# Development of a scoring method to visually score cortical interruptions on high-resolution peripheral quantitative computed tomography in rheumatoid arthritis and healthy controls

**DOI:** 10.1371/journal.pone.0200331

**Published:** 2018-07-09

**Authors:** Andrea Scharmga, Michiel Peters, Joop P. van den Bergh, Piet Geusens, Daan Loeffen, Bert van Rietbergen, Thea Schoonbrood, Debby Vosse, René Weijers, Astrid van Tubergen

**Affiliations:** 1 Department of Rheumatology, Maastricht University Medical Center, Maastricht, The Netherlands; 2 NUTRIM School of Nutrition and Translational Research in Metabolism, Maastricht University, Maastricht, The Netherlands; 3 CAPHRI Care and Public Health Research Institute, Maastricht University, Maastricht, The Netherlands; 4 Department of Internal Medicine, Viecuri Medical Center, Venlo, The Netherlands; 5 Faculty of Medicine and Life Sciences, Hasselt University, Hasselt, Belgium; 6 Department of Radiology, Maastricht University Medical Center, Maastricht, The Netherlands; 7 Department of Medical Engineering, Eindhoven University of Technology, Eindhoven, The Netherlands; 8 Department of Orthopaedic surgery, Maastricht University Medical Center, Maastricht, The Netherlands; University of Twente, NETHERLANDS

## Abstract

**Objectives:**

To develop a scoring method to visually score cortical interruptions in finger joints on High-Resolution peripheral Quantitative Computed Tomography (HR-pQCT), determine its intra- and inter-reader reliability and test its feasibility.

**Methods:**

The scoring method was developed by integrating results from in-depth discussions with experts, consensus meetings, multiple reading experiments and the literature. Cortical interruptions were scored by two independent readers in an imaging dataset with finger joints from patients with rheumatoid arthritis (RA) and healthy controls and assessed for adjacent trabecular distortion. Reliability for the total number of cortical interruptions per joint and per quadrant was calculated using intraclass correlation coefficient (ICC). Feasibility was tested by recording the time to analyze one joint.

**Results:**

In 98 joints we identified 252 cortical interruptions, 17% had trabecular distortion. Mean diameter of the interruptions was significantly larger in patients with RA compared with healthy controls (0.88 vs 0.47 mm, p = 0.03). Intra-reader reliability was ICC 0.88 (95% CI 0.83;0.92) per joint and ICC 0.69 (95% CI 0.65;0.73) per quadrant. Inter-reader reliability was ICC 0.48 (95% CI 0.20;0.67) per joint and ICC 0.56 (95% CI 0.49;0.62) per quadrant. The time to score one joint was mean 9.2 (SD 4.9) min.

**Conclusions:**

This scoring method allows detection of small cortical interruptions on HR-pQCT imaging of finger joints, which is promising for use in clinical studies.

## Introduction

High-Resolution peripheral Quantitative Computed Tomography (HR-pQCT) is a non-invasive imaging technique enabling three dimensional analysis of bone microarchitecture at an isotropic voxel size of 82 microns and a spatial resolution of 130 microns. HR-pQCT has the potential to identify and quantify early bone changes in metacarpophalangeal (MCP) joints before bone damage can be identified on radiographs [1]. With HR-pQCT, very small cortical interruptions of less than 0.5 mm can be detected [1,2]. These cortical interruptions can be physiological, e.g. vascular channels or pathological, e.g. erosions in rheumatic diseases [3]. A definition for detection of a vascular channel on HR-pQCT has been proposed [3], but appeared insufficient [4]. Vascular channels were more heterogeneous and smaller in size than previously suggested. Also several definitions for erosion on HR-pQCT exist [3]. In one study cortical interruptions greater than 1.9 mm were considered bone erosions specific for rheumatoid arthritis (RA) [1]. To date, a validated visual scoring method for HR-pQCT images that incorporates all cortical interruptions, including vascular channels and erosions, is lacking. Such a scoring system could aid in making a distinction between (small) physiological vascular channels and pathological erosions, which may be accompanied by adjacent trabecular distortion [3].

Here we describe the development of a scoring method to visually score cortical interruptions on HR-pQCT. We integrated results from in-depth discussions with experts, consensus meetings, multiple reading experiments [4,5], and the literature on an image grading scale for motion artefacts [6] and joint assessment on HR-pQCT images as described by Stach et al [1]. Furthermore, we tested its intra- and inter-reader reliability, and feasibility in terms of time to perform.

## Methods

### Subjects

A representative sample of 30 subjects (10 healthy controls and 20 patients with RA) from 38 female healthy controls and 41 female patients diagnosed with RA, participating in an observational cohort study, the MOSA-Hand study, was used [7]. All patients with RA fulfilled the 2010 American College of Rheumatology (ACR)/European League Against Rheumatism (EULAR) classification criteria for RA [8]. Healthy controls, matched per decade, did not suffer from hand joint complaints. All subjects were recruited at the Maastricht University Medical Centre, the Netherlands and signed informed consent. Ethical approval was obtained from the ethics board of the academic hospital Maastricht/ Maastricht University, the Netherlands. Netherlands Trial Registry number: NTR3821.

### Conventional radiography

Posterior-anterior radiographs of both hands were taken from all subjects at baseline. Two experienced rheumatologists (TS and DV) independently scored the radiographs according to the Sharp/ van der Heijde method (SvdH) for the presence of joint damage in hand joints (range 0–280) [9]. Radiographs were scored using a free Digital Imaging and Communications in Medicine (DICOM) viewer (Osirix v.5.8.5 64-bit). The readers were blinded for demographic and clinical data. A subsample of 30 subjects was selected, that most represented the entire spectrum of radiographic damage according to the SvdH scores, ranging from none to severe, instead of the whole sample that could possibly contain most patients on one side of the spectrum. Healthy controls were selected when the SvdH score was zero.

### HR-pQCT image acquisition

Second and third MCP and proximal interphalangeal (PIP) joints were scanned with HR-pQCT (XtremeCT1, Scanco Medical AG, Switzerland) according to the HR-pQCT imaging protocol proposed by The Study grouP for xtrEme Computed Tomography in Rheumatoid Arthritis (SPECTRA) at baseline and after one year [10]. In patients with RA, both hands were scanned and in healthy controls only the dominant hand. Each hand was scanned using the standard carbon forearm cast as provided by the manufacturer. Scanning time per patient was nine minutes for MCP joints (three stacks of 9.02 mm, 330 slices) and six minutes for PIP joints (two stacks of 9.02 mm, 220 slices).

### Development of the scoring method

We developed a scoring method to visually score cortical interruptions on HR-pQCT images. In monthly meetings during a 1 year period, a consensus was developed on the visual scoring of cortical interruptions by a panel of rheumatologists, radiologists and engineers with particular interest and expertise in image analyses. We integrated results from in-depth discussions with experts, multiple reading experiments and comparison with microCT and histology [4,5] and from the literature available studies on the grading of motion artefacts [6] and on the assessment of finger joints using HR-pQCT images [1].

**Fig 1:**
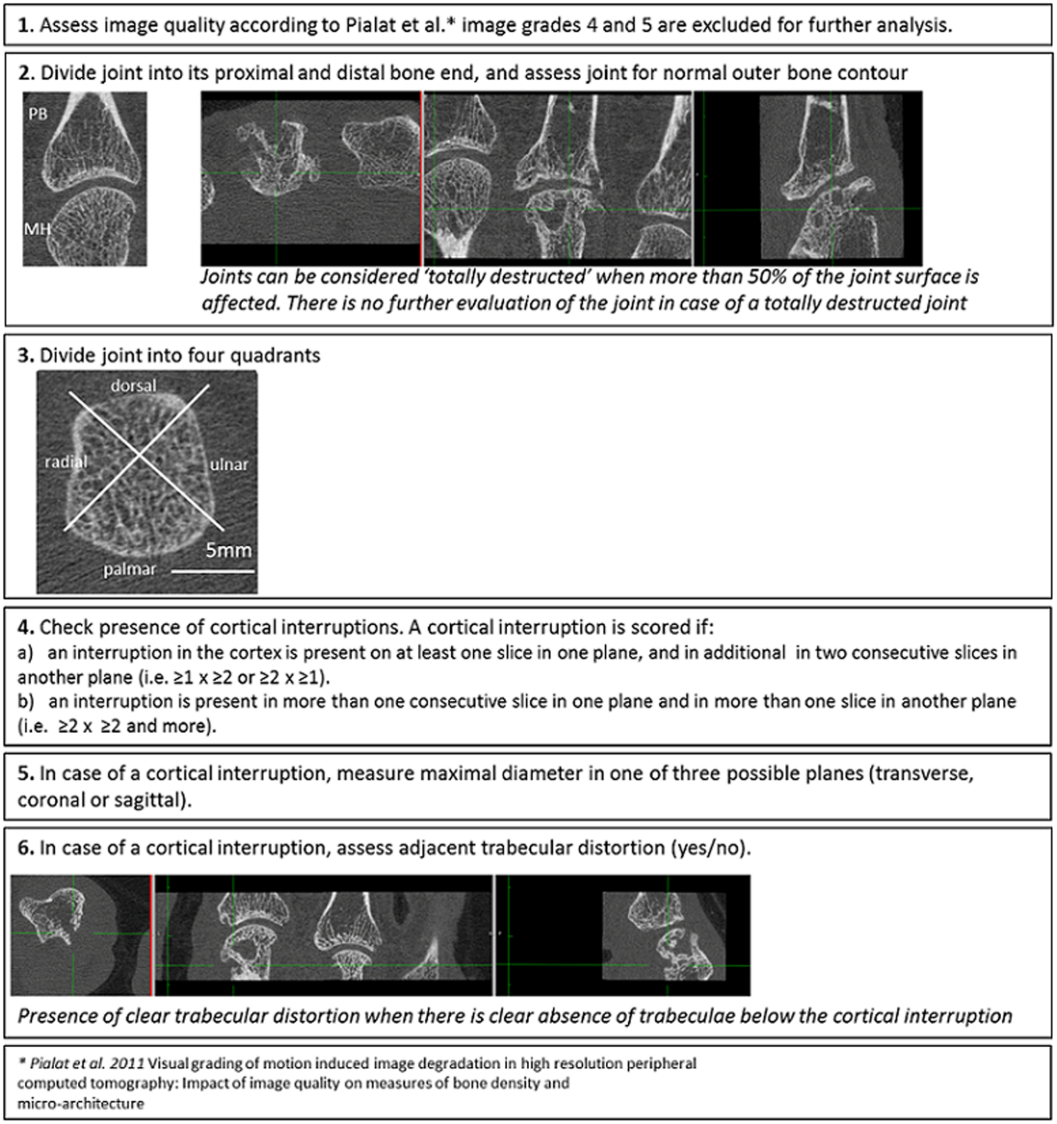
Steps of the visual scoring method.

Our proposal for the visual scoring method is shown in Fig 1.

First, images need to be assessed for the presence of motion artifacts according to Pialat et al. [6]. Only HR-pQCT images with image quality grades 1, 2 or 3 are considered evaluable, and image quality grades 4 and 5 with severe and extreme motion artifacts are excluded from further analysis. Second, each joint is divided into its proximal and distal bone end and it is assessed whether a normal outer bone contour is present. When more than 50% of the joint surface of the cortical bone per bone end is destroyed, the bone end is considered ‘totally destructed’ and excluded from further analysis. Third, each bone end is further divided into four quadrants (palmar, ulnar, dorsal and radial) according to Stach et al [1]. Thus per joint, eight quadrants are assessed for the presence of cortical interruptions [1]. Fourth, a cortical interruption is scored when it fulfilled one of the following criteria: a). a cortical interruption in at least one slice in one plane and in addition in two consecutive slices in another plane (i.e. ≥1 x ≥2 or ≥2 x ≥1 slices), b). a cortical interruption in more than one consecutive slice in one plane and in more than one slice in another plane (e.g. ≥2 x ≥2 slices). Fifth, when a cortical interruption is identified, its maximal diameter is measured (in mm) in one of the three planes (transverse, coronal or sagittal). Sixth, the adjacent trabecular structure is assessed for distortion, defined as clear absence of calcified trabeculae underlying the cortical interruption.

### Image analyses

The HR-pQCT images were viewed in Osirix. Using the visual scoring method, two experienced readers (AS and MP) with extensive experience in visual image analysis on HR-pQCT and microCT [5] prior to the start of this study and who were formally trained by SPECTRA, scored the HR-pQCT images twice, blinded for demographic and clinical data, and independently of each other. When large discrepancies (difference of >3 cortical interruptions per joint) between the readers were found, these images were checked and evaluated for reasons for discrepancy. Scores were not adjusted after this evaluation.

### Reliability

For testing the reliability of the visual scoring method, second and third MCP and PIP joints were used. In the dataset, each healthy control contributed with two joints, one MCP and one PIP joint. Each patient with RA contributed with four joints, two MCP and two PIP joints which were randomly selected from either hand.

### Feasibility

For testing the feasibility of the visual scoring method, start and end time of reading one joint was recorded in each subject in full round minutes, for each reader separately.

### Statistics

Descriptive statistics were used to calculate the SvdH score on conventional radiographs (CR), and to analyze the number, diameter of cortical interruptions and presence of trabecular distortion, and the average time to score a joint on HR-pQCT using the visual scoring method. Wilcoxon signed-rank test was used to compare the number of cortical interruptions of the first and second reading on HR-pQCT for all subjects and for intra-reader scores. Mann-Whitney U test was used to compare the diameter of the cortical interruptions between healthy controls and patients with RA. Intra- and inter-reader reliability of the visual scoring method was assessed using Cohen’s kappa (κ) based on the presence or absence of a cortical interruption per quadrant and per joint. Intraclass correlation coefficient (ICC), based on a two way random effects model, was used to assess inter-reader reliability of the SvdH scores, and of intra- and inter-reader reliability of HR-pQCT image scores based on the total number of cortical interruptions per quadrant and per joint. Reliability was rated according to Landis et al.: <0.00 poor, 0.00–0.20 slight, 0.21–0.40 fair, 0.41–0.60 moderate, 0.61–0.80 substantial, 0.81–1.00 almost perfect [11]. Totally destructed bone ends (base and/or head) were excluded from the analyses on HR-pQCT. Only quadrants without motion artefacts on both readings were taken into account for the intra-reader analysis.

## Results

**Fig 2:**
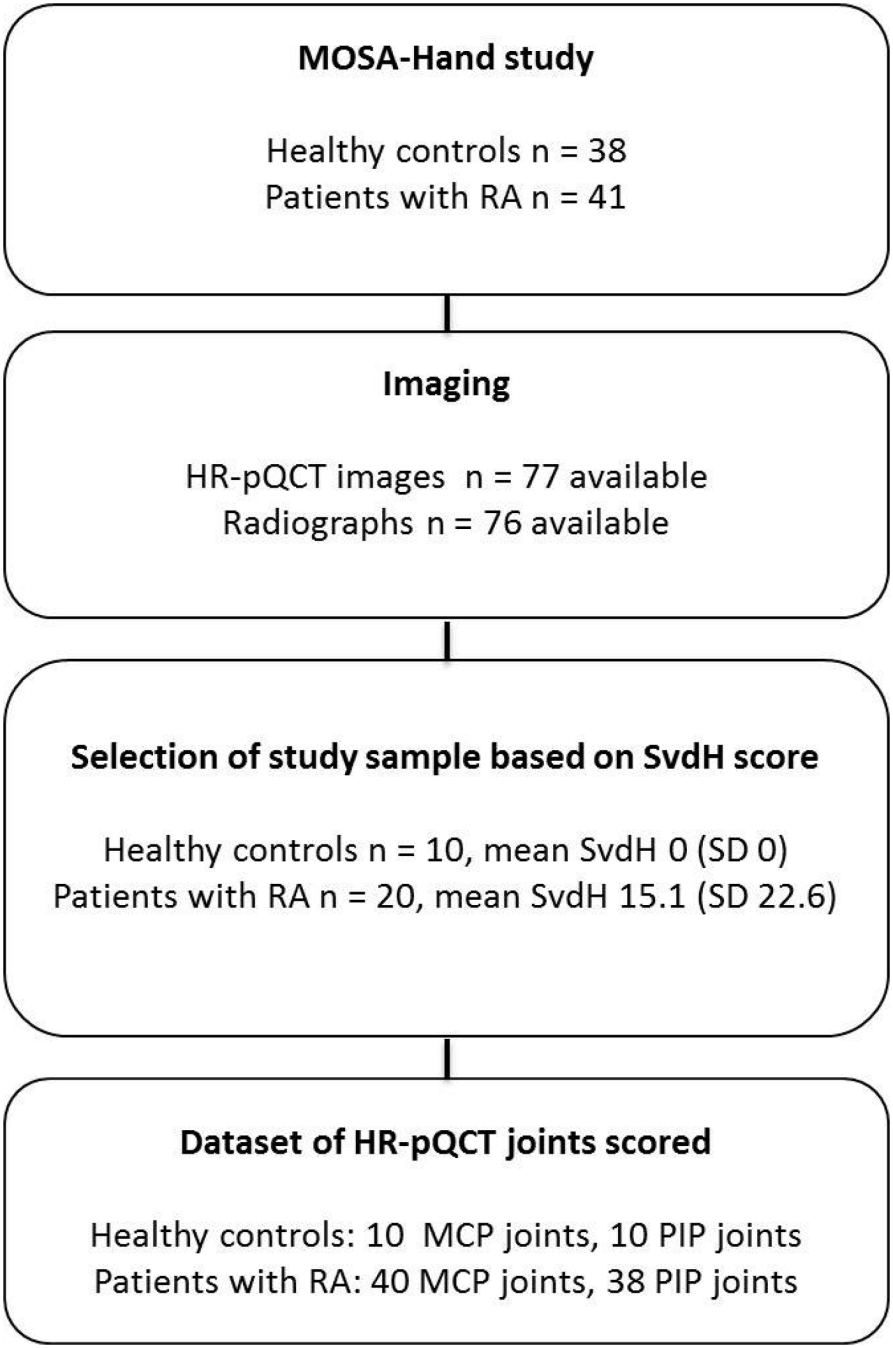
Flowchart dataset of HR-pQCT joints scored from the MOSA-Hand study. Abbrevations: HR-pQCT; high resolution peripheral quantitative computed tomography, SvdH; Sharp/ van der Heijde method.

Fig 2 shows a flowchart of subject inclusion in the MOSA-Hand study and selection of the sample of 30 female subjects (10 healthy controls and 20 patients with RA) for this study. The mean age was 46.1 (SD 9.2) years for the selected healthy controls and 57.4 (SD 5.8) years for the selected patients with RA. The mean disease duration for patients with RA was 117.1 (SD 110.1, range 4–334) months. In one patient, the HR-pQCT images of two PIP joints were missing because of intolerance to long immobilization during scanning. The dataset of HR-pQCT images to examine the reliability and feasibility of the visual method included in total 98 joints: 50 MCP (10 from healthy controls and 40 from patients with RA) and 48 PIP joints (10 from healthy controls and 38 from patients with RA).

### Conventional radiography

ICC between the SvdH scores of the two readers was ICC 0.96 (95% CI 0.93–0.97). Mean SvdH scores for the 30 selected subjects were 0 (SD 0) for healthy controls and 15.1 (SD 22.6; range 0–87.5) for patients with RA.

### Image analysis

Table 1 shows descriptives of the first and second reading of the dataset on HR-pQCT. In the first readings of Reader 1 and Reader 2, 40 out of 784 quadrants were not considered evaluable due to motion grade >3, 32 quadrants were considered to be totally destructed by Reader 1 and 42 by Reader 2. In total, 702 quadrants by Reader 1 and 706 quadrants by Reader 2 were further assessed for the presence of a cortical interruption fulfilling the criteria of the scoring method. Reader 1 identified 252 cortical interruptions, of which 35 were small (in at least 1x2 slices) and 217 were identified in 2x2 or more consecutive slices. Reader 2 scored a significantly higher number of cortical interruptions than Reader 1 in the first and second reading (first reading: 381 versus 252 cortical interruptions, second reading: 456 versus 248 cortical interruptions, both p<0.001). Intra-reader scores for the total number of cortical interruptions were not significantly different for Reader 1 (252 versus 248, p = 0.67), but were significantly different for Reader 2 (381 versus 456, p<0.001). Adjacent trabecular distortion was observed in 17% (Reader 1) and 25% (Reader 2) of the cortical interruptions (Table 1). The diameter of the cortical interruptions ranged from 0.09 mm to 7.4 mm for Reader 1 and from 0.11 mm to 7.88 mm for Reader 2 (Table 1).

**Table 1:** Descriptives of first and second reading of Reader 1 and 2.

	Reader 1		Reader 2	
	First reading	Second reading	First reading	Second reading
Number of evaluable quadrants (%)	734 (94%)	744 (95%)	728 (93%)	730 (93%)
Number of totally destructed quadrants	32	36	32	32
Number of quadrants further assessed	702 (90%)	708 (90%)	696 (89%)	698 (89%)
Number of joints further assessed	93	93	93	95
Number of interruptions	252	248	381	456
Mean per joint (SD)	2.7 (2.3)	2.6 (2.2)	4.1 (3.3)	4.8 (3.2)
present on < 2 consecutive slices	35	45	1	0
present on ≥ 2 consecutive slices	217	203	380	456
with trabecular distortion (%)	44 (17%)	60 (25%)	94 (25%)	97 (21%)
Mean diameter of cortical interruption in mm (SD), range	0.82 (0.91) 0.12–7.16	0.73 (0.89) 0.09–7.4	0.68 (0.71) 0.13–7.88	0.63 (0.62) 0.11–4.57
Mean reading time per joint in minutes, (SD), range	8.3 (3.7) 1–18	8.8 (3.9) 1–20	9.8 (6.9) 1–38	9.9 (5.4) 1–27

Table 2 shows the results of the visual scoring for healthy controls and patients with RA separately, based on results of the first reading of Reader 1. Most interruptions were seen in two or more than two consecutive slices in two or more planes (≥2 x ≥2 planes) in both healthy controls and patients with RA (n = 34 (89%) and n = 183 (85.6%), respectively). None of the cortical interruptions in healthy controls were accompanied by adjacent trabecular distortion, but in patients with RA 44 (21%) of the cortical interruptions were accompanied by adjacent trabecular distortion. The mean diameter of the interruptions was significantly larger in patients with RA compared with healthy controls (0.88 vs 0.47 mm, p = 0.03).

**Table 2:** Descriptives of cortical interruptions in healthy controls and patients with RA.

	Healthy controls (n = 20 joints)	Patients with RA (n = 78 joints)
Number of interruptions	38	214
Mean number of cortical interruptions per joint (SD)	1.9 (1.7)	2.9 (2.4)
present on < 2 consecutive slices(%)	4 (11%)	31 (14.4%)
present on ≥ 2 consecutive slices (%)	34 (89%)	183 (85.6%)
with trabecular distortion (%)	0 (0%)	44 (21%)
Mean diameter of cortical interruption in mm, (SD), range	0.47 (0.22) 0.19–1.11	0.88 (0.97) 0.12–7.16
Mean reading time per joint in minutes, (SD), range	7.4 (3.2) 3–14	8.5 (3.7) 1–18

Numbers based on results Reader 1, first reading

Abbreviations: RA; rheumatoid arthritis

Online S1 Table shows the mean number of cortical interruptions per quadrant in healthy controls and patients with RA, based on results of the first reading of Reader 1. Significantly more cortical interruptions were observed in the ulnar and radial quadrants in patients with RA compared with healthy controls (p<0.05, S1 Table). Cortical interruptions were also frequently seen in the palmar quadrants, but equally in patients with RA and healthy controls. Cortical interruptions with adjacent trabecular distortion in patients with RA were most frequently seen in the ulnar quadrant (n = 17), followed by palmar, radial and dorsal quadrants (n = 9, n = 8 and n = 5 respectively).

**Fig 3:**
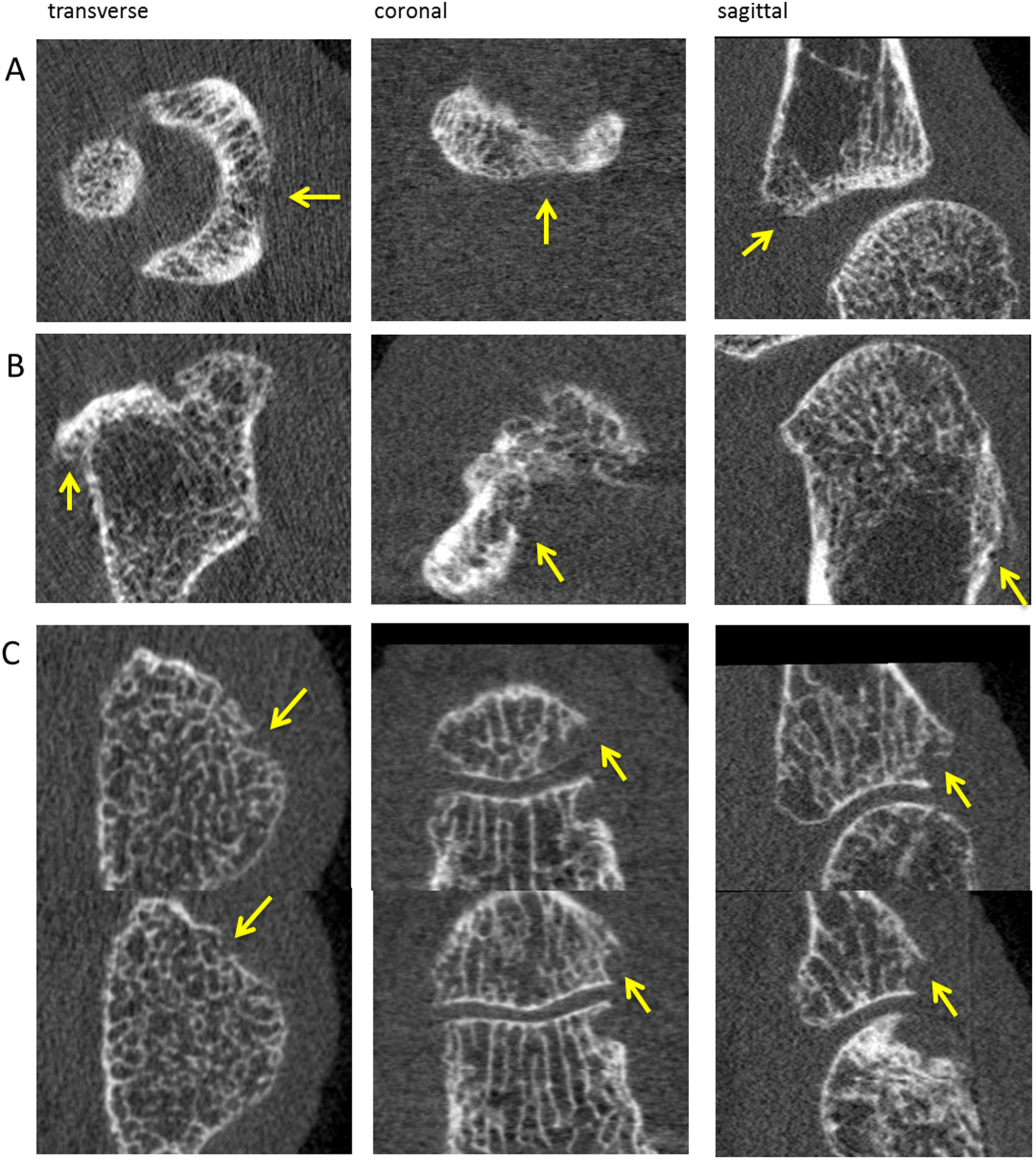
HR-pQCT images of cases leading to discrepancies between readers in scoring. Panel A: low density caused by low mineralization and/or thin cortex. Panel B: bone apposition. Panel C: multiple breaks belonging to one large break at the base of the phalanx. Abbrevations: HR-pQCT; high resolution peripheral quantitative computed tomography.

In total 13 joints were re-evaluated because of discrepancies of >3 interruptions per joint between the two readers in the first reading. Discrepancies occurred only in patients with RA. In general, Reader 1 was more conservative than Reader 2 on the identification of an interruption. Fig 3, panel A shows an example of a discrepancy in the observation of a possible cortical interruption. The interruption is difficult to distinguish from the background noise, due to low density caused by low mineralization and/or a thin cortex. Furthermore, larger interruptions showed more agreement than smaller interruptions. Also, irregularities in the cortex or bone apposition (Fig 3, panel B) caused discrepancies in the total number of interruptions. Exceptionally, an interruption was overlooked by one reader, multiple smaller interruptions were counted instead of one large interruption (Fig 3, panel C) or, in retrospect, an interruption was wrongly considered a cortical interruption, but did not fulfill the criteria.

### Reliability

Table 3 shows the results for intra- and inter-reader reliability of the visual scoring method for the number of cortical interruptions based on joints (n = 98) and quadrants (n = 784). Intra-reader reliability of Reader 1 and Reader 2 was moderate (0.52 to 0.67) for the presence of an interruption, but substantial to almost perfect (0.69 to 0.88) for the number of cortical interruptions (Table 3). Inter-reader reliability was fair to moderate (0.37 to 0.56) for the presence and total number of cortical interruptions (Table 3).

**Table 3:** Intra- and inter-reader reliability of the visual scoring method.

	Intra-reader				Inter-reader (reading 1)	
	Reader 1		Reader 2		κ	ICC
	κ	ICC	κ	ICC		
Cortical interruptions based on joints	0.52 (0.26;0.79)	0.88 (0.83;0.92)	0.56 (0.07;1.00)	0.79 (0.66;0.87)	0.37 (0.02;0.72)	0.48 (0.20;0.67)
Cortical interruptions based on quadrants	0.63 (0.56;0.69)	0.69 (0.65;0.73)	0.67 (0.62;0.72)	0.76 (0.72;0.79)	0.53 (0.47;0.60)	0.56 (0.49;0.62)

Values represent Cohen’s kappa (κ) or intraclass correlation coefficient (ICC) with 95% confidence interval. κ is calculated for the presence of a cortical interruption, ICC is calculated for the total amount of cortical interruptions.

### Feasibility

The mean time to score one joint was 8.6 min (SD 3.7) for Reader 1 and 10.0 min (SD 6.0) for Reader 2 (Table 1), the mean for both readers was 9.2 min (SD 4.9).

## Discussion

We developed a visual scoring method to detect cortical interruptions on HR-pQCT imaging in healthy controls and patients with RA and tested its intra- and inter-reader reliability and feasibility. Cortical interruptions were seen in both healthy controls and patients with RA, but more frequently and with a larger diameter in patients with RA. We showed that most cortical interruptions (86%) were seen in ≥2 x ≥2 slices and 21% of the cortical interruptions had adjacent trabecular distortion. Although intra-reader reliability was moderate to almost perfect, the inter-reader reliability was fair to moderate for the presence and total number of cortical interruptions. Scoring one joint took on average less than ten minutes, but the longest scoring time recorded was 38 minutes.

Different methods to investigate and interpret HR-pQCT images of mainly erosions have been proposed [1,2,3,12]. Fouque-Aubert et al. defined erosions as sharply demarcated bone lesions with juxta-articular localisation with a cortical interruption seen in at least two adjacent slices in one plane and assigned a score by percentage of bone volume involved (score 0–10, by 10% volume increments) [12]. Stach et al. defined erosions as a clear juxta-articular interruption within the cortical shell, but did not further specify the number of adjacent slices or orthogonal planes [1]. Erosions were graded (grades 0–3) based on the maximum diameter of the cortical interruption. In addition, three-dimensional reconstructions of the joint were made to assess cortical surface change [1]. Srikhum et al. defined erosions as sharply demarcated juxta-articular focal bone lesions with a cortical interruption in at least two adjacent slices in one plane, and also graded erosions (grades 0–3) based on the maximal dimension of the cortical interruption [2]. The SPECTRA collaboration proposed a case definition for erosion described as a cortical interruption extending over a minimum of two consecutive slices in two perpendicular planes, and measured erosion by maximum width and perpendicular to the width, the maximum depth of the erosion [3]. A limitation of these studies is that they specifically aimed at scoring established bone erosions in RA which, in most studies, need to be seen on two consecutive slices. As a result, only large interruptions were scored [2], small cortical interruptions were not taken into account. Therefore, physiological vascular channels, which might represent starting points for erosion development [13,14,15], were not identified. However, the strength of HR-pQCT imaging is that it has the potential to identify small (i.e. early) bone changes, also in the vascular channels. Our visual scoring method incorporates all cortical interruptions in MCP and PIP joints and hereby allows to make a distinction between small cortical interruptions and larger cortical interruptions which are considered more specific for RA [1,3].

Previous studies in patients with RA showed a predilection for large cortical interruptions (erosions) at the radial quadrant [1,16]. Finzel et al. suggested that the palmar quadrant is the site where physiological vascular channels enter the cortex [16]. We observed most cortical interruptions in the palmar quadrant in healthy controls and in the palmar and radial quadrant in patients with RA. However, our method did not categorize cortical interruptions into vascular channels or erosions. We assessed adjacent trabecular distortion and observed that this was only observed in patients with RA (21%), with the highest frequency in the ulnar quadrant. Both radial and ulnar quadrants are insertion sites of collateral ligaments and considered an area that is prone for erosion development [17].

The inter-reader reliability of our scoring method was fair to moderate, however, a significant difference in the total number of cortical interruptions between the two readers was found. Several reasons for this difference were observed: smaller interruptions showed less agreement, multiple smaller interruptions were counted as one large interruption by one reader whereas the other reader counted each, and disagreement was sometimes caused by low density of the cortex. These discrepancies were also observed in a previous study from our study group, in which we used microCT as gold standard [18]. We found fair to substantial reliability of HR-pQCT in the detection of cortical interruptions on two consecutive slices in two planes. Other studies that tested methods for scoring bone erosions on HR-pQCT showed moderate to almost perfect reliability [1,2,3]. The lower reliability values observed in our study are most likely attributable to the smaller cortical interruptions which we incorporated in the visual scoring method. Reliability scores for other imaging techniques, such as conventional radiographs, also show a wide range in reliability, from moderate to almost perfect (0.47–1.00), despite being scored by experienced readers [19]. Visual interpretation of images remains a complex task [20]. Furthermore, scoring of the HR-pQCT images was time consuming with a mean time to score one joint of 9.2 min. Only one other study reported scoring time for MCP joints; they found a median scoring time of 2 minutes (range 1.20–5.30 minutes) [2]. With a scoring time per joint of up to 38 minutes, our visual scoring method is barely feasible for clinical practise. On the other hand, with our visual scoring method, every cortical interruption can be scored, which increases the time to score per joint and hence lowers the feasibility. This might improve when scoring only interruptions with adjacent trabecular distortion, i.e. those suspected for reflecting an erosion [1,3]. Alternatively, automated algorithms to score the cortical interruptions could be a solution. A study from our group has shown that a (semi) automated algorithm was highly reproducible in the detection of cortical interruptions (ICC 0.93 (95% CI 0.87;0.97)) [21].

The present study has an important limitation. The visual scoring method aimed to score cortical interruptions and to assess the presence of adjacent trabecular bone structure. The presence of adjacent trabecular bone is however an underestimation of the damage present, since totally destructed bone ends were by definition excluded from further assessment. The choice to exclude bone ends when more than 50% of the bone surface was destroyed was made based on the presence of multiple (large) indistinguishable interruptions. The goal of the visual scoring method is, however, to also detect small cortical interruptions and ultimately, to aid in making a distinction between physiological and pathological interruptions. To support such hypothesis, longitudinal studies will be needed.

In conclusion, this scoring method allows detection of small cortical interruptions on HR-pQCT imaging of finger joints. Although reading was time-consuming, this tool is promising for use in clinical studies.

## Supporting information

S1 File Scoringsheet first reading Reader 1. (PDF)

S2 File Scoringsheet second reading Reader 1. (PDF)

S3 File Scoringsheet first reading Reader 2. (PDF)

S4 File Scoringsheet second reading Reader 2. (PDF)

S1 Table Mean (SD) number of cortical interruptions based on quadrants in healthy controls and patients with RA. (DOCX)
