# Supplementary material for: Development of a scoring method to visually score cortical interruptions on high-resolution peripheral quantitative computed tomography in rheumatoid arthritis and healthy controls
Source: PLoS One. 2018 Jul 9;13(7):e0200331. doi: 10.1371/journal.pone.0200331 (PMC6037386; doi:10.1371/journal.pone.0200331)
Supplement: S1 File — (PDF) [file pone.0200331.s001.pdf]

Reader initials	studyID	subjectID	joint (0=MCP2, 1= MCP3, 2=PIP2, 3=PIP3)	hand 1=rechts 2= links	surface (0= palmar PB, 1=u	evaluable (0=no 1=yes)	discontinuity (0=no 1=yes 2=total destruction)	x value	y value	z value	planes	adjacent trabecular distortion (0=no 1=yes)	parallel structure (0=nc	maximal diameter
AS	1200	22	0	1	0	1	1	83,08	46,72	94,5	3x4	0	1	0,467
AS	1200	22	0	1	1	1	1	77,18	50,09	97,12	4x5	0	0	0,768
AS	1200	22	0	1	2	1	1	88,67	36,74	95,56	7x4	0	0	1,646
AS	1200	22	0	1	3	1	1	90,47	41,23	93,51	3x2	0	1	0,226
AS	1200	22	0	1	3	1	1	90,45	44,99	96,06	13x17	0	0	1,53
AS	1200	22	0	1	4	1	0							
AS	1200	22	0	1	5	1	1	77,73	35,89	101,63	47x30	0	0	5,48
AS	1200	22	0	1	6	1	1	80,95	30,31	102,78	4x8	0	0	1,306
AS	1200	22	0	1	7	1	1	89,69	35,55	106,47	35x20	1	0	2,83
AS	1200	22	0	1	7	1	1	86,85	32,9	100,65	9x8	1	0	1,564
AS	1201	30	0	2	0	1	0							
AS	1201	30	0	2	1	1	0							
AS	1201	30	0	2	2	1	0							
AS	1201	30	0	2	3	1	0							
AS	1201	30	0	2	4	1	0							
AS	1201	30	0	2	5	1	0							
AS	1201	30	0	2	6	1	1	46,72	44,68	96,9	1x2	0	1	0,31
AS	1201	30	0	2	7	1	1	40,21	50,74	100,02	2x3	0	0	0,465
AS	1202	61	2	1	8	1	1	82,48	55,08	36,24	7x6	0	0	0,73
AS	1202	61	2	1	8	1	1	79,86	55,88	36,32	3x2	0	1	0,302
AS	1202	61	2	1	9	1	0							
AS	1202	61	2	1	10	1	0							
AS	1202	61	2	1	11	1	1	85,84	53,25	31,48	2x1	0	1	0,454
AS	1202	61	2	1	12	1	0							
AS	1202	61	2	1	13	1	1	75,35	52,36	40,26	2x1	0	0	0,256
AS	1202	61	2	1	14	1	0							
AS	1202	61	2	1	15	1	0							
AS	1204	22	1	2	0	1	1	62,23	53,26	92,73	2x3	0	1	0,276
AS	1204	22	1	2	0	1	1	64,65	53,73	93,3	3x4	0	1	0,535
AS	1204	22	1	2	0	1	1	62,79	53,64	95,6	15x22	1	0	2,138
AS	1204	22	1	2	1	1	0							
AS	1204	22	1	2	2	1	0							
AS	1204	22	1	2	3	1	0							
AS	1204	22	1	2	4	1	0							
AS	1204	22	1	2	5	1	1	66,8	51,15	104,37	12x15	0	0	1,299
AS	1204	22	1	2	5	1	1	65,87	45,56	101,42	4x5	0	0	0,328
AS	1204	22	1	2	6	1	0							
AS	1204	22	1	2	7	1	1	54,81	48,65	106,51	11x10	1	0	1,096
AS	1204	22	1	2	7	1	1	56,32	45,25	105,11	10x10	1	0	1,258
AS	1205	30	3	1	8	1	0							
AS	1205	30	3	1	9	1	0							
AS	1205	30	3	1	10	1	0							
AS	1205	30	3	1	11	1	0							
AS	1205	30	3	1	12	1	0							
AS	1205	30	3	1	13	1	1	51,21	53,57	39,25	7x9	0	0	0,901
AS	1205	30	3	1	14	1	1	52,68	48,8	39,17	2x2	0	1	0,247
AS	1205	30	3	1	15	1	1	62,19	52,2	41,3	11x9	1	0	0,699
AS	1206	61	3	2	8	1	1	67,45	57,39	31,28	1x2	0	1	0,292
AS	1206	61	3	2	9	1	0							
AS	1206	61	3	2	10	1	0							
AS	1206	61	3	2	11	1	0							
AS	1206	61	3	2	12	1	0							
AS	1206	61	3	2	13	1	0							
AS	1206	61	3	2	14	1	0							
AS	1206	61	3	2	15	1	0							
AS	1207	30	1	1	0	1	1	53,26	47,94	70,03	2x1	0	1	0,298
AS	1207	30	1	1	0	1	1	54,53	48,52	72,24	3x1	0	1	0,19
AS	1207	30	1	1	0	1	1	59,37	49,65	76,59	1x2	0	1	0,294
AS	1207	30	1	1	0	1	1	56,01	49,31	76,75	3x1	0	1	0,371
AS	1207	30	1	1	1	1	0							
AS	1207	30	1	1	2	1	0							
AS	1207	30	1	1	3	1	0							
AS	1207	30	1	1	4	1	0							
AS	1207	30	1	1	5	1	0							
AS	1207	30	1	1	6	1	0							
AS	1207	30	1	1	7	1	1	62,31	41,17	87,41	8x6	0	0	1,149
AS	1208	61	1	2	0	1	0							
AS	1208	61	1	2	1	1	0							
AS	1208	61	1	2	2	1	0							
AS	1208	61	1	2	3	1	0							
AS	1208	61	1	2	4	1	0							
AS	1208	61	1	2	5	1	0							
AS	1208	61	1	2	6	1	0							
AS	1208	61	1	2	7	1	0							
AS	1210	56	3	2	8	1	0							
AS	1210	56	3	2	9	1	0							
AS	1210	56	3	2	10	1	0							
AS	1210	56	3	2	11	1	0							
AS	1210	56	3	2	12	1	0							
AS	1210	56	3	2	13	1	1	71,49	55,11	49,08	27x3	0	0	1,122
AS	1210	56	3	2	14	1	0							
AS	1210	56	3	2	15	1	0							
AS	1211	22	2	1	8	0								
AS	1211	22	2	1	9	0								
AS	1211	22	2	1	10	0								
AS	1211	22	2	1	11	0								
AS	1211	22	2	1	12	0								
AS	1211	22	2	1	13	0								
AS	1211	22	2	1	14	0								
AS	1211	22	2	1	15	0								
AS	1212	30	2	2	8	1	0							
AS	1212	30	2	2	9	1	0							
AS	1212	30	2	2	10	1	0							
AS	1212	30	2	2	11	1	0							
AS	1212	30	2	2	12	1	0							
AS	1212	30	2	2	13	1	0							
AS	1212	30	2	2	14	1	0							
AS	1212	30	2	2	15	1	0							
AS	1213	22	3	2	8	1	0							
AS	1213	22	3	2	9	1	0							
AS	1213	22	3	2	10	1	0							
AS	1213	22	3	2	11	1	0							
AS	1213	22	3	2	12	0								
AS	1213	22	3	2	13	0								
AS	1213	22	3	2	14	0								
AS	1213	22	3	2	15	0								
AS	1214	61	0	1	0	1	1	79,42	42,2	72,06	2x2	0	1	0,316
AS	1214	61	0	1	0	1	1	78,47	42,19	73,13	3x4	0	1	0,503
AS	1214	61	0	1	1	1	0							
AS	1214	61	0	1	2	1	0							
AS	1214	61	0	1	3	1	0							
AS	1214	61	0	1	4	1	1	79,53	39,17	84,28	2x3	0	1	0,486
AS	1214	61	0	1	5	1	0							
AS	1214	61	0	1	6	1	1	73,87	28,7	82,72	2x1	0	1	0,525
AS	1214	61	0	1	7	1	0							
AS	1215	62	0	2	0	1	0							
AS	1215	62	0	2	1	1	0							
AS	1215	62	0	2	2	1	0							
AS	1215	62	0	2	3	1	1	35,46	42,35	68,95	13x9	0	0	1,721
AS	1215	62	0	2	4	1	1	40,95	36,72	76,74	2x2	0	0	0,51
AS	1215	62	0	2	4	1	1	39,03	37,6	75,84	1x2	0	1	0,314
AS	1215	62	0	2	5	1	1	46,44	32,28	74,85	2x4	0	1	0,318
AS	1215	62	0	2	6	1	0							
AS	1215	62	0	2	7	1	1	36,84	32,92	77,31	20x42	1	0	3,019
AS	1216	31	2	1	8	1	1	85,29	55,33	52,11	2x1	0	1	0,465
AS	1216	31	2	1	8	1	1	85,88	55,03	53,51	2x4	0	0	0,862
AS	1216	31	2	1	8	1	1	79,65	56,1	51,38	2x2	0	1	0,439
AS	1216	31	2	1	9	1	0							
AS	1216	31	2	1	10	1	0							
AS	1216	31	2	1	11	1	1	88,42	48,79	51,29	2x3	0	1	0,289
AS	1216	31	2	1	12	1	0							
AS	1216	31	2	1	13	1	0							
AS	1216	31	2	1	14	1	0							
AS	1216	31	2	1	15	1	0							
AS	1217	57	2	2	8	1	1	44,2	54,97	37,15	4x2	0	1	0,19
AS	1217	57	2	2	9	1	0							
AS	1217	57	2	2	10	1	0							
AS	1217	57	2	2	11	1	1	36,26	50,66	38,88	2x2	0	1	0,203
AS	1217	57	2	2	12	1	0							
AS	1217	57	2	2	13	1	1	45,75	52,06	47,08	5x5	0	1	0,584
AS	1217	57	2	2	14	1	1	37,22	47,14	48,47	2x1	0	1	0,236
AS	1217	57	2	2	15	1	1	35,38	54,19	48,47	15x12	0	0	0,836
AS	1217	57	2	2	15	1	1	35,37	51,51	45,52	16x11	0	0	0,743
AS	1220	67	3	2	8	1	0							
AS	1220	67	3	2	9	1	0							
AS	1220	67	3	2	10	1	0							
AS	1220	67	3	2	11	1	0							
AS	1220	67	3	2	12	1	0							
AS	1220	67	3	2	13	1	0							
AS	1220	67	3	2	14	1	0							
AS	1220	67	3	2	15	1	0							
AS	1221	62	1	1	0	1	0							
AS	1221	62	1	1	1	1	0							
AS	1221	62	1	1	2	1	0							
AS	1221	62	1	1	3	1	0							
AS	1221	62	1	1	4	1	1	68,74	35,18	91,55	5x3,	0	1	0,532
AS	1221	62	1	1	5	1	0							
AS	1221	62	1	1	6	1	0							
AS	1221	62	1	1	7	1	1	69,64	28,39	92,2	9x12	1	0	0,683
AS	1222	56	1	2	0	1	1	64,45	54,99	85,7	2x3	0	1	0,311
AS	1222	56	1	2	0	1	1	66,02	55,22	86,03	2x1	0	1	0,358
AS	1222	56	1	2	1	1	0							
AS	1222	56	1	2	2	1	0							
AS	1222	56	1	2	3	1	0							

AS	1222	56	1	2	4	1	0							
AS	1222	56	1	2	5	1	0							
AS	1222	56	1	2	6	1	1	66,5	43,02	97,59	2x2	0	1	0,426
AS	1222	56	1	2	7	1	1	60,46	48,31	96,77	14x13	1	0	1,73
AS	1222	56	1	2	7	1	1	63,31	46,36	92,1	7x6	1	0	0,78
AS	1223	57	3	1	8	1	0							
AS	1223	57	3	1	9	1	1	52,23	52,19	44,55	5x6	0	0	1,894
AS	1223	57	3	1	10	1	0							
AS	1223	57	3	1	11	1	1	63,36	47,82	44,63	4x7	1	0	1,447
AS	1223	57	3	1	12	1	0							
AS	1223	57	3	1	13	1	0							
AS	1223	57	3	1	14	1	1	55,58	47	50,94	2x2	0	1	0,234
AS	1223	57	3	1	15	1	0							
AS	1224	56	0	1	0	1	0							
AS	1224	56	0	1	1	1	0							
AS	1224	56	0	1	2	1	0							
AS	1224	56	0	1	3	1	1	84,45	43,19	80,44	8x10	1	0	1,636
AS	1224	56	0	1	4	1								
AS	1224	56	0	1	5	1								
AS	1224	56	0	1	6	1								
AS	1224	56	0	1	7	1								
AS	1225	57	0	2	0	1	1	45,92	49,08	75,92	2x2	0	1	0,267
AS	1225	57	0	2	0	1	1	43,33	50,91	84,04	10x16	0	0	1,145
AS	1225	57	0	2	1	1	1	50,08	43,77	80,35	11x14	0	0	1,207
AS	1225	57	0	2	2	1	1	46,08	41	82,23	2x3	0	0	0,495
AS	1225	57	0	2	3	1	0							
AS	1225	57	0	2	4	1								
AS	1225	57	0	2	5	1								
AS	1225	57	0	2	6	1								
AS	1225	57	0	2	7	1								
AS	1226	67	2	1	8	1	1	75,8	51,82	33,4	1x2	0	1	0,268
AS	1226	67	2	1	8	1	1	81,76	51,46	3,07	4x6	0	0	0,87
AS	1226	67	2	1	8	1	1	79,5	51,7	34,55	3x3	0	1	0,298
AS	1226	67	2	1	9	1	0							
AS	1226	67	2	1	10	1	1	82,97	45,16	31,27	40x57	1	0	4,308
AS	1226	67	2	1	11	1	0							
AS	1226	67	2	1	12	1								
AS	1226	67	2	1	13	1								
AS	1226	67	2	1	14	1								
AS	1226	67	2	1	15	1								
AS	1227	63	2	2	8	1								
AS	1227	63	2	2	9	1								
AS	1227	63	2	2	10	1								
AS	1227	63	2	2	11	1								
AS	1227	63	2	2	12	1								
AS	1227	63	2	2	13	1								
AS	1227	63	2	2	14	1								
AS	1227	63	2	2	15	1								
AS	1230	31	1	1	0	1	1	61,74	58,36	92,01	3x6	0	1	0,436
AS	1230	31	1	1	1	1	0							
AS	1230	31	1	1	2	0								
AS	1230	31	1	1	3	1	0							
AS	1230	31	1	1	4	1	0							
AS	1230	31	1	1	5	1	0							
AS	1230	31	1	1	6	1	0							
AS	1230	31	1	1	7	1	0							
AS	1231	67	0	1	0	1	1	77,45	38,95	64,31	3x1	0	1	0,304
AS	1231	67	0	1	0	1	1	81,55	38,51	64,64	2x2	0	1	0,261
AS	1231	67	0	1	1	1	1	73,64	36,03	61,85	2x1	0	1	0,171
AS	1231	67	0	1	2	1	1	81,39	28,96	62,51	1x2	0	1	0,115
AS	1231	67	0	1	3	1	0							
AS	1231	67	0	1	4	1	1	81,75	35,24	75,46	4x4	0	0	0,444
AS	1231	67	0	1	4	1	1	82,93	34,97	71,04	3x2	0	1	0,312
AS	1231	67	0	1	5	1	1	74,91	31,12	74,81	4x4	0	1	0,345
AS	1231	67	0	1	6	1	1	76,27	24,92	73,5	7x4	0	0	0,593
AS	1231	67	0	1	7	1	0							
AS	1232	67	1	2	0	1	0							
AS	1232	67	1	2	1	1	0							
AS	1232	67	1	2	2	1	0							
AS	1232	67	1	2	3	1	0							
AS	1232	67	1	2	4	1	0							
AS	1232	67	1	2	5	1	0							
AS	1232	67	1	2	6	1	0							
AS	1232	67	1	2	7	1	0							
AS	1233	63	3	1	8	1	1	59,31	52,18	37,34	7x6	0	0	0,469
AS	1233	63	3	1	8	1	1	56,75	53,82	39,96	4x6	1	0	0,41
AS	1233	63	3	1	9	1	1	48,52	50,91	41,11	3x3	1	0	0,477
AS	1233	63	3	1	10	1	1	50,71	47,42	41,27	5x13	0	0	0,774
AS	1233	63	3	1	11	1	1	57,54	47,22	40,21	12x20	0	0	1,886
AS	1233	63	3	1	11	1	1	59,15	48,86	40,13	8x4	0	1	0,51
AS	1233	63	3	1	12	1	0							
AS	1233	63	3	1	13	1	0							
AS	1233	63	3	1	14	1	1	56,92	45,37	44,72	14x11	1	0	1,564
AS	1233	63	3	1	15	1	1	59,84	50,41	46,28	11x3	1	0	1,021
AS	1234	27	3	2	8	1	0							
AS	1234	27	3	2	9	1	0							
AS	1234	27	3	2	10	1	0							
AS	1234	27	3	2	11	1	0							
AS	1234	27	3	2	12	1	0							
AS	1234	27	3	2	13	1	0							
AS	1234	27	3	2	14	1	0							
AS	1234	27	3	2	15	1	0							
AS	1235	63	1	1	0	1								
AS	1235	63	1	1	1	1								
AS	1235	63	1	1	2	1								
AS	1235	63	1	1	3	1								
AS	1235	63	1	1	4	1								
AS	1235	63	1	1	5	1								
AS	1235	63	1	1	6	1								
AS	1235	63	1	1	7	1								
AS	1236	27	0	1	0	1	1	73,37	52,73	81,94	3x1	0	1	0,285
AS	1236	27	0	1	1	1	0							
AS	1236	27	0	1	2	1	0							
AS	1236	27	0	1	3	1	0							
AS	1236	27	0	1	4	1	1	75,27	51,6	92,43	5x2	0	1	0,71
AS	1236	27	0	1	5	1	0							
AS	1236	27	0	1	6	1	1	73,2	41,24	91,61	2x1	0	1	0,26
AS	1236	27	0	1	7	1	0							
AS	1237	63	0	2	0	1	0							
AS	1237	63	0	2	1	1	0							
AS	1237	63	0	2	2	1	0							
AS	1237	63	0	2	3	1	1	38,79	41,65	68,08	2x5	0	0	0,905
AS	1237	63	0	2	4	1	0							
AS	1237	63	0	2	5	1	0							
AS	1237	63	0	2	6	1	0							
AS	1237	63	0	2	7	1	1	41,75	40,36	82,68	3x1	0	1	0,492
AS	1237	63	0	2	7	1	1	43,97	38,03	75,96	4x3	0	0	0,576
AS	1238	42	2	1	8	1	0							
AS	1238	42	2	1	9	1	0							
AS	1238	42	2	1	10	1	0							
AS	1238	42	2	1	11	1	0							
AS	1238	42	2	1	12	1	0							
AS	1238	42	2	1	13	1	0							
AS	1238	42	2	1	14	1	0							
AS	1238	42	2	1	15	1	0							
AS	1239	4	2	2	8	1	0							
AS	1239	4	2	2	9	1	0							
AS	1239	4	2	2	10	1	0							
AS	1239	4	2	2	11	1	1	34,86	55,94	31,59	2x2	0	1	0,157
AS	1239	4	2	2	12	1	0							
AS	1239	4	2	2	13	1	1	45,42	52,51	38,89	7x13	0	0	3,171
AS	1239	4	2	2	14	1	0							
AS	1239	4	2	2	15	1	1	36,17	55,15	39,54	11x3	0	0	0,711
AS	1240	27	1	2	0	1	1	68,35	53,08	82,19	2x3	0	1	0,437
AS	1240	27	1	2	1	1	1	70,66	46,62	80,64	2x1	0	1	0,406
AS	1240	27	1	2	2	1	0							
AS	1240	27	1	2	3	1	0							
AS	1240	27	1	2	4	1	0							
AS	1240	27	1	2	5	1	0							
AS	1240	27	1	2	6	1	0							
AS	1240	27	1	2	7	1	1	62,14	47,67	95,31	10x9	0	0	1,265
AS	1241	4	1	1	0	1	1	56,37	50,33	71,19	3x3	0	1	0,362
AS	1241	4	1	1	0	1	1	57,4	50,28	73,56	16x19	1	0	2,428
AS	1241	4	1	1	1	1	1	52,04	46,2	73,97	17x27	0	0	0,884
AS	1241	4	1	1	2	1	0							
AS	1241	4	1	1	3	1	1	62,15	41,46	72,42	7x8	0	1	0,774
AS	1241	4	1	1	3	1	1	63,01	43,91	73,15	4x8	0	0	0,546
AS	1241	4	1	1	4	1								
AS	1241	4	1	1	5	1								
AS	1241	4	1	1	6	1								
AS	1241	4	1	1	7	1								
AS	1242	2	1	1	0	1	1	52,71	45,56	72,22	1x3	0	1	0,508
AS	1242	2	1	1	1	1	0							
AS	1242	2	1	1	2	1	0							
AS	1242	2	1	1	3	1	1	61	35,89	73,53	2x21	1	0	3,214
AS	1242	2	1	1	4	1	0							
AS	1242	2	1	1	5	1	0							
AS	1242	2	1	1	6	1	0							
AS	1242	2	1	1	7	1	1	59,84	35,25	81,81	2x6	0	0	0,476
AS	1243	2	1	2	0	1	0							
AS	1243	2	1	2	1	1	0							
AS	1243	2	1	2	2	1	0							
AS	1243	2	1	2	3	1	0							
AS	1243	2	1	2	4	1	1	44,12	48,59	87,35	4x2	0	1	0,509
AS	1243	2	1	2	5	1	0							

AS	1243	2	1	2	6	1	0							
AS	1243	2	1	2	7	1	1	38,1	45,24	88,99	3x4	0	1	0,446
AS	1244	4	0	2	0	1	0							
AS	1244	4	0	2	1	1	1	50,3	47,57	68,69	2x2	0	1	0,323
AS	1244	4	0	2	2	1	0							
AS	1244	4	0	2	3	1	1	37,35	47,21	69,68	14x18	0	0	1,266
AS	1244	4	0	2	4	1	0							
AS	1244	4	0	2	5	1	0							
AS	1244	4	0	2	6	1	1	45,32	39,85	79,19	2x2	0	1	0,339
AS	1244	4	0	2	7	1	0							
AS	1245	2	3	1	8	1	0							
AS	1245	2	3	1	9	1	1	48,24	53,85	31,02	2x1	0	1	0,271
AS	1245	2	3	1	10	1	0							
AS	1245	2	3	1	11	1	0							
AS	1245	2	3	1	12	1	0							
AS	1245	2	3	1	13	1	1	49,7	54,64	37,17	7x9	0	0	0,643
AS	1245	2	3	1	14	1	0							
AS	1245	2	3	1	15	1	0							
AS	1246	4	3	1	8	1	0							
AS	1246	4	3	1	9	1	0							
AS	1246	4	3	1	10	1	0							
AS	1246	4	3	1	11	1	1	60,61	53,5	29,79	2x1	0	1	0,204
AS	1246	4	3	1	12	1	0							
AS	1246	4	3	1	13	1	1	51,71	56,86	36,92	11x6	1	0	0,781
AS	1246	4	3	1	14	1	0							
AS	1246	4	3	1	15	1	0							
AS	1247	2	2	2	8	1	1	42,42	58,15	38,12	2x2	0	1	0,364
AS	1247	2	2	2	8	1	1	39,17	58,04	37,87	2x2	0	1	0,168
AS	1247	2	2	2	9	1	0							
AS	1247	2	2	2	10	1	0							
AS	1247	2	2	2	11	1	0							
AS	1247	2	2	2	12	1	0							
AS	1247	2	2	2	13	1	0							
AS	1247	2	2	2	14	1	0							
AS	1247	2	2	2	15	1	0							
AS	1248	13	1	1	0	1	1	62,76	50,25	76,74	2x1	0	1	0,504
AS	1248	13	1	1	1	1	0							
AS	1248	13	1	1	2	1	0							
AS	1248	13	1	1	3	1	0							
AS	1248	13	1	1	4	1	0							
AS	1248	13	1	1	5	1	1	56,52	45,98	87,65	8x5	0	1	0,545
AS	1248	13	1	1	6	1	1	58,97	39,01	87,89	6x4	0	1	0,534
AS	1248	13	1	1	7	1	0							
AS	1249	18	3	1	8	1	0							
AS	1249	18	3	1	9	1	0							
AS	1249	18	3	1	10	1	0							
AS	1249	18	3	1	11	1	0							
AS	1249	18	3	1	12	1	0							
AS	1249	18	3	1	13	1	0							
AS	1249	18	3	1	14	1	0							
AS	1249	18	3	1	15	1	1	65,19	50,96	45,67	18x12	0	0	1,03
AS	1250	13	2	1	8	1	1	83,86	53,46	48,69	6x5	0	1	0,634
AS	1250	13	2	1	9	1	0							
AS	1250	13	2	1	10	1	0							
AS	1250	13	2	1	11	1	0							
AS	1250	13	2	1	12	1	0							
AS	1250	13	2	1	13	1	0							
AS	1250	13	2	1	14	1	0							
AS	1250	13	2	1	15	1	0							
AS	1251	18	0	1	0	1	0							
AS	1251	18	0	1	1	1	1	74,14	41,77	73,6	5x1	0	1	0,19
AS	1251	18	0	1	2	1	0							
AS	1251	18	0	1	3	1	0							
AS	1251	18	0	1	4	1	1	82,74	44,61	85,9	3x3	0	1	0,39
AS	1251	18	0	1	5	1	0							
AS	1251	18	0	1	6	1	0							
AS	1251	18	0	1	7	1	1	83,04	39,97	89,59	1x4	0	1	0,544
AS	1252	56	2	1	8	1	1	78,57	57,24	39,46	3x3	0	1	0,455
AS	1252	56	2	1	8	1	1	76,13	58,18	40,37	3x3	0	1	0,298
AS	1252	56	2	1	9	1	0							
AS	1252	56	2	1	10	1	0							
AS	1252	56	2	1	11	1	1	83,57	53,82	37,41	2X2	0	1	0,345
AS	1252	56	2	1	12	1	0							
AS	1252	56	2	1	13	1	0							
AS	1252	56	2	1	14	1	0							
AS	1252	56	2	1	15	1	0							
AS	1253	45	3	1	0	1	1	58,19	48,8	95,81	2x2	0	1	0,337
AS	1253	45	3	1	1	1	0							
AS	1253	45	3	1	2	1	0							
AS	1253	45	3	1	3	1	0							
AS	1253	45	3	1	4	1	0							
AS	1253	45	3	1	5	1	0							
AS	1253	45	3	1	6	1	1	55,69	36,25	106,23	3x2	0	1	0,516
AS	1253	45	3	1	7	1	0							
AS	1254	57	1	1	0	1	1	57,17	52,31	85,9	3x1	0	1	0,165
AS	1254	57	1	1	0	1	1	62,88	51,03	87,05	3x3	0	1	0,548
AS	1254	57	1	1	0	1	1	59,15	52,95	89,1	1x2	0	1	0,782
AS	1254	57	1	1	1	1	0							
AS	1254	57	1	1	2	1	1	58,48	41,62	90,66	3x4	0	1	0,394
AS	1254	57	1	1	2	1	1	61,41	41,4	90,99	10x38	1	0	1,149
AS	1254	57	1	1	3	1	0							
AS	1254	57	1	1	4	1	1	62,69	49,75	101,32	8x8	1	1	0,878
AS	1254	57	1	1	5	1	1	53,61	48,01	100,42	1x2	0	1	0,186
AS	1254	57	1	1	6	1	0							
AS	1254	57	1	1	7	1	1	64,9	45,36	101,16	14x8	1	0	1,278
AS	1254	57	1	1	7	1	1	62,13	43,84	98,45	36x29	1	0	7,155
AS	1255	33	3	2	8	1	0							
AS	1255	33	3	2	9	1	0							
AS	1255	33	3	2	10	1	0							
AS	1255	33	3	2	11	1	0							
AS	1255	33	3	2	12	0								
AS	1255	33	3	2	13	0								
AS	1255	33	3	2	14	0								
AS	1255	33	3	2	15	0								
AS	1256	45	2	1	8	1	1	76,54	53,41	54,13	2x2	0	1	0,266
AS	1256	45	2	1	8	1	1	77,2	53,49	55,12	2x1	0	1	0,247
AS	1256	45	2	1	8	1	1	80,68	51,52	55,77	2x2	0	1	0,244
AS	1256	45	2	1	9	1	0							
AS	1256	45	2	1	10	1	1	80,62	44,49	52,99	3x2	0	1	0,229
AS	1256	45	2	1	11	1	0							
AS	1256	45	2	1	12	1	0							
AS	1256	45	2	1	13	1	0							
AS	1256	45	2	1	14	1	0							
AS	1256	45	2	1	15	1	0							
AS	1257	44	0	1	0	1	0							
AS	1257	44	0	1	1	1	0							
AS	1257	44	0	1	2	1	0							
AS	1257	44	0	1	3	1	0							
AS	1257	44	0	1	4	1	0							
AS	1257	44	0	1	5	1	1	78,43	37,59	73,9	2x2	0	1	0,222
AS	1257	44	0	1	6	1	0							
AS	1257	44	0	1	7	1	0							
AS	1258	42	1	1	0	1	1	58,68	55,64	93,68	3x1	0	1	0,324
AS	1258	42	1	1	1	1	0							
AS	1258	42	1	1	2	1	0							
AS	1258	42	1	1	3	1	0							
AS	1258	42	1	1	4	1	0							
AS	1258	42	1	1	5	1	0							
AS	1258	42	1	1	6	1	1	56,98	43,15	105,25	2x2	0	0	0,738
AS	1258	42	1	1	7	1	0							
AS	1259	23	1	1	0	1	1	58,64	49,52	87,07	6x10	0	1	0,645
AS	1259	23	1	1	1	1	0							
AS	1259	23	1	1	2	1	0							
AS	1259	23	1	1	3	1	1	63,89	45,17	84,36	2x2	0	1	0,329
AS	1259	23	1	1	4	1	0							
AS	1259	23	1	1	5	1	0							
AS	1259	23	1	1	6	1	0							
AS	1259	23	1	1	7	1	0							
AS	1260	44	2	1	8	1	1	66,83	55,02	26,39	4x5	0	0	0,648
AS	1260	44	2	1	9	1	0							
AS	1260	44	2	1	10	1	0							
AS	1260	44	2	1	11	1	0							
AS	1260	44	2	1	12	1	0							
AS	1260	44	2	1	13	1	0							
AS	1260	44	2	1	14	1	0							
AS	1260	44	2	1	15	1	0							
AS	1261	37	1	1	0	1	1	57,83	53,95	85,54	2x2	0	1	0,246
AS	1261	37	1	1	0	1	1	59,17	53,73	85,46	2x1	0	1	0,394
AS	1261	37	1	1	0	1	1	54,09	54,96	85,79	2x2	0	1	0,363
AS	1261	37	1	1	1	1	0							
AS	1261	37	1	1	2	1	1	58,78	43,46	87,84	3x4	0	1	0,407
AS	1261	37	1	1	3	1	0							
AS	1261	37	1	1	4	1	1	60,25	53,68	99,97	2x14	0	0	0,602
AS	1261	37	1	1	5	1	0							
AS	1261	37	1	1	6	1	1	53,94	43,3	99,24	5x6	0	1	0,675
AS	1261	37	1	1	7	1	0							
AS	1262	27	2	1	8	1	1	74,26	53,91	41,7	2x1	0	1	0,245
AS	1262	27	2	1	9	1	0							
AS	1262	27	2	1	10	1	1	70,06	50,28	39,65	3x1	0	1	0,155
AS	1262	27	2	1	11	1	1	75,05	50,91	41,29	3x2	0	1	0,247
AS	1262	27	2	1	12	1	0							
AS	1262	27	2	1	13	1	0							
AS	1262	27	2	1	14	1	0							
AS	1262	27	2	1	15	1	0							
AS	1263	41	3	1	8	1	1	61,79	57,38	48,79	2x2	0	1	0,238

AS	1263	41	3	1	8	1	1	56,43	59,05	49,85	3x3	0	1	0,262
AS	1263	41	3	1	9	1	0							
AS	1263	41	3	1	10	1	0							
AS	1263	41	3	1	11	1	0							
AS	1263	41	3	1	12	1	0							
AS	1263	41	3	1	13	1	0							
AS	1263	41	3	1	14	1	0							
AS	1263	41	3	1	15	1	0							
AS	1264	36	0	1	0	1	1	77,49	45,72	79,89	3x4	0	1	0,565
AS	1264	36	0	1	0	1	1	81,44	45,88	80,22	2x5	0	1	0,561
AS	1264	36	0	1	1	1	0							
AS	1264	36	0	1	2	1	0							
AS	1264	36	0	1	3	1	0							
AS	1264	36	0	1	4	1	1	79,99	43,79	91,04	6x6	0	0	1,113
AS	1264	36	0	1	5	1	0							
AS	1264	36	0	1	6	1	1	80,58	32,26	89,73	2x4	0	1	0,582
AS	1264	36	0	1	7	1	0							
AS	1265	37	2	1	8	1	0							
AS	1265	37	2	1	9	1	0							
AS	1265	37	2	1	10	1	0							
AS	1265	37	2	1	11	1	0							
AS	1265	37	2	1	12	1	0							
AS	1265	37	2	1	13	1	0							
AS	1265	37	2	1	14	1	0							
AS	1265	37	2	1	15	1	0							
AS	1266	33	0	2	0	1	0							
AS	1266	33	0	2	1	1	0							
AS	1266	33	0	2	2	1	0							
AS	1266	33	0	2	3	1	0							
AS	1266	33	0	2	4	0								
AS	1266	33	0	2	5	0								
AS	1266	33	0	2	6	0								
AS	1266	33	0	2	7	0								
AS	1267	36	3	1	8	1	1	62,11	55,06	46,44	4x4	0	1	0,452
AS	1267	36	3	1	9	1	0							
AS	1267	36	3	1	10	1	0							
AS	1267	36	3	1	11	1	0							
AS	1267	36	3	1	12	1	0							
AS	1267	36	3	1	13	1	0							
AS	1267	36	3	1	14	1	0							
AS	1267	36	3	1	15	1	0							
AS	1268	66	0	1	0	1	1	82,45	49,89	87,71	7x5	0	1	0,554
AS	1268	66	0	1	0	1	1	78,77	49,25	88,78	7x8	0	1	0,688
AS	1268	66	0	1	1	1	0							
AS	1268	66	0	1	2	1	0							
AS	1268	66	0	1	3	1	0							
AS	1268	66	0	1	4	1	0							
AS	1268	66	0	1	5	1	1	74,49	42,46	97,31	4x3	0	1	2,293
AS	1268	66	0	1	6	1	0							
AS	1268	66	0	1	7	1	0							
AS	1269	19	0	2	0	1	1	46,03	49,32	69,25	2x3	0	1	0,34
AS	1269	19	0	2	1	1	0							
AS	1269	19	0	2	2	1	0							
AS	1269	19	0	2	3	1	0							
AS	1269	19	0	2	4	0								
AS	1269	19	0	2	5	0								
AS	1269	19	0	2	6	0								
AS	1269	19	0	2	7	0								
AS	1270	21	2	1	8	1	1	77,97	53,48	41,2	4x4	0	1	0,397
AS	1270	21	2	1	9	1	0							
AS	1270	21	2	1	10	1	0							
AS	1270	21	2	1	11	1	0							
AS	1270	21	2	1	12	1	0							
AS	1270	21	2	1	13	1	0							
AS	1270	21	2	1	14	1	0							
AS	1270	21	2	1	15	1	0							
AS	1271	23	2	2	8	1	1	45,13	56,37	32,7	3x2	0	1	0,332
AS	1271	23	2	2	8	1	1	47,73	57,09	34,01	3x1	0	1	0,611
AS	1271	23	2	2	9	1	1	55	53,61	33,19	2x1	0	1	0,146
AS	1271	23	2	2	10	1	0							
AS	1271	23	2	2	11	1	0							
AS	1271	23	2	2	12	1	0							
AS	1271	23	2	2	13	1	0							
AS	1271	23	2	2	14	1	0							
AS	1271	23	2	2	15	1	0							
AS	1272	66	1	2	0	1	0							
AS	1272	66	1	2	1	1	0							
AS	1272	66	1	2	2	1	1	62,41	42,02	89,1	7x5	0	1	0,604
AS	1272	66	1	2	3	1	1	58,4	46,92	90,49	19x20	0	0	1,485
AS	1272	66	1	2	4	1	0							
AS	1272	66	1	2	5	1	1	69,76	46,88	99,02	4x4	0	1	0,382
AS	1272	66	1	2	6	1	0							
AS	1272	66	1	2	7	1	1	60,78	44,54	97,71	21x17	1	0	1,531
AS	1273	19	3	1	8	1	0							
AS	1273	19	3	1	9	1	0							
AS	1273	19	3	1	10	1	0							
AS	1273	19	3	1	11	1	1	66,87	47,35	50,63	13x6	1	0	0,71
AS	1273	19	3	1	12	1	0							
AS	1273	19	3	1	13	1	0							
AS	1273	19	3	1	14	1	0							
AS	1273	19	3	1	15	1	0							
AS	1274	21	3	2	8	1	0							
AS	1274	21	3	2	9	1	0							
AS	1274	21	3	2	10	1	1	62,71	46,16	51,94	2x2	0	1	0,39
AS	1274	21	3	2	11	1	0							
AS	1274	21	3	2	12	1	1	60,2	51,11	60,87	1x2	0	1	0,367
AS	1274	21	3	2	13	1	0							
AS	1274	21	3	2	14	1	0							
AS	1274	21	3	2	15	1	0							
AS	1275	66	2	1	8	1	0							
AS	1275	66	2	1	9	1	0							
AS	1275	66	2	1	10	1	1	80,52	52,72	45,82	2x2	0	1	0,245
AS	1275	66	2	1	11	1	1	82,82	54,85	44,36	2x2	0	1	0,319
AS	1275	66	2	1	12	1	0							
AS	1275	66	2	1	13	1	0							
AS	1275	66	2	1	14	1	0							
AS	1275	66	2	1	15	1	0							
AS	1276	19	2	2	8	1	0							
AS	1276	19	2	2	9	1	0							
AS	1276	19	2	2	10	1	0							
AS	1276	19	2	2	11	1	0							
AS	1276	19	2	2	12	1	0							
AS	1276	19	2	2	13	1	1	50,08	53,22	34,74	7x7	0	0	0,807
AS	1276	19	2	2	14	1	0							
AS	1276	19	2	2	15	1	0							
AS	1277	66	3	2	8	1	1	61,32	53,42	40,83	4x2	0	1	0,323
AS	1277	66	3	2	9	1	1	71,19	51,9	44,28	1x2	0	1	0,125
AS	1277	66	3	2	10	1	0							
AS	1277	66	3	2	11	1	0							
AS	1277	66	3	2	12	1	0							
AS	1277	66	3	2	13	1	0							
AS	1277	66	3	2	14	1	0							
AS	1277	66	3	2	15	1	0							
AS	1278	60	0	1	0	1	1	73,89	45,39	78,54	4x2	0	1	0,502
AS	1278	60	0	1	1	1	1	71,25	39,07	81,41	5x19	0	0	2,136
AS	1278	60	0	1	2	1	0							
AS	1278	60	0	1	3	1	1	84,67	39,07	81,89	17x40	1	0	3,305
AS	1278	60	0	1	4	1	0							
AS	1278	60	0	1	5	1	1	70,83	34,49	93,62	2x2	0	0	0,468
AS	1278	60	0	1	5	1	1	71,77	35	87,47	3x4	0	0	0,543
AS	1278	60	0	1	6	1	1	75,52	32,67	90,92	5x19	0	1	0,287
AS	1278	60	0	1	6	1	1	76,21	32,15	88,46	18x22	0	0	1,385
AS	1278	60	0	1	7	1	1	81,97	38,17	87,39	8x8	0	0	1,673
AS	1278	60	0	1	7	1	1	80,28	37,25	85,34	4x4	0	0	0,789
AS	1279	60	1	2	0	1	0							
AS	1279	60	1	2	1	1	0							
AS	1279	60	1	2	2	1	0							
AS	1279	60	1	2	3	1	1	60,11	44,28	84,75	2x1	0	1	0,336
AS	1279	60	1	2	4	1	0							
AS	1279	60	1	2	5	1	1	70,52	43,19	95,5	5x7	0	0	0,824
AS	1279	60	1	2	6	1	0							
AS	1279	60	1	2	7	1	1	61,46	39,15	93,69	3x4	0	0	0,878
AS	1280	60	2	1	8	1	0							
AS	1280	60	2	1	9	1	1	74,05	53,4	47,8	14x30	1	0	1,276
AS	1280	60	2	1	10	1	0							
AS	1280	60	2	1	11	1	1	81,38	51,16	46,74	27x30	1	0	2,844
AS	1280	60	2	1	12	0								
AS	1280	60	2	1	13	0								
AS	1280	60	2	1	14	0								
AS	1280	60	2	1	15	0								
AS	1281	19	1	1	0	1	1	61,7	47,98	93,48	3x5	0	1	0,432
AS	1281	19	1	1	0	1	1	59,76	48,22	93,48	2x1	0	1	0,435
AS	1281	19	1	1	0	1	1	59,28	47,97	95,86	10x8	0	0	1,871
AS	1281	19	1	1	0	1	1	64,47	47,07	89,79	2x4	0	1	0,289
AS	1281	19	1	1	1	1	0							
AS	1281	19	1	1	2	1	0							
AS	1281	19	1	1	3	1	0							
AS	1281	19	1	1	4	1	0							
AS	1281	19	1	1	5	1	1	52,45	40,83	402,34	1x4	0	1	0,455
AS	1281	19	1	1	6	1	0							
AS	1281	19	1	1	7	1	1	60,59	35,08	104,72	19x12	1	0	1,216
AS	1282	21	1	2	0	1	1	58,03	51,97	94,22	3x4	0	1	0,572
AS	1282	21	1	2	1	1	0							
AS	1282	21	1	2	2	1	1	59,83	41,7	91,26	2x1	0	1	0,291
AS	1282	21	1	2	3	1	0							

AS	1282	21	1	2	4	1	1	62,44	52,18	106,68	2x1	0	1	0,323
AS	1282	21	1	2	4	1	1	59,77	51,32	105,11	2x2	0	1	0,362
AS	1282	21	1	2	5	1	1	67,28	48,37	111,27	2x1	0	1	0,361
AS	1282	21	1	2	5	1	1	66,9	46,5	104,3	5x4	0	1	0,21
AS	1282	21	1	2	6	1	0							
AS	1282	21	1	2	7	1	1	57,19	45,88	105,61	8x5	1	0	0,572
AS	1283	53	1	2	0	0								
AS	1283	53	1	2	1	0								
AS	1283	53	1	2	2	0								
AS	1283	53	1	2	3	0								
AS	1283	53	1	2	4	0								
AS	1283	53	1	2	5	0								
AS	1283	53	1	2	6	0								
AS	1283	53	1	2	7	0								
AS	1284	54	3	2	8	1	0							
AS	1284	54	3	2	9	1	0							
AS	1284	54	3	2	10	1	0							
AS	1284	54	3	2	11	1	0							
AS	1284	54	3	2	12	1	0							
AS	1284	54	3	2	13	1	1	68,09	55,68	60,22	3x3	0	1	0,386
AS	1284	54	3	2	13	1	1	68,25	56,49	58,33	28x21	1	0	2,278
AS	1284	54	3	2	14	1	1	64,34	47,34	57,27	1x2	0	1	0,219
AS	1284	54	3	2	15	1	0							
AS	1285	21	0	1	0	1	1	80,62	45,14	78,36	2x1	0	1	0,349
AS	1285	21	0	1	1	1	0							
AS	1285	21	0	1	2	1	0							
AS	1285	21	0	1	3	1	0							
AS	1285	21	0	1	4	1	1	80,46	42,31	89,84	5x5	1	0	0,321
AS	1285	21	0	1	5	1	0							
AS	1285	21	0	1	6	1	0							
AS	1285	21	0	1	7	1	1	83,18	37	90,57	12x17	1	0	1,163
AS	1286	53	3	2	8	1	0							
AS	1286	53	3	2	9	1	0							
AS	1286	53	3	2	10	1	0							
AS	1286	53	3	2	11	1	0							
AS	1286	53	3	2	12	1	0							
AS	1286	53	3	2	13	1	0							
AS	1286	53	3	2	14	1	0							
AS	1286	53	3	2	15	1	0							
AS	1287	55	1	1	0	1	0							
AS	1287	55	1	1	1	1	0							
AS	1287	55	1	1	2	1	0							
AS	1287	55	1	1	3	1	0							
AS	1287	55	1	1	4	1	0							
AS	1287	55	1	1	5	1	0							
AS	1287	55	1	1	6	1	0							
AS	1287	55	1	1	7	1	1	65,04	41,39	83,32	11x9	1	0	1,057
AS	1288	41	0	1	0	1	1	78,77	52,6	88,08	2x4	0	1	0,497
AS	1288	41	0	1	1	1	1	71,16	50,15	88	2x2	0	1	0,225
AS	1288	41	0	1	2	1	0							
AS	1288	41	0	1	3	1	0							
AS	1288	41	0	1	4	1	0							
AS	1288	41	0	1	5	1	0							
AS	1288	41	0	1	6	1	0							
AS	1288	41	0	1	7	1	0							
AS	1289	23	0	2	0	1	1	46,41	51,78	71,37	3x2	0	1	0,506
AS	1289	23	0	2	1	1	0							
AS	1289	23	0	2	2	1	0							
AS	1289	23	0	2	3	1	1	43,67	51,5	68,99	3x2	0	1	0,468
AS	1289	23	0	2	4	1	0							
AS	1289	23	0	2	5	1	0							
AS	1289	23	0	2	6	1	0							
AS	1289	23	0	2	7	1	1	42,69	45,69	83,91	2x3	0	1	0,317
AS	1290	54	2	1	8	1	0							
AS	1290	54	2	1	9	1	0							
AS	1290	54	2	1	10	1	0							
AS	1290	54	2	1	11	1	0							
AS	1290	54	2	1	12	1	0							
AS	1290	54	2	1	13	1	0							
AS	1290	54	2	1	14	1	0							
AS	1290	54	2	1	15	1	1	89,87	43,52	46,52	23x17	1	0	1,797
AS	1291	54	0	1	0	1	0							
AS	1291	54	0	1	1	1	0							
AS	1291	54	0	1	2	1	1	85,51	37,32	83	10x10	0	0	1,963
AS	1291	54	0	1	3	1	1	88,33	40,25	81,2	26x32	0	0	3,23
AS	1291	54	0	1	4	1	1	83,03	44,32	92,43	6x6	0	1	0,623
AS	1291	54	0	1	5	1	1	75,68	39,12	90,54	2x3	0	1	0,288
AS	1291	54	0	1	6	1	1	78,62	32,65	90,54	10x11	1	0	0,856
AS	1291	54	0	1	6	1	1	76,38	34,51	85,54	6x12	0	0	0,961
AS	1291	54	0	1	7	1	1	86,24	37,85	90,71	93x47	1	0	3,111
AS	1292	55	2	2	8	1	0							
AS	1292	55	2	2	9	1	0							
AS	1292	55	2	2	10	1	0							
AS	1292	55	2	2	11	1	0							
AS	1292	55	2	2	12	1	0							
AS	1292	55	2	2	13	1	0							
AS	1292	55	2	2	14	1	0							
AS	1292	55	2	2	15	1	1	29,37	53,15	43,54	24x32	1	0	3,085
AS	1293	54	1	2	0	1	0							
AS	1293	54	1	2	1	1	0							
AS	1293	54	1	2	2	1	1	58,91	44,62	87,22	13x8	1	0	1,027
AS	1293	54	1	2	3	1	0							
AS	1293	54	1	2	4	1	1	60,62	49,89	95,91	4x4	0	1	0,611
AS	1293	54	1	2	5	1	1	68,3	47,26	95,99	2x1	0	1	0,345
AS	1293	54	1	2	5	1	1	70,51	43,12	90,99	11x18	1	0	1,903
AS	1293	54	1	2	6	1	1	64,64	40,42	90,99	19x7	1	0	2,185
AS	1293	54	1	2	7	1	1	60,15	43,14	94,93	17x11	1	0	5,501
AS	1294	39	1	1	0	1	0							
AS	1294	39	1	1	1	1	0							
AS	1294	39	1	1	2	0								
AS	1294	39	1	1	3	1	0							
AS	1294	39	1	1	4	1	0							
AS	1294	39	1	1	5	1	0							
AS	1294	39	1	1	6	1	0							
AS	1294	39	1	1	7	1	0							
AS	1295	55	0	2	0	1	0							
AS	1295	55	0	2	1	1	0							
AS	1295	55	0	2	2	1	0							
AS	1295	55	0	2	3	1	0							
AS	1295	55	0	2	4	1	0							
AS	1295	55	0	2	5	1	0							
AS	1295	55	0	2	6	1	0							
AS	1295	55	0	2	7	1	0							
AS	1296	39	3	1	8	1	0							
AS	1296	39	3	1	9	1	1	55,03	51,56	38,32	2x2	0	1	0,154
AS	1296	39	3	1	10	1	0							
AS	1296	39	3	1	11	1	0							
AS	1296	39	3	1	12	1	0							
AS	1296	39	3	1	13	1	0							
AS	1296	39	3	1	14	1	0							
AS	1296	39	3	1	15	1	0							
AS	1297	55	3	1	8	1	0							
AS	1297	55	3	1	9	1	0							
AS	1297	55	3	1	10	1								
AS	1297	55	3	1	11	1	1	64,59	52,84	26,63	2x2	0	1	0,412
AS	1297	55	3	1	12	1	0							
AS	1297	55	3	1	13	1	0							
AS	1297	55	3	1	14	1	0							
AS	1297	55	3	1	15	1	0							
AS	1298	39	2	2	8	1	0							
AS	1298	39	2	2	9	1	0							
AS	1298	39	2	2	10	1	0							
AS	1298	39	2	2	11	1	0							
AS	1298	39	2	2	12	0								
AS	1298	39	2	2	13	0								
AS	1298	39	2	2	14	0								
AS	1298	39	2	2	15	0								
AS	1299	39	0	2	0	1	1	45,02	41,64	72,35	6x4	0	1	0,477
AS	1299	39	0	2	1	1	0							
AS	1299	39	0	2	2	1	0							
AS	1299	39	0	2	3	1	0							
AS	1299	39	0	2	4	0								
AS	1299	39	0	2	5	0								
AS	1299	39	0	2	6	0								
AS	1299	39	0	2	7	0								
AS	1300	23	3	1	8	1	1	57,54	51,88	42,03	6x5	0	1	0,494
AS	1300	23	3	1	8	1	1	54,59	53,34	43,01	4x2	0	1	0,647
AS	1300	23	3	1	9	1	1	49,57	53,22	40,06	4x3	0	1	0,209
AS	1300	23	3	1	10	1	0							
AS	1300	23	3	1	11	1	0							
AS	1300	23	3	1	12	1	0							
AS	1300	23	3	1	13	1	0							
AS	1300	23	3	1	14	1	0							
AS	1300	23	3	1	15	1	0							
AS	1301	53	2	1	8	1	0							
AS	1301	53	2	1	9	1	0							
AS	1301	53	2	1	10	1	1	77,86	50,82	41,89	5x3	0	0	0,538
AS	1301	53	2	1	11	1	0							
AS	1301	53	2	1	12	1	0							
AS	1301	53	2	1	13	1	0							
AS	1301	53	2	1	14	1	0							
AS	1301	53	2	1	15	1	0							
AS	1302	53	0	1	0	1	1	79,88	50,78	76,86	4x4	0	1	0,334
AS	1302	53	0	1	1	1	0							
AS	1302	53	0	1	2	1	0							

AS	1302	53	0	1	3	1	0							
AS	1302	53	0	1	4	1	0							
AS	1302	53	0	1	5	1	0							
AS	1302	53	0	1	6	1	1	77,32	37,8	87,51	3x2	0	1	0,308
AS	1302	53	0	1	7	1	0							
AS	1303	60	3	2	8	1	0							
AS	1303	60	3	2	9	1	0							
AS	1303	60	3		10	1	1	70,08	42,58	42,92	9x16	1	0	1,668
AS	1303	60	3	2	11	1	1	65,8	46,18	41,2	10x10	0	0	1,21
AS	1303	60	3	2	11	1	1	66,47	47,32	42,59	3x7	0	0	0,781
AS	1303	60	3	2	12	0								
AS	1303	60	3	2	13	0								
AS	1303	60	3	2	14	0								
AS	1303	60	3	2	15	0								
AS	1702	13	0	1	0	1	1	77,5	50,84	72,56	2x1	0	1	0,298
AS	1702	13	0	1	0	1	1	82,53	51,78	73,46	5x2	0	1	0,396
AS	1702	13	0	1	1	1	0							
AS	1702	13	0	1	2	1	1	86,19	43,92	76,99	15x12	1	0	1,453
AS	1702	13	0	1	3	1	1	88,49	45,24	75,1	4x7	0	0	0,538
AS	1702	13	0	1	4	1	1	80,85	52,11	85,93	2x8	0	0	0,697
AS	1702	13	0	1	5	1	0							
AS	1702	13	0	1	6	1	1	79,26	40,22	83,22	4x4	0	1	0,229
AS	1702	13	0	1	7	1	0							
AS	1705	13	3	1	8	1	1	62,25	50,55	47,38	8x9	1	0	0,486
AS	1705	13	3	1	8	1	1	59,8	51,1	47,62	7x5	1	0	0,292
AS	1705	13	3	1	9	1	0							
AS	1705	13	3	1	10	1	0							
AS	1705	13	3	1	11	1	0							
AS	1705	13	3	1	12	1	0							
AS	1705	13	3	1	13	1	0							
AS	1705	13	3	1	14	1	0							
AS	1705	13	3	1	15	1	0							
AS	1708	18	1	1	0	1	1	61,05	48,86	81,96	5x7	0	1	0,353
AS	1708	18	1	1	1	1	0							
AS	1708	18	1	1	2	1	0							
AS	1708	18	1	1	3	1	0							
AS	1708	18	1	1	4	1	1	61,29	47,22	92,21	2x6	0	0	0,732
AS	1708	18	1	1	5	1	0							
AS	1708	18	1	1	6	1	0							
AS	1708	18	1	1	7	1	0							
AS	1711	18	2	1	8	1	1	82,56	54,53	43,37	1x3	0	1	0,282
AS	1711	18	2	1	9	1	0							
AS	1711	18	2	1	10	1	0							
AS	1711	18	2	1	11	1	0							
AS	1711	18	2	1	12	1	0							
AS	1711	18	2	1	13	1	0							
AS	1711	18	2	1	14	1	0							
AS	1711	18	2	1	15	1	0							
AS	1714	31	0	1	0	0								
AS	1714	31	0	1	1	0								
AS	1714	31	0	1	2	0								
AS	1714	31	0	1	3	0								
AS	1714	31	0	1	4	1	0							
AS	1714	31	0	1	5	1	1	74,39	44,14	98,16	2x2	0	1	0,313
AS	1714	31	0	1	6	1	0							
AS	1714	31	0	1	7	1	0							
AS	1717	31	3	1	8	1	1	66,6	53,99	49,74	1x3	0	1	0,513
AS	1717	31	3	1	8	1	1	56,48	56,67	50,56	2x1	0	1	0,274
AS	1717	31	3	1	9	1	1	54,09	52,13	49,98	3x3	0	1	0,227
AS	1717	31	3	1	10	1	1	65,52	48,3	49,41	1x2	0	1	0,18
AS	1717	31	3	1	11	1	0							
AS	1717	31	3	1	12	1	0							
AS	1717	31	3	1	13	1	0							
AS	1717	31	3	1	14	1	0							
AS	1717	31	3	1	15	1	0							
AS	1722	33	1	2	0	1	1	62,88	48,98	68,85	2x2	0	1	0,376
AS	1722	33	1	2	1	1	0							
AS	1722	33	1	2	2	1	0							
AS	1722	33	1	2	3	1	0							
AS	1722	33	1	2	4	0								
AS	1722	33	1	2	5	0								
AS	1722	33	1	2	6	0								
AS	1722	33	1	2	7	0								
AS	1725	33	2	2	8	1	0							
AS	1725	33	2	2	9	1	0							
AS	1725	33	2	2	10	1	0							
AS	1725	33	2	2	11	1	0							
AS	1725	33	2	2	12	0								
AS	1725	33	2	2	13	0								
AS	1725	33	2	2	14	0								
AS	1725	33	2	2	15	0								
AS	1726	36	1	1	0	1	0							
AS	1726	36	1	1	1	1	0							
AS	1726	36	1	1	2	1	0							
AS	1726	36	1	1	3	1	0							
AS	1726	36	1	1	4	1	0							
AS	1726	36	1	1	5	1	0							
AS	1726	36	1	1	6	1	0							
AS	1726	36	1	1	7	1	0							
AS	1729	36	2	1	8	1	1	82,66	53,52	48,49	4x1	0	0	0,376
AS	1729	36	2	1	9	1	0							
AS	1729	36	2	1	10	1	0							
AS	1729	36	2	1	11	1	0							
AS	1729	36	2	1	12	1	0							
AS	1729	36	2	1	13	1	0							
AS	1729	36	2	1	14	1	0							
AS	1729	36	2	1	15	1	0							
AS	1732	37	0	1	0	1	1	79,72	54,08	81,69	1x5	0	1	0,792
AS	1732	37	0	1	1	1	0							
AS	1732	37	0	1	2	1	0							
AS	1732	37	0	1	3	1	0							
AS	1732	37	0	1	4	1	0							
AS	1732	37	0	1	5	1	1	73,18	49,24	95,55	4x3	0	1	0,241
AS	1732	37	0	1	6	1	0							
AS	1732	37	0	1	7	1	0							
AS	1735	37	3	1	8	1	1	53,3	55,72	46,23	8x5	0	0	0,961
AS	1735	37	3	1	9	1	1	50,49	52,7	40,74	1x2	0	1	0,289
AS	1735	37	3	1	10	1	0							
AS	1735	37	3	1	11	1	1	61,48	53,23	42,71	3x1	0	0	0,186
AS	1735	37	3	1	12	1	0							
AS	1735	37	3	1	13	1	0							
AS	1735	37	3	1	14	1	0							
AS	1735	37	3	1	15	1	1	60,78	49,7	51,73	7x9	1	0	0,992
AS	1738	41	1	1	0	1	1	56,3	55,7	89,06	3x2	0	1	0,306
AS	1738	41	1	1	1	1	0							
AS	1738	41	1	1	2	1	0							
AS	1738	41	1	1	3	1	0							
AS	1738	41	1	1	4	1	0							
AS	1738	41	1	1	5	1	1	53,54	50,6	102,02	2x3	0	1	0,164
AS	1738	41	1	1	6	1	1	57,48	43,52	101,03	1x4	0	1	0,28
AS	1738	41	1	1	7	1	0							
AS	1741	41	2	1	8	1	1	81,33	57,36	50,84	2x2	0	1	0,273
AS	1741	41	2	1	9	1	0							
AS	1741	41	2	1	10	1	0							
AS	1741	41	2	1	11	1	0							
AS	1741	41	2	1	12	1	0							
AS	1741	41	2	1	13	1	0							
AS	1741	41	2	1	14	1	0							
AS	1741	41	2	1	15	1	0							
AS	1744	42	0	1	0	0								
AS	1744	42	0	1	1	0								
AS	1744	42	0	1	2	0								
AS	1744	42	0	1	3	0								
AS	1744	42	0	1	4	1	0							
AS	1744	42	0	1	5	1	0							
AS	1744	42	0	1	6	1	0							
AS	1744	42	0	1	7	1	0							
AS	1747	42	3	1	8	1	1	62,6	58,18	53,08	2x1	0	0	0,173
AS	1747	42	3	1	8	1	1	62,78	57,76	52,83	2x2	0	1	0,285
AS	1747	42	3	1	9	1	1	55,06	55,92	53,74	2x1	0	1	0,209
AS	1747	42	3	1	10	1	1	64,21	51,57	53,57	2x1	0	0	0,242
AS	1747	42	3	1	11	1	0							
AS	1747	42	3	1	12	1	0							
AS	1747	42	3	1	13	1	0							
AS	1747	42	3	1	14	1	0							
AS	1747	42	3	1	15	1	0							
AS	1750	44	1	1	0	1	1	61,58	46,56	67,18	1x2	0	1	0,313
AS	1750	44	1	1	0	1	1	64,97	45,41	68	1x3	0	1	0,261
AS	1750	44	1	1	1	1	0							
AS	1750	44	1	1	2	1	0							
AS	1750	44	1	1	3	1	0							
AS	1750	44	1	1	4	1	1	62,72	43,88	77,26	7x2	0	0	0,41
AS	1750	44	1	1	5	1	0							
AS	1750	44	1	1	6	1	1	60,58	32,6	78,08	1x5	0	1	0,221
AS	1750	44	1	1	7	1	1	65,9	36,52	76,44	3x1	0	0	0,345
AS	1753	44	2	1	8	1	1	90,41	53,6	27,21	3x1	0	1	0,249
AS	1753	44	2	1	9	1	1	81,08	51,32	28,69	2x4	0	0	0,331
AS	1753	44	2	1	10	1	0							
AS	1753	44	2	1	11	1	0							
AS	1753	44	2	1	12	0								
AS	1753	44	2	1	13	0								
AS	1753	44	2	1	14	0								
AS	1753	44	2	1	15	0								
AS	1756	45	0	1	0	1	1	79,98	44,33	91,47	3x5	0	1	0,415
AS	1756	45	0	1	1	1	0							

AS	1756	45	0	1	2	1	0							
AS	1756	45	0	1	3	1	0							
AS	1756	45	0	1	4	1	0							
AS	1756	45	0	1	5	1	0							
AS	1756	45	0	1	6	1	0							
AS	1756	45	0	1	7	1	0							
AS	1759	45	3	1	8	1	1	61,43	53,35	54,38	4x1	0	1	0,196
AS	1759	45	3	1	8	1	1	59,96	54,19	55,77	1x2	0	0	0,157
AS	1759	45	3	1	8	1	1	56,76	55,38	56,27	2x1	0	1	0,24
AS	1759	45	3	1	9	1	0							
AS	1759	45	3	1	10	1	0							
AS	1759	45	3	1	11	1	0							
AS	1759	45	3	1	12	1	0							
AS	1759	45	3	1	13	1	0							
AS	1759	45	3	1	14	1	0							
AS	1759	45	3	1	15	1	0							

(width in mm)
