# Supplementary material for: Development of a scoring method to visually score cortical interruptions on high-resolution peripheral quantitative computed tomography in rheumatoid arthritis and healthy controls
Source: PLoS One. 2018 Jul 9;13(7):e0200331. doi: 10.1371/journal.pone.0200331 (PMC6037386; doi:10.1371/journal.pone.0200331)
Supplement: S2 File — (PDF) [file pone.0200331.s002.pdf]

| Reader initials | studyID | start time | joint (0=MCP2, 1= MCP3, 2=PIP2, 3=PIP3) | surface (0= palmar PB, 1=ul | evaluable (0=no 1=yes) | discontinuity (0=no 1=yes 2=total destruction) | x value | y value | z value | planes | adjacent trabecular distortion (0=no 1=yes) | parallel structure (0=no | maximal diameter | end time | total time |
|---|---|---|---|---|---|---|---|---|---|---|---|---|---|---|---|
| AS | 1300 | 9:26 | 3 | 8 | 1 | 1 | 57,65 | 51,78 | 42,11 | 5x3 | 0 | 1 | 0,51 | | |
| AS | | | | 8 | 1 | 1 | 54,61 | 53,37 | 43,01 | 4x3 | 0 | 1 | 0,662 | | |
| AS | | | | 9 | 1 | 1 | 49,77 | 53,12 | 39,98 | 2x2 | 0 | 1 | 0,147 | | |
| AS | | | | 10 | 1 | 0 | | | | | | | | | |
| AS | | | | 11 | 1 | 1 | 59,45 | 49,79 | 37,03 | 2x4 | 0 | 1 | 0,303 | | |
| AS | | | | 12 | 1 | 0 | | | | | | | | | |
| AS | | | | 13 | 1 | 0 | | | | | | | | | |
| AS | | | | 14 | 1 | 1 | 54,47 | 42,93 | 46,46 | 4x2 | 0 | 0 | 0,304 | | |
| AS | | | | 15 | 1 | 0 | | | | | | | | 9:38 | 12 |
| AS | 1233 | 9:51 | 3 | 8 | 1 | 1 | 59,27 | 52,11 | 37,26 | 5x5 | 0 | 0 | 0,393 | | |
| AS | | | | 8 | 1 | 1 | 56,81 | 53,69 | 39,96 | 5x4 | 0 | 0 | 0,312 | | |
| AS | | | | 9 | 1 | 1 | 48,55 | 50,82 | 41,11 | 3x5 | 1 | 0 | 0,397 | | |
| AS | | | | 9 | 1 | 1 | 49,07 | 51,9 | 42,26 | 4x7 | 0 | 0 | 0,745 | | |
| AS | | | | 10 | 1 | 1 | 57,5 | 47,15 | 40,45 | 15x23 | 1 | 0 | 1,827 | | |
| AS | | | | 10 | 1 | 1 | 50,66 | 47,74 | 41,27 | 3x10 | 0 | 0 | 0,668 | | |
| AS | | | | 11 | 1 | 1 | 59,12 | 48,4 | 39,72 | 6x6 | 0 | 0 | 0,701 | | |
| AS | | | | 12 | 1 | 0 | | | | | | | | | |
| AS | | | | 13 | 1 | 0 | | | | | | | | | |
| AS | | | | 14 | 1 | 1 | 56,84 | 45,72 | 44,14 | 15x11 | 1 | 0 | 1,273 | | |
| AS | | | | 15 | 1 | 1 | 59,94 | 49,98 | 46,36 | 11x2 | 1 | 0 | 0,99 | 10:08 | 17 |
| AS | 1261 | 11:04 | 1 | 0 | 1 | 1 | 59,29 | 53,54 | 85,46 | 3x2 | 0 | 1 | 0,408 | | |
| AS | | | | 0 | 1 | 1 | 57,87 | 53,71 | 85,38 | 3x1 | 0 | 1 | 0,43 | | |
| AS | | | | 0 | 1 | 1 | 54,17 | 54,94 | 85,87 | 2x3 | 0 | 1 | 0,327 | | |
| AS | | | | 1 | 1 | 0 | | | | | | | | | |
| AS | | | | 2 | 1 | 1 | 58,93 | 43,5 | 87,76 | 3x5 | 0 | 1 | 0,372 | | |
| AS | | | | 3 | 1 | 1 | 63,39 | 48,99 | 86,12 | 2x1 | 0 | 1 | 0,136 | | |
| AS | | | | 4 | 1 | 1 | 60,09 | 53,22 | 99,97 | 3x3 | 0 | 0 | 0,735 | | |
| AS | | | | 5 | 1 | 1 | 50,47 | 48,2 | 97,35 | 2x2 | 0 | 1 | 0,301 | | |
| AS | | | | 6 | 1 | 1 | 54,13 | 43,35 | 99,24 | 7x8 | 0 | 0 | 0,474 | | |
| AS | | | | 7 | 1 | 0 | | | | | | | | 11:21 | 17 |
| AS | 1232 | 11:23 | 1 | 0 | 1 | 0 | | | | | | | | | |
| AS | | | | 1 | 1 | 0 | | | | | | | | | |
| AS | | | | 2 | 1 | 0 | | | | | | | | | |
| AS | | | | 3 | 1 | 0 | | | | | | | | | |
| AS | | | | 4 | 1 | 0 | | | | | | | | | |
| AS | | | | 5 | 1 | 0 | | | | | | | | | |
| AS | | | | 6 | 1 | 0 | | | | | | | | | |
| AS | | | | 7 | 1 | 0 | | | | | | | | 11:34 | 11 |
| AS | 1226 | 11:34 | 2 | 8 | 1 | 1 | 81,91 | 51,39 | 33,16 | 4x1 | 0 | 0 | 0,68 | | |
| AS | | | | 9 | 1 | 0 | | | | | | | | | |
| AS | | | | 10 | 1 | 1 | 83,2 | 45,48 | 29,79 | 25x43 | 1 | 0 | 4,687 | | |
| AS | | | | 11 | 1 | 1 | 85,06 | 48,19 | 32,34 | 3x3 | 0 | 0 | 0,393 | | |
| AS | | | | 12 | 1 | 2 | | | | | | | | | |
| AS | | | | 13 | 1 | 2 | | | | | | | | | |
| AS | | | | 14 | 1 | 2 | | | | | | | | | |
| AS | | | | 15 | 1 | 2 | | | | | | | | 11:45 | 11 |
| AS | 1274 | 11:47 | 3 | 8 | 1 | 0 | | | | | | | | | |
| AS | | | | 9 | 1 | 1 | 65,86 | 47,2 | 54,64 | 1x2 | 0 | 0 | 0,27 | | |
| AS | | | | 10 | 1 | 0 | | | | | | | | | |
| AS | | | | 11 | 1 | 0 | | | | | | | | | |
| AS | | | | 12 | 1 | 1 | 59,35 | 51,42 | 60,38 | 2x1 | 0 | 1 | 0,256 | | |
| AS | | | | 13 | 1 | 0 | | | | | | | | | |
| AS | | | | 14 | 1 | 0 | | | | | | | | | |
| AS | | | | 15 | 1 | 0 | | | | | | | | 11:59 | 12 |
| AS | 1281 | 12:00 | 1 | 0 | 1 | 1 | 64,45 | 46,86 | 89,79 | 1x2 | 0 | 1 | 0,33 | | |
| AS | | | | 0 | 1 | 1 | 61,89 | 47,7 | 93,48 | 3x5 | 0 | 1 | 0,37 | | |
| AS | | | | 1 | 1 | 0 | | | | | | | | | |
| AS | | | | 2 | 1 | 0 | | | | | | | | | |
| AS | | | | 3 | 1 | 1 | 65,36 | 41,24 | 94,06 | 1x2 | 0 | 1 | 0,452 | | |
| AS | | | | 3 | 1 | 1 | 63,85 | 40,73 | 97,17 | 3x3 | 0 | 0 | 0,479 | | |
| AS | | | | 4 | 1 | 0 | | | | | | | | | |
| AS | | | | 5 | 1 | 1 | 52,44 | 41,32 | 102,5 | 2x4 | 0 | 1 | 0,492 | | |
| AS | | | | 6 | 1 | 0 | | | | | | | | | |
| AS | | | | 7 | 1 | 1 | 60,38 | 35,33 | 104,22 | 10x11 | 1 | 0 | 1,781 | 12:14 | 14 |
| AS | 1254 | 13:24 | 1 | 0 | 1 | 1 | 57,14 | 52,05 | 85,9 | 2x1 | 0 | 1 | 0,152 | | |
| AS | | | | 0 | 1 | 1 | 62,99 | 50,93 | 87,22 | 4x3 | 0 | 1 | 0,484 | | |
| AS | | | | 0 | 1 | 1 | 59,31 | 51,71 | 89,1 | 2x2 | 0 | 1 | 0,592 | | |
| AS | | | | 1 | 1 | 0 | | | | | | | | | |
| AS | | | | 2 | 1 | 1 | 57,55 | 40,59 | 89,18 | 2x2 | 0 | 1 | 0,29 | | |
| AS | | | | 2 | 1 | 1 | 58,56 | 41,67 | 90,74 | 2x5 | 0 | 1 | 0,438 | | |
| AS | | | | 2 | 1 | 1 | 61,74 | 41,64 | 90,99 | 11x42 | 1 | 0 | 1,299 | | |
| AS | | | | 3 | 1 | 0 | | | | | | | | | |
| AS | | | | 4 | 1 | 1 | 62,71 | 49,59 | 100,39 | 6x2 | 0 | 0 | 0,606 | | |
| AS | | | | 5 | 1 | 0 | | | | | | | | | |
| AS | | | | 6 | 1 | 0 | | | | | | | | | |
| AS | | | | 7 | 1 | 1 | 64,82 | 45,32 | 101,32 | 12x7 | 1 | 0 | 1,237 | | |
| AS | | | | 7 | 1 | 1 | 62,28 | 43,45 | 98,45 | 70x27 | 1 | 0 | 7,42 | 13:44 | 20 |
| AS | 1214 | 13:50 | 0 | 0 | 1 | 1 | 79,48 | 42,3 | 71,9 | 3x2 | 0 | 1 | 0,202 | | |
| AS | | | | 0 | 1 | 1 | 78,37 | 42,33 | 73,04 | 4x4 | 0 | 1 | 0,43 | | |
| AS | | | | 1 | 1 | 0 | | | | | | | | | |
| AS | | | | 2 | 1 | 0 | | | | | | | | | |
| AS | | | | 3 | 1 | 0 | | | | | | | | | |
| AS | | | | 4 | 1 | 1 | 79,66 | 39,35 | 84,28 | 3x8 | 0 | 1 | 0,359 | | |
| AS | | | | 5 | 1 | 0 | | | | | | | | | |
| AS | | | | 6 | 1 | 0 | | | | | | | | | |
| AS | | | | 7 | 1 | 0 | | | | | | | | 14:02 | 12 |
| AS | 1207 | 14:20 | 1 | 0 | 1 | 1 | 54,45 | 48,56 | 72,24 | 2x2 | 0 | 1 | 0,155 | | |
| AS | | | | 0 | 1 | 1 | 54,4 | 48,54 | 72,65 | 2x1 | 0 | 1 | 0,311 | | |
| AS | | | | 0 | 1 | 1 | 56,05 | 49,28 | 76,67 | 3x2 | 0 | 1 | 0,267 | | |
| AS | | | | 0 | 1 | 1 | 59,84 | 48,27 | 80,03 | 2x3 | 0 | 0 | 0,632 | | |
| AS | | | | 1 | 1 | 0 | | | | | | | | | |
| AS | | | | 2 | 1 | 0 | | | | | | | | | |
| AS | | | | 3 | 1 | 0 | | | | | | | | | |
| AS | | | | 4 | 1 | 0 | | | | | | | | | |
| AS | | | | 5 | 1 | 1 | 54,25 | 43,75 | 93,15 | 1x2 | 0 | 1 | 0,271 | | |
| AS | | | | 6 | 1 | 0 | | | | | | | | | |
| AS | | | | 7 | 1 | 1 | 62,64 | 40,93 | 87,33 | 7x5 | 1 | 0 | 1,298 | 14:36 | 16 |
| AS | 1269 | 14:37 | 0 | 0 | 1 | 1 | 46,19 | 50,29 | 69,08 | 1x3 | 0 | 1 | 0,269 | | |
| AS | | | | 1 | 1 | 0 | | | | | | | | | |
| AS | | | | 2 | 1 | 0 | | | | | | | | | |
| AS | | | | 3 | 1 | 0 | | | | | | | | | |
| AS | | | | 4 | 0 | | | | | | | | | | |
| AS | | | | 5 | 0 | | | | | | | | | | |
| AS | | | | 6 | 0 | | | | | | | | | | |
| AS | | | | 7 | 0 | | | | | | | | | 14:43 | 6 |
| AS | 1301 | 15:06 | 2 | 8 | 1 | 1 | 79,04 | 58,25 | 41,81 | 2x2 | 0 | 0 | 0,282 | | |
| AS | | | | 9 | 1 | 0 | | | | | | | | | |
| AS | | | | 10 | 1 | 1 | 77,78 | 50,64 | 41,89 | 6x4 | 0 | 0 | 0,527 | | |
| AS | | | | 11 | 1 | 0 | | | | | | | | | |
| AS | | | | 12 | 1 | 0 | | | | | | | | | |
| AS | | | | 13 | 1 | 0 | | | | | | | | | |
| AS | | | | 14 | 1 | 0 | | | | | | | | | |
| AS | | | | 15 | 1 | 0 | | | | | | | | 15:16 | 10 |
| AS | 1285 | 15:16 | 0 | 0 | 1 | 1 | 80,76 | 45,14 | 78,36 | 2x1 | 0 | 1 | 0,303 | | |
| AS | | | | 1 | 1 | 0 | | | | | | | | | |
| AS | | | | 2 | 1 | 0 | | | | | | | | | |
| AS | | | | 3 | 1 | 1 | 83,66 | 39,21 | 81,31 | 3x17 | 1 | 0 | 0,757 | | |
| AS | | | | 4 | 1 | 1 | 80,59 | 42,18 | 89,75 | 3x3 | 1 | 0 | 0,37 | | |
| AS | | | | 5 | 1 | 0 | | | | | | | | | |
| AS | | | | 6 | 1 | 0 | | | | | | | | | |
| AS | | | | 7 | 1 | 1 | 82,92 | 37,3 | 90,49 | 17x10 | 1 | 0 | 1,247 | 15:28 | 12 |
| AS | 1215 | 15:29 | 0 | 0 | 1 | 0 | | | | | | | | | |
| AS | | | | 1 | 1 | 0 | | | | | | | | | |
| AS | | | | 2 | 1 | 0 | | | | | | | | | |
| AS | | | | 3 | 1 | 1 | 35,38 | 42,39 | 68,87 | 16x2 | 0 | 0 | 1,931 | | |
| AS | | | | 4 | 1 | 1 | 40,89 | 36,44 | 76,74 | 3x3 | 0 | 0 | 0,358 | | |
| AS | | | | 5 | 1 | 1 | 46,32 | 32,11 | 75,02 | 9x5 | 0 | 0 | 0,454 | | |
| AS | | | | 6 | 1 | 0 | | | | | | | | | |
| AS | | | | 7 | 1 | 1 | 37,08 | 33,04 | 77,31 | 23x43 | 1 | 0 | 2,849 | 15:42 | 13 |
| AS | 1288 | 8:23 | 0 | 0 | 1 | 1 | 78,79 | 52,51 | 88,08 | 3x5 | 0 | 1 | 0,371 | | |
| AS | | | | 1 | 1 | 1 | 71,17 | 50,01 | 88 | 2x1 | 0 | 1 | 0,19 | | |
| AS | | | | 2 | 1 | 0 | | | | | | | | | |
| AS | | | | 3 | 1 | 0 | | | | | | | | | |
| AS | | | | 4 | 1 | 1 | 78,61 | 51,03 | 98,66 | 2x1 | 0 | 1 | 0,288 | | |
| AS | | | | 5 | 1 | 1 | 72,43 | 48,5 | 97,43 | 2x1 | 0 | 1 | 0,181 | | |
| AS | | | | 6 | 1 | 0 | | | | | | | | | |
| AS | | | | 7 | 1 | 0 | | | | | | | | 8:36 | 13 |
| AS | 1238 | 8:36 | 2 | 8 | 1 | 0 | | | | | | | | | |
| AS | | | | 9 | 1 | 0 | | | | | | | | | |
| AS | | | | 10 | 1 | 0 | | | | | | | | | |
| AS | | | | 11 | 1 | 0 | | | | | | | | | |
| AS | | | | 12 | 1 | 0 | | | | | | | | | |
| AS | | | | 13 | 1 | 0 | | | | | | | | | |
| AS | | | | 14 | 1 | 1 | 80,52 | 49,72 | 57,83 | 4x2 | 0 | 1 | 0,225 | | |
| AS | | | | 15 | 1 | 0 | | | | | | | | 8:43 | 7 |
| AS | 1243 | 8:43 | 0 | 0 | 1 | 0 | | | | | | | | | |
| AS | | | | 1 | 1 | 0 | | | | | | | | | |
| AS | | | | 2 | 1 | 0 | | | | | | | | | |
| AS | | | | 3 | 1 | 0 | | | | | | | | | |
| AS | | | | 4 | 1 | 1 | 44,13 | 48,34 | 87,26 | 4x2 | 0 | 1 | 0,392 | | |
| AS | | | | 5 | 1 | 1 | 48,6 | 42,09 | 85,95 | 2x2 | 0 | 1 | 0,113 | | |
| AS | | | | 6 | 1 | 0 | | | | | | | | | |
| AS | | | | 7 | 1 | 0 | | | | | | | | 8:50 | 7 |
| AS | 1259 | 8:56 | 1 | 0 | 1 | 1 | 58,57 | 49,41 | 86,99 | 5x3 | 0 | 1 | 0,456 | | |
| AS | | | | 1 | 1 | 0 | | | | | | | | | |
| AS | | | | 2 | 1 | 0 | | | | | | | | | |
| AS | | | | 3 | 1 | 0 | | | | | | | | | |
| AS | | | | 4 | 1 | 0 | | | | | | | | | |
| AS | | | | 5 | 1 | 1 | 51,53 | 45,06 | 97,4 | 1x2 | 0 | 1 | 0,297 | | |

| | | | | | | | | | | | | | | | |
|---|---|---|---|---|---|---|---|---|---|---|---|---|---|---|---|
| AS | | | | 6 | 1 | 0 | | | | | | | | | |
| AS | | | | 7 | 1 | 0 | | | | | | | | 9:04 | 8 |
| AS | 1240 | 9:08 | 1 | 0 | 1 | 0 | | | | | | | | | |
| AS | | | | 1 | 1 | 0 | | | | | | | | | |
| AS | | | | 2 | 1 | 0 | | | | | | | | | |
| AS | | | | 3 | 1 | 0 | | | | | | | | | |
| AS | | | | 4 | 1 | 0 | | | | | | | | | |
| AS | | | | 5 | 1 | 0 | | | | | | | | | |
| AS | | | | 6 | 1 | 0 | | | | | | | | | |
| AS | | | | 7 | 1 | 1 | 62,02 | 47,71 | 95,72 | 10x8 | 1 | 0 | 0,948 | 9:15 | 7 |
| AS | 1260 | 9:16 | 3 | 8 | 1 | 1 | 66,81 | 54,92 | 26,47 | 4x4 | 0 | 0 | 1,006 | | |
| AS | | | | 9 | 1 | 0 | | | | | | | | | |
| AS | | | | 10 | 1 | 0 | | | | | | | | | |
| AS | | | | 11 | 1 | 0 | | | | | | | | | |
| AS | | | | 12 | 1 | 0 | | | | | | | | | |
| AS | | | | 13 | 1 | 0 | | | | | | | | | |
| AS | | | | 14 | 1 | 0 | | | | | | | | | |
| AS | | | | 15 | 1 | 0 | | | | | | | | 9:22 | 6 |
| AS | 1253 | 9:43 | 1 | 0 | 1 | 0 | | | | | | | | | |
| AS | | | | 1 | 1 | 0 | | | | | | | | | |
| AS | | | | 2 | 1 | 0 | | | | | | | | | |
| AS | | | | 3 | 1 | 0 | | | | | | | | | |
| AS | | | | 4 | 1 | 0 | | | | | | | | | |
| AS | | | | 5 | 1 | 1 | 50,68 | 43,66 | 105,41 | 1x2 | 0 | 1 | 0,222 | | |
| AS | | | | 6 | 1 | 1 | 55,72 | 36,27 | 106,14 | 2x2 | 0 | 1 | 0,392 | | |
| AS | | | | 7 | 1 | 0 | | | | | | | | 9:51 | 8 |
| AS | 1252 | 9:52 | 2 | 8 | 1 | 1 | 78,56 | 57,09 | 39,46 | 3x3 | 0 | 1 | 0,348 | | |
| AS | | | | 8 | 1 | 1 | 76,21 | 57,84 | 40,45 | 3x3 | 0 | 1 | 0,368 | | |
| AS | | | | 9 | 1 | 0 | | | | | | | | | |
| AS | | | | 10 | 1 | 0 | | | | | | | | | |
| AS | | | | 11 | 1 | 1 | 83,65 | 53,7 | 37,41 | 2x2 | 0 | 1 | 0,267 | | |
| AS | | | | 12 | 1 | 0 | | | | | | | | | |
| AS | | | | 13 | 1 | 1 | 73,08 | 56,54 | 45,86 | 3x1 | 0 | 0 | 0,493 | | |
| AS | | | | 14 | 1 | 0 | | | | | | | | | |
| AS | | | | 15 | 1 | 0 | | | | | | | | 10:02 | 10 |
| AS | 1210 | 10:11 | 3 | 8 | 1 | 0 | | | | | | | | | |
| AS | | | | 9 | 1 | 0 | | | | | | | | | |
| AS | | | | 10 | 1 | 0 | | | | | | | | | |
| AS | | | | 11 | 1 | 0 | | | | | | | | | |
| AS | | | | 12 | 1 | 0 | | | | | | | | | |
| AS | | | | 13 | 1 | 1 | 71,29 | 55,3 | 48,67 | 24x10 | 1 | 0 | 0,781 | | |
| AS | | | | 14 | 1 | 0 | | | | | | | | | |
| AS | | | | 15 | 1 | 0 | | | | | | | | 10:17 | 6 |
| AS | 1204 | 10:19 | 1 | 0 | 1 | 1 | 62,25 | 53,64 | 92,57 | 3x3 | 0 | 1 | 0,252 | | |
| AS | | | | 0 | 1 | 1 | 64,71 | 53,77 | 93,3 | 3x5 | 0 | 1 | 0,422 | | |
| AS | | | | 0 | 1 | 1 | 62,88 | 53,73 | 95,44 | 17x23 | 1 | 0 | 2,299 | | |
| AS | | | | 1 | 1 | 0 | | | | | | | | | |
| AS | | | | 2 | 1 | 0 | | | | | | | | | |
| AS | | | | 3 | 1 | 0 | | | | | | | | | |
| AS | | | | 4 | 1 | 0 | | | | | | | | | |
| AS | | | | 5 | 1 | 1 | 66,44 | 51,19 | 104,37 | 11x15 | 0 | 0 | 1,042 | | |
| AS | | | | 6 | 1 | 0 | | | | | | | | | |
| AS | | | | 7 | 1 | 1 | 54,61 | 48,51 | 106,34 | 11x9 | 1 | 0 | 0,626 | | |
| AS | | | | 7 | 1 | 1 | 56,43 | 45,67 | 105,03 | 10x9 | 1 | 0 | 1,068 | 10:32 | 13 |
| AS | 1248 | 11:08 | 1 | 0 | 1 | 1 | 58,88 | 50,82 | 76 | 1x2 | 0 | 1 | 0,292 | | |
| AS | | | | 0 | 1 | 1 | 62,78 | 50,29 | 76,74 | 3x2 | 0 | 1 | 0,512 | | |
| AS | | | | 1 | 1 | 0 | | | | | | | | | |
| AS | | | | 2 | 1 | 0 | | | | | | | | | |
| AS | | | | 3 | 1 | 0 | | | | | | | | | |
| AS | | | | 4 | 1 | 0 | | | | | | | | | |
| AS | | | | 5 | 1 | 1 | 56,07 | 45,06 | 87,81 | 6x6 | 0 | 1 | 0,534 | | |
| AS | | | | 6 | 1 | 1 | 59,11 | 38,88 | 87,89 | 6x4 | 0 | 1 | 0,432 | | |
| AS | | | | 7 | 1 | 0 | | | | | | | | 11:18 | 10 |
| AS | 1246 | 11:18 | 3 | 8 | 1 | 1 | 55,55 | 59,12 | 31,68 | 2x2 | 0 | 0 | 0,262 | | |
| AS | | | | 9 | 1 | 0 | | | | | | | | | |
| AS | | | | 10 | 1 | 0 | | | | | | | | | |
| AS | | | | 11 | 1 | 0 | | | | | | | | | |
| AS | | | | 12 | 1 | 0 | | | | | | | | | |
| AS | | | | 13 | 1 | 1 | 51,73 | 56,87 | 36,92 | 12x8 | 1 | 0 | 0,505 | | |
| AS | | | | 14 | 1 | 0 | | | | | | | | | |
| AS | | | | 15 | 1 | 0 | | | | | | | | 11:25 | 7 |
| AS | 1220 | 11:25 | 3 | 8 | 1 | 1 | 62,59 | 51,4 | 40,74 | 6x2 | 1 | 0 | 1,115 | | |
| AS | | | | 9 | 1 | 0 | | | | | | | | | |
| AS | | | | 10 | 1 | 0 | | | | | | | | | |
| AS | | | | 11 | 1 | 0 | | | | | | | | | |
| AS | | | | 12 | 1 | 0 | | | | | | | | | |
| AS | | | | 13 | 1 | 0 | | | | | | | | | |
| AS | | | | 14 | 1 | 0 | | | | | | | | | |
| AS | | | | 15 | 1 | 0 | | | | | | | | 11:32 | 7 |
| AS | 1249 | 11:34 | 3 | 8 | 1 | 1 | 63,37 | 54,93 | 40,83 | 2x1 | 0 | 1 | 0,273 | | |
| AS | | | | 9 | 1 | 0 | | | | | | | | | |
| AS | | | | 10 | 1 | 0 | | | | | | | | | |
| AS | | | | 11 | 1 | 0 | | | | | | | | | |
| AS | | | | 12 | 1 | 0 | | | | | | | | | |
| AS | | | | 13 | 1 | 0 | | | | | | | | | |
| AS | | | | 14 | 1 | 0 | | | | | | | | | |
| AS | | | | 15 | 1 | 1 | 65,07 | 51,09 | 45,75 | 18x12 | 1 | 0 | 0,977 | 11:40 | 6 |
| AS | 1283 | 11:45 | 1 | 0 | 0 | | | | | | | | | | |
| AS | | | | 1 | 0 | | | | | | | | | | |
| AS | | | | 2 | 0 | | | | | | | | | | |
| AS | | | | 3 | 0 | | | | | | | | | | |
| AS | | | | 4 | 0 | | | | | | | | | | |
| AS | | | | 5 | 0 | | | | | | | | | | |
| AS | | | | 6 | 0 | | | | | | | | | | |
| AS | | | | 7 | 0 | | | | | | | | | 11:46 | 1 |
| AS | 1239 | 11:47 | 2 | 8 | 1 | 0 | | | | | | | | | |
| AS | | | | 9 | 1 | 0 | | | | | | | | | |
| AS | | | | 10 | 1 | 1 | 37,55 | 51,79 | 32,41 | 2x1 | 0 | 1 | 0,142 | | |
| AS | | | | 11 | 1 | 0 | | | | | | | | | |
| AS | | | | 12 | 1 | 0 | | | | | | | | | |
| AS | | | | 13 | 1 | 0 | | | | | | | | | |
| AS | | | | 14 | 1 | 0 | | | | | | | | | |
| AS | | | | 15 | 1 | 0 | | | | | | | | 11:53 | 6 |
| AS | 1276 | 11:53 | 2 | 8 | 1 | 0 | | | | | | | | | |
| AS | | | | 9 | 1 | 0 | | | | | | | | | |
| AS | | | | 10 | 1 | 0 | | | | | | | | | |
| AS | | | | 11 | 1 | 0 | | | | | | | | | |
| AS | | | | 12 | 1 | 0 | | | | | | | | | |
| AS | | | | 13 | 1 | 0 | | | | | | | | | |
| AS | | | | 14 | 1 | 0 | | | | | | | | | |
| AS | | | | 15 | 1 | 0 | | | | | | | | 11:58 | 5 |
| AS | 1205 | 11:59 | 3 | 8 | 1 | 0 | | | | | | | | | |
| AS | | | | 9 | 1 | 0 | | | | | | | | | |
| AS | | | | 10 | 1 | 0 | | | | | | | | | |
| AS | | | | 11 | 1 | 0 | | | | | | | | | |
| AS | | | | 12 | 1 | 0 | | | | | | | | | |
| AS | | | | 13 | 1 | 1 | 51,07 | 53,48 | 39,42 | 9x9 | 1 | 0 | 0,475 | | |
| AS | | | | 14 | 1 | 0 | | | | | | | | | |
| AS | | | | 15 | 1 | 1 | 61,75 | 52 | 41,47 | 10x15 | 1 | 0 | 0,66 | 12:06 | 7 |
| AS | 1296 | 13:13 | 3 | 8 | 0 | | | | | | | | | | |
| AS | | | | 9 | 0 | | | | | | | | | | |
| AS | | | | 10 | 0 | | | | | | | | | | |
| AS | | | | 11 | 0 | | | | | | | | | | |
| AS | | | | 12 | 1 | 0 | | | | | | | | | |
| AS | | | | 13 | 1 | 0 | | | | | | | | | |
| AS | | | | 14 | 1 | 0 | | | | | | | | | |
| AS | | | | 15 | 1 | 0 | | | | | | | | 13:18 | 5 |
| AS | 1200 | 13:18 | 0 | 0 | 1 | 1 | 83,14 | 46,18 | 94,5 | 3x3 | 0 | 1 | 0,449 | | |
| AS | | | | 0 | 1 | 1 | 82,21 | 45,91 | 98,19 | 6x7 | 0 | 0 | 0,965 | | |
| AS | | | | 1 | 1 | 0 | | | | | | | | | |
| AS | | | | 2 | 1 | 0 | | | | | | | | | |
| AS | | | | 3 | 1 | 1 | 90,48 | 41,33 | 93,35 | 4x2 | 0 | 1 | 0,381 | | |
| AS | | | | 3 | 1 | 1 | 89,92 | 43,88 | 95,97 | 14x19 | 0 | 0 | 1,783 | | |
| AS | | | | 4 | 1 | 1 | 86,14 | 40,2 | 105,65 | 5x4 | 0 | 1 | 0,66 | | |
| AS | | | | 5 | 1 | 1 | 79,14 | 36,64 | 103,76 | 2x1 | 0 | 1 | 0,268 | | |
| AS | | | | 6 | 1 | 1 | 81,25 | 29,6 | 102,86 | 3x5 | 0 | 0 | 0,874 | | |
| AS | | | | 7 | 1 | 1 | 89,57 | 35,6 | 106,8 | 35x23 | 1 | 0 | 3,155 | | |
| AS | | | | 7 | 1 | 1 | 86,77 | 32,9 | 100,65 | 18x8 | 1 | 0 | 1,907 | 13:35 | 17 |
| AS | 1216 | 13:37 | 2 | 8 | 1 | 0 | | | | | | | | | |
| AS | | | | 9 | 1 | 0 | | | | | | | | | |
| AS | | | | 10 | 1 | 0 | | | | | | | | | |
| AS | | | | 11 | 1 | 0 | | | | | | | | | |
| AS | | | | 12 | 1 | 0 | | | | | | | | | |
| AS | | | | 13 | 1 | 0 | | | | | | | | | |
| AS | | | | 14 | 1 | 0 | | | | | | | | | |
| AS | | | | 15 | 1 | 0 | | | | | | | | 13:46 | 9 |
| AS | 1287 | 13:47 | 1 | 0 | 1 | 0 | | | | | | | | | |
| AS | | | | 1 | 1 | 0 | | | | | | | | | |
| AS | | | | 2 | 1 | 0 | | | | | | | | | |
| AS | | | | 3 | 1 | 0 | | | | | | | | | |
| AS | | | | 4 | 1 | 0 | | | | | | | | | |
| AS | | | | 5 | 1 | 0 | | | | | | | | | |
| AS | | | | 6 | 1 | 0 | | | | | | | | | |
| AS | | | | 7 | 1 | 1 | 65,08 | 41,61 | 82,24 | 10x9 | 1 | 0 | 1,166 | 13:55 | 8 |
| AS | 1268 | 14:03 | 0 | 0 | 1 | 1 | 82,43 | 49,54 | 87,71 | 7x5 | 0 | 1 | 0,726 | | |
| AS | | | | 0 | 1 | 1 | 78,92 | 49,02 | 88,94 | 5x6 | 0 | 1 | 0,468 | | |
| AS | | | | 1 | 1 | 0 | | | | | | | | | |
| AS | | | | 2 | 1 | 0 | | | | | | | | | |
| AS | | | | 3 | 1 | 1 | 85,82 | 42,74 | 85,17 | 1x2 | 0 | 1 | 0,242 | | |
| AS | | | | 4 | 1 | 0 | | | | | | | | | |

| | | | | | | | | | | | | | | | |
|---|---|---|---|---|---|---|---|---|---|---|---|---|---|---|---|
| AS | | | | 5 | 1 | 0 | | | | | | | | | |
| AS | | | | 6 | 1 | 0 | | | | | | | | | |
| AS | | | | 7 | 1 | 0 | | | | | | | | 14:09 | 6 |
| AS | 1277 | 14:09 | 3 | 8 | 1 | 1 | 65,36 | 54,96 | 42,8 | 2x1 | 0 | 1 | 0,252 | | |
| AS | | | | 8 | 1 | 1 | 61,05 | 53,29 | 42,72 | 2x1 | 0 | 1 | 0,267 | | |
| AS | | | | 9 | 1 | 1 | 72,35 | 53,29 | 42,15 | 2x1 | 0 | 1 | 0,264 | | |
| AS | | | | 9 | 1 | 1 | 72 | 52,83 | 44,2 | 5x17 | 0 | 0 | 1,702 | | |
| AS | | | | 10 | 1 | 0 | | | | | | | | | |
| AS | | | | 11 | 1 | 0 | | | | | | | | | |
| AS | | | | 12 | 1 | 0 | | | | | | | | | |
| AS | | | | 13 | 1 | 0 | | | | | | | | | |
| AS | | | | 14 | 1 | 1 | 62,59 | 45,57 | 49,94 | 5x12 | 1 | 0 | 1,598 | | |
| AS | | | | 15 | 1 | 0 | | | | | | | | 14:21 | 12 |
| AS | 1293 | 14:26 | 1 | 0 | 1 | 0 | | | | | | | | | |
| AS | | | | 1 | 1 | 0 | | | | | | | | | |
| AS | | | | 2 | 1 | 1 | 59,21 | 44,55 | 87,3 | 8x8 | 1 | 0 | 0,984 | | |
| AS | | | | 3 | 1 | 0 | | | | | | | | | |
| AS | | | | 4 | 1 | 1 | 60,54 | 50,13 | 96,07 | 3x4 | 0 | 1 | 0,551 | | |
| AS | | | | 5 | 1 | 1 | 68,44 | 47,26 | 95,99 | 2x2 | 0 | 1 | 0,255 | | |
| AS | | | | 5 | 1 | 1 | 70,52 | 43,24 | 91,07 | 11x14 | 1 | 0 | 0,719 | | |
| AS | | | | 6 | 1 | 1 | 64,88 | 40,3 | 91,15 | 15x16 | 1 | 0 | 2,231 | | |
| AS | | | | 7 | 1 | 1 | 60,7 | 43,34 | 92,06 | 51x22 | 1 | 0 | 4,392 | 14:39 | 13 |
| AS | 1292 | 14:40 | 2 | 8 | 1 | 0 | | | | | | | | | |
| AS | | | | 9 | 1 | 0 | | | | | | | | | |
| AS | | | | 10 | 1 | 1 | 28,92 | 51,38 | 38,87 | 2x1 | 0 | 1 | 0,135 | | |
| AS | | | | 11 | 1 | 0 | | | | | | | | | |
| AS | | | | 12 | 1 | 0 | | | | | | | | | |
| AS | | | | 13 | 1 | 0 | | | | | | | | | |
| AS | | | | 14 | 1 | 0 | | | | | | | | | |
| AS | | | | 15 | 1 | 1 | 28,9 | 53,66 | 44,2 | 24x35 | 1 | 0 | 2,848 | 14:47 | 7 |
| AS | 1235 | 15:05 | 1 | 0 | 1 | 2 | | | | | | | | | |
| AS | | | | 1 | 1 | 2 | | | | | | | | | |
| AS | | | | 2 | 1 | 2 | | | | | | | | | |
| AS | | | | 3 | 1 | 2 | | | | | | | | | |
| AS | | | | 4 | 1 | 2 | | | | | | | | | |
| AS | | | | 5 | 1 | 2 | | | | | | | | | |
| AS | | | | 6 | 1 | 2 | | | | | | | | | |
| AS | | | | 7 | 1 | 2 | | | | | | | | 15:06 | 1 |
| AS | 1272 | 15:06 | 1 | 0 | 1 | 1 | 66,59 | 52,55 | 87,38 | 1x3 | 0 | 1 | 0,424 | | |
| AS | | | | 1 | 1 | 0 | | | | | | | | | |
| AS | | | | 2 | 1 | 1 | 62,25 | 42,34 | 89,02 | 7x2 | 0 | 1 | 0,59 | | |
| AS | | | | 3 | 1 | 1 | 58,64 | 46,93 | 90,82 | 17x20 | 1 | 0 | 1,481 | | |
| AS | | | | 4 | 1 | 0 | | | | | | | | | |
| AS | | | | 5 | 1 | 1 | 69,61 | 46,89 | 99,18 | 4x4 | 0 | 1 | 0,363 | | |
| AS | | | | 6 | 1 | 0 | | | | | | | | | |
| AS | | | | 7 | 1 | 1 | 60,75 | 44,69 | 97,54 | 20x22 | 1 | 0 | 2,278 | 15:21 | 15 |
| AS | 1258 | 15:21 | 1 | 0 | 1 | 1 | 58,72 | 55,47 | 93,68 | 2x1 | 0 | 1 | 0,172 | | |
| AS | | | | 1 | 1 | 0 | | | | | | | | | |
| AS | | | | 2 | 1 | 0 | | | | | | | | | |
| AS | | | | 3 | 1 | 0 | | | | | | | | | |
| AS | | | | 4 | 1 | 0 | | | | | | | | | |
| AS | | | | 5 | 1 | 0 | | | | | | | | | |
| AS | | | | 6 | 1 | 0 | | | | | | | | | |
| AS | | | | 7 | 1 | 0 | | | | | | | | 15:28 | 7 |
| AS | 1211 | 15:38 | 2 | 8 | 1 | 0 | | | | | | | | | |
| AS | | | | 9 | 1 | 0 | | | | | | | | | |
| AS | | | | 10 | 1 | 0 | | | | | | | | | |
| AS | | | | 11 | 1 | 0 | | | | | | | | | |
| AS | | | | 12 | 1 | 0 | | | | | | | | | |
| AS | | | | 13 | 1 | 0 | | | | | | | | | |
| AS | | | | 14 | 1 | 0 | | | | | | | | | |
| AS | | | | 15 | 1 | 0 | | | | | | | | 15:43 | 5 |
| AS | 1278 | 15:46 | 0 | 0 | 1 | 0 | | | | | | | | | |
| AS | | | | 1 | 1 | 1 | 71,92 | 40,05 | 82,8 | 3x1 | 0 | 1 | 0,592 | | |
| AS | | | | 2 | 1 | 0 | | | | | | | | | |
| AS | | | | 3 | 1 | 1 | 84,36 | 38,85 | 81,98 | 17x50 | 1 | 0 | 2,908 | | |
| AS | | | | 4 | 1 | 0 | | | | | | | | | |
| AS | | | | 5 | 1 | 1 | 71,68 | 35,34 | 87,31 | 8x7 | 0 | 0 | 0,568 | | |
| AS | | | | 6 | 1 | 1 | 75,48 | 32,74 | 90367 | 4x2 | 0 | 1 | 0,246 | | |
| AS | | | | 6 | 1 | 1 | 75,89 | 32,18 | 89,03 | 18x20 | 1 | 0 | 0,876 | | |
| AS | | | | 7 | 1 | 1 | 82,31 | 36,72 | 88,79 | 5x6 | 0 | 1 | 0,481 | 16:04 | 18 |
| AS | 1294 | 16:08 | 1 | 0 | 0 | | | | | | | | | | |
| AS | | | | 1 | 0 | | | | | | | | | | |
| AS | | | | 2 | 0 | | | | | | | | | | |
| AS | | | | 3 | 0 | | | | | | | | | | |
| AS | | | | 4 | 1 | 0 | | | | | | | | | |
| AS | | | | 5 | 1 | 0 | | | | | | | | | |
| AS | | | | 6 | 1 | 0 | | | | | | | | | |
| AS | | | | 7 | 1 | 0 | | | | | | | | 16:12 | 4 |
| AS | 1299 | 16:13 | 0 | 0 | 1 | 1 | 44,87 | 41,83 | 72,35 | 5x5 | 0 | 1 | 0,431 | | |
| AS | | | | 0 | 1 | 1 | 46,73 | 41,26 | 72,43 | 1x2 | 0 | 1 | 0,297 | | |
| AS | | | | 1 | 1 | 0 | | | | | | | | | |
| AS | | | | 2 | 1 | 0 | | | | | | | | | |
| AS | | | | 3 | 1 | 0 | | | | | | | | | |
| AS | | | | 4 | 1 | 0 | | | | | | | | | |
| AS | | | | 5 | 1 | 0 | | | | | | | | | |
| AS | | | | 6 | 1 | 0 | | | | | | | | | |
| AS | | | | 7 | 1 | 0 | | | | | | | | 16:22 | 9 |
| AS | 1245 | 16:23 | 3 | 8 | 1 | 0 | | | | | | | | | |
| AS | | | | 9 | 1 | 0 | | | | | | | | | |
| AS | | | | 10 | 1 | 0 | | | | | | | | | |
| AS | | | | 11 | 1 | 0 | | | | | | | | | |
| AS | | | | 12 | 1 | 0 | | | | | | | | | |
| AS | | | | 13 | 1 | 1 | 49,45 | 54,51 | 37,25 | 9x10 | 1 | 0 | 0,456 | | |
| AS | | | | 14 | 1 | 0 | | | | | | | | | |
| AS | | | | 15 | 1 | 0 | | | | | | | | 16:29 | 6 |
| AS | 1222 | 9:21 | 1 | 0 | 1 | 0 | | | | | | | | | |
| AS | | | | 1 | 1 | 1 | 71,36 | 50,94 | 80,78 | 1x2 | 0 | 1 | 0,115 | | |
| AS | | | | 2 | 1 | 0 | | | | | | | | | |
| AS | | | | 3 | 1 | 0 | | | | | | | | | |
| AS | | | | 4 | 1 | 0 | | | | | | | | | |
| AS | | | | 5 | 1 | 0 | | | | | | | | | |
| AS | | | | 6 | 1 | 1 | 66,31 | 42,84 | 97,51 | 2x1 | 0 | 1 | 0,4 | | |
| AS | | | | 7 | 1 | 1 | 60,83 | 48,08 | 96,61 | 13x16 | 1 | 0 | 1,66 | | |
| AS | | | | 7 | 1 | 1 | 63,09 | 46,34 | 92,75 | 12x7 | 1 | 0 | 0,567 | 9:35 | 14 |
| AS | 1265 | 9:39 | 2 | 8 | 1 | 0 | | | | | | | | | |
| AS | | | | 9 | 1 | 0 | | | | | | | | | |
| AS | | | | 10 | 1 | 0 | | | | | | | | | |
| AS | | | | 11 | 1 | 0 | | | | | | | | | |
| AS | | | | 12 | 1 | 0 | | | | | | | | | |
| AS | | | | 13 | 1 | 0 | | | | | | | | | |
| AS | | | | 14 | 1 | 0 | | | | | | | | | |
| AS | | | | 15 | 1 | 1 | 83,21 | 46,24 | 48,04 | 1x5 | 0 | 0 | 0,422 | 9:47 | 8 |
| AS | 1217 | 9:48 | 2 | 8 | 1 | 0 | | | | | | | | | |
| AS | | | | 9 | 1 | 1 | 46,26 | 53,94 | 40,44 | 2x1 | 0 | 1 | 0,183 | | |
| AS | | | | 9 | 1 | 1 | 45,81 | 52,25 | 42,32 | 2x3 | 0 | 0 | 0,332 | | |
| AS | | | | 10 | 1 | 1 | 39,99 | 48,14 | 42,57 | 2x3 | 0 | 0 | 0,469 | | |
| AS | | | | 11 | 1 | 0 | | | | | | | | | |
| AS | | | | 12 | 1 | 1 | 43,62 | 51,93 | 50,03 | 2x2 | 0 | 0 | 0,265 | | |
| AS | | | | 13 | 1 | 1 | 45,74 | 51,94 | 47,08 | 3x3 | 0 | 0 | 0,617 | | |
| AS | | | | 14 | 1 | 1 | 39,61 | 47,99 | 46,01 | 2x2 | 0 | 1 | 0,164 | | |
| AS | | | | 15 | 1 | 1 | 35,08 | 54,48 | 48,72 | 19x11 | 1 | 0 | 0,715 | | |
| AS | | | | 15 | 1 | 1 | 35,38 | 51,55 | 45,85 | 17x11 | 1 | 0 | 1,275 | 10:03 | 15 |
| AS | 1221 | 10:25 | 1 | 0 | 1 | 1 | 69,75 | 35,66 | 80,56 | 5x1 | 0 | 1 | 0,315 | | |
| AS | | | | 1 | 1 | 0 | | | | | | | | | |
| AS | | | | 2 | 1 | 0 | | | | | | | | | |
| AS | | | | 3 | 1 | 0 | | | | | | | | | |
| AS | | | | 4 | 1 | 1 | 73,65 | 31,88 | 79,99 | 2x1 | 0 | 1 | 0,25 | | |
| AS | | | | 5 | 1 | 0 | | | | | | | | | |
| AS | | | | 6 | 1 | 0 | | | | | | | | | |
| AS | | | | 7 | 1 | 1 | 69,65 | 28,44 | 92,12 | 8x11 | 1 | 0 | 0,564 | 10:37 | 12 |
| AS | 1231 | 10:58 | 0 | 0 | 1 | 0 | | | | | | | | | |
| AS | | | | 1 | 1 | 0 | | | | | | | | | |
| AS | | | | 2 | 1 | 0 | | | | | | | | | |
| AS | | | | 3 | 1 | 0 | | | | | | | | | |
| AS | | | | 4 | 1 | 1 | 81,95 | 35,16 | 75,38 | 1x4 | 0 | 1 | 0,284 | | |
| AS | | | | 5 | 1 | 1 | 74,96 | 31,23 | 74,81 | 2x2 | 0 | 1 | 0,152 | | |
| AS | | | | 6 | 1 | 1 | 76,1 | 25,11 | 73,5 | 3x1 | 0 | 0 | 0,522 | | |
| AS | | | | 7 | 1 | 0 | | | | | | | | 11:09 | 11 |
| AS | 1251 | 11:12 | 0 | 0 | 1 | 0 | | | | | | | | | |
| AS | | | | 1 | 1 | 0 | | | | | | | | | |
| AS | | | | 2 | 1 | 0 | | | | | | | | | |
| AS | | | | 3 | 1 | 0 | | | | | | | | | |
| AS | | | | 4 | 1 | 0 | | | | | | | | | |
| AS | | | | 5 | 1 | 0 | | | | | | | | | |
| AS | | | | 6 | 1 | 0 | | | | | | | | | |
| AS | | | | 7 | 1 | 0 | | | | | | | | 11:19 | 7 |
| AS | 1224 | 11:20 | 0 | 0 | 1 | 0 | | | | | | | | | |
| AS | | | | 1 | 1 | 0 | | | | | | | | | |
| AS | | | | 2 | 1 | 0 | | | | | | | | | |
| AS | | | | 3 | 1 | 1 | 83,72 | 43,44 | 80,52 | 10x7 | 1 | 0 | 1,264 | | |
| AS | | | | 4 | 1 | 2 | | | | | | | | | |
| AS | | | | 5 | 1 | 2 | | | | | | | | | |
| AS | | | | 6 | 1 | 2 | | | | | | | | | |
| AS | | | | 7 | 1 | 2 | | | | | | | | 11:24 | 4 |
| AS | 1213 | 11:31 | 3 | 8 | 1 | 0 | | | | | | | | | |
| AS | | | | 9 | 1 | 0 | | | | | | | | | |
| AS | | | | 10 | 1 | 0 | | | | | | | | | |
| AS | | | | 11 | 1 | 0 | | | | | | | | | |
| AS | | | | 12 | 0 | | | | | | | | | | |

| | | | | | | | | | | | | | | | |
|---|---|---|---|---|---|---|---|---|---|---|---|---|---|---|---|
| AS | | | | 13 | 0 | | | | | | | | | | |
| AS | | | | 14 | 0 | | | | | | | | | | |
| AS | | | | 15 | 0 | | | | | | | | | 11:38 | 7 |
| AS | 1280 | 11:39 | 2 | 8 | 1 | 0 | | | | | | | | | |
| AS | | | | 9 | 1 | 1 | 73,75 | 53,33 | 47,56 | 10x15 | 1 | 0 | 0,705 | | |
| AS | | | | 10 | 1 | 0 | | | | | | | | | |
| AS | | | | 11 | 1 | 1 | 81,28 | 51,27 | 47,06 | 19x18 | 1 | 0 | 2,211 | | |
| AS | | | | 12 | 1 | 0 | | | | | | | | | |
| AS | | | | 13 | 1 | 0 | | | | | | | | | |
| AS | | | | 14 | 1 | 0 | | | | | | | | | |
| AS | | | | 15 | 1 | 1 | 79,7 | 46,82 | 48,62 | 4x5 | 0 | 0 | 0,375 | 11:50 | 11 |
| AS | 1202 | 12:03 | 2 | 8 | 1 | 1 | 82,64 | 54,97 | 36,07 | 9x6 | 0 | 0 | 0,606 | | |
| AS | | | | 9 | 1 | 1 | 75,52 | 51,95 | 33,94 | 2x1 | 0 | 1 | 0,183 | | |
| AS | | | | 10 | 1 | 1 | 83,14 | 48,05 | 35,34 | 3x3 | 0 | 0 | 0,418 | | |
| AS | | | | 11 | 1 | 0 | | | | | | | | | |
| AS | | | | 12 | 1 | 0 | | | | | | | | | |
| AS | | | | 13 | 1 | 0 | | | | | | | | | |
| AS | | | | 14 | 1 | 0 | | | | | | | | | |
| AS | | | | 15 | 1 | 0 | | | | | | | | 12:12 | 9 |
| AS | 1270 | 12:13 | 2 | 8 | 1 | 1 | 78,05 | 53,29 | 41,2 | 3x4 | 0 | 1 | 0,215 | | |
| AS | | | | 9 | 1 | 0 | | | | | | | | | |
| AS | | | | 10 | 1 | 0 | | | | | | | | | |
| AS | | | | 11 | 1 | 0 | | | | | | | | | |
| AS | | | | 12 | 1 | 0 | | | | | | | | | |
| AS | | | | 13 | 1 | 0 | | | | | | | | | |
| AS | | | | 14 | 1 | 0 | | | | | | | | | |
| AS | | | | 15 | 1 | 0 | | | | | | | | 12:20 | 7 |
| AS | 1295 | 13:10 | 0 | 0 | 1 | 1 | 34,33 | 49,61 | 75,96 | 1x2 | 0 | 1 | 0,21 | | |
| AS | | | | 0 | 1 | 1 | 35,88 | 49,49 | 76,12 | 1x3 | 0 | 1 | 0,232 | | |
| AS | | | | 1 | 1 | 0 | | | | | | | | | |
| AS | | | | 2 | 1 | 0 | | | | | | | | | |
| AS | | | | 3 | 1 | 0 | | | | | | | | | |
| AS | | | | 4 | 1 | 0 | | | | | | | | | |
| AS | | | | 5 | 1 | 0 | | | | | | | | | |
| AS | | | | 6 | 1 | 0 | | | | | | | | | |
| AS | | | | 7 | 1 | 0 | | | | | | | | 13:18 | 8 |
| AS | 1236 | 13:19 | 0 | 0 | 1 | 1 | 73,48 | 52,67 | 81,94 | 3x2 | 0 | 1 | 0,207 | | |
| AS | | | | 1 | 1 | 0 | | | | | | | | | |
| AS | | | | 2 | 1 | 0 | | | | | | | | | |
| AS | | | | 3 | 1 | 0 | | | | | | | | | |
| AS | | | | 4 | 1 | 1 | 75,46 | 51,44 | 92,43 | 3x5 | 0 | 0 | 0,598 | | |
| AS | | | | 5 | 1 | 0 | | | | | | | | | |
| AS | | | | 6 | 1 | 1 | 73,3 | 41,12 | 91,53 | 2x1 | 0 | 1 | 0,2 | | |
| AS | | | | 7 | 1 | 0 | | | | | | | | 13:28 | 9 |
| AS | 1256 | 13:32 | 2 | 8 | 1 | 0 | | | | | | | | | |
| AS | | | | 9 | 1 | 0 | | | | | | | | | |
| AS | | | | 10 | 1 | 1 | 80,55 | 44,45 | 53,07 | 2x1 | 0 | 1 | 0,258 | | |
| AS | | | | 11 | 1 | 0 | | | | | | | | | |
| AS | | | | 12 | 1 | 0 | | | | | | | | | |
| AS | | | | 13 | 1 | 0 | | | | | | | | | |
| AS | | | | 14 | 1 | 0 | | | | | | | | | |
| AS | | | | 15 | 1 | 0 | | | | | | | | 13:37 | 5 |
| AS | 1282 | 13:38 | 1 | 0 | 1 | 0 | | | | | | | | | |
| AS | | | | 1 | 1 | 0 | | | | | | | | | |
| AS | | | | 2 | 1 | 0 | | | | | | | | | |
| AS | | | | 3 | 1 | 0 | | | | | | | | | |
| AS | | | | 4 | 1 | 0 | | | | | | | | | |
| AS | | | | 5 | 1 | 1 | 66,87 | 46,43 | 104,39 | 5x4 | 0 | 1 | 0,263 | | |
| AS | | | | 6 | 1 | 0 | | | | | | | | | |
| AS | | | | 7 | 1 | 1 | 57,33 | 45,81 | 105,86 | 7x6 | 1 | 0 | 0,444 | 13:46 | 8 |
| AS | 1279 | 13:47 | 1 | 0 | 1 | 0 | | | | | | | | | |
| AS | | | | 1 | 1 | 0 | | | | | | | | | |
| AS | | | | 2 | 1 | 0 | | | | | | | | | |
| AS | | | | 3 | 1 | 0 | | | | | | | | | |
| AS | | | | 4 | 1 | 0 | | | | | | | | | |
| AS | | | | 5 | 1 | 0 | | | | | | | | | |
| AS | | | | 6 | 1 | 1 | 66,89 | 34,08 | 96,97 | 3x1 | 0 | 0 | 0,554 | | |
| AS | | | | 7 | 1 | 1 | 60,66 | 39,13 | 93,69 | 28x25 | 0 | 0 | 1,973 | 13:56 | 9 |
| AS | 1271 | 14:21 | 2 | 8 | 1 | 1 | 45,12 | 56,29 | 32,78 | 2x1 | 0 | 1 | 0,149 | | |
| AS | | | | 8 | 1 | 1 | 47,64 | 57,32 | 34,01 | 4x2 | 0 | 0 | 0,715 | | |
| AS | | | | 9 | 1 | 1 | 54,83 | 53,59 | 33,19 | 2x1 | 0 | 1 | 0,121 | | |
| AS | | | | 10 | 1 | 0 | | | | | | | | | |
| AS | | | | 11 | 1 | 0 | | | | | | | | | |
| AS | | | | 12 | 1 | 0 | | | | | | | | | |
| AS | | | | 13 | 1 | 1 | 54,92 | 53,45 | 40,08 | 2x1 | 0 | 1 | 0,158 | | |
| AS | | | | 14 | 1 | 0 | | | | | | | | | |
| AS | | | | 15 | 1 | 0 | | | | | | | | 14:32 | 11 |
| AS | 1266 | 14:39 | 0 | 0 | 1 | 1 | 39,13 | 48,41 | 64,01 | 1x2 | 0 | 1 | 0,54 | | |
| AS | | | | 1 | 1 | 0 | | | | | | | | | |
| AS | | | | 2 | 1 | 0 | | | | | | | | | |
| AS | | | | 3 | 1 | 0 | | | | | | | | | |
| AS | | | | 4 | 0 | | | | | | | | | | |
| AS | | | | 5 | 0 | | | | | | | | | | |
| AS | | | | 6 | 0 | | | | | | | | | | |
| AS | | | | 7 | 0 | | | | | | | | | 14:43 | 4 |
| AS | 1291 | 14:43 | 0 | 0 | 1 | 0 | | | | | | | | | |
| AS | | | | 1 | 1 | 1 | 73,74 | 43,29 | 80,13 | 2x1 | 0 | 1 | 0,259 | | |
| AS | | | | 2 | 1 | 1 | 85,29 | 36,83 | 82,67 | 14x20 | 1 | 0 | 1,703 | | |
| AS | | | | 3 | 1 | 1 | 88,54 | 39,89 | 81,11 | 25x38 | 1 | 0 | 3,161 | | |
| AS | | | | 4 | 1 | 1 | 82,87 | 43,84 | 92,6 | 4x4 | 0 | 1 | 0,631 | | |
| AS | | | | 5 | 1 | 1 | 75,78 | 39,04 | 90,54 | 2x2 | 0 | 1 | 0,362 | | |
| AS | | | | 6 | 1 | 1 | 78,74 | 32,89 | 90,8 | 10x11 | 1 | 0 | 0,768 | | |
| AS | | | | 7 | 1 | 1 | 85,52 | 38,25 | 90,71 | 86x49 | 1 | 0 | 6,606 | 14:58 | 15 |
| AS | 1242 | 15:20 | 1 | 0 | 1 | 1 | 52,83 | 45,37 | 71,97 | 4x3 | 0 | 1 | 0,465 | | |
| AS | | | | 1 | 1 | 0 | | | | | | | | | |
| AS | | | | 2 | 1 | 0 | | | | | | | | | |
| AS | | | | 3 | 1 | 1 | 62,03 | 36,78 | 73,45 | 3x24 | 1 | 0 | 0,887 | | |
| AS | | | | 4 | 1 | 0 | | | | | | | | | |
| AS | | | | 5 | 1 | 0 | | | | | | | | | |
| AS | | | | 6 | 1 | 0 | | | | | | | | | |
| AS | | | | 7 | 1 | 1 | 59,88 | 35,08 | 81,81 | 3x6 | 1 | 0 | 0,465 | 15:27 | 7 |
| AS | 1262 | 15:28 | 2 | 8 | 1 | 0 | | | | | | | | | |
| AS | | | | 9 | 1 | 0 | | | | | | | | | |
| AS | | | | 10 | 1 | 0 | | | | | | | | | |
| AS | | | | 11 | 1 | 1 | 75,04 | 50,9 | 41,29 | 4x2 | 0 | 1 | 0,159 | | |
| AS | | | | 12 | 1 | 0 | | | | | | | | | |
| AS | | | | 13 | 1 | 0 | | | | | | | | | |
| AS | | | | 14 | 1 | 1 | 72,42 | 45,22 | 49,65 | 1x3 | 0 | 0 | 0,532 | | |
| AS | | | | 15 | 1 | 0 | | | | | | | | 15:35 | 7 |
| AS | 1237 | 15:43 | 0 | 0 | 1 | 0 | | | | | | | | | |
| AS | | | | 1 | 1 | 0 | | | | | | | | | |
| AS | | | | 2 | 1 | 0 | | | | | | | | | |
| AS | | | | 3 | 1 | 1 | 39,78 | 41,65 | 68,17 | 3x4 | 0 | 0 | 0,585 | | |
| AS | | | | 4 | 1 | 0 | | | | | | | | | |
| AS | | | | 5 | 1 | 0 | | | | | | | | | |
| AS | | | | 6 | 1 | 0 | | | | | | | | | |
| AS | | | | 7 | 1 | 1 | 44,19 | 38,15 | 75,79 | 3x1 | 0 | 0 | 0,378 | | |
| AS | | | | 7 | 1 | 1 | 42,1 | 40,87 | 81,12 | 3x1 | 0 | 1 | 0,254 | 15:53 | 10 |
| AS | 1225 | 8:36 | 0 | 0 | 1 | 1 | 42,92 | 50,51 | 84,2 | 6x5 | 0 | 0 | 0,788 | | |
| AS | | | | 1 | 1 | 1 | 50,13 | 44,03 | 80,59 | 11x13 | 0 | 0 | 1,094 | | |
| AS | | | | 1 | 1 | 1 | 49,92 | 43,48 | 83,87 | 4x4 | 0 | 0 | 0,463 | | |
| AS | | | | 2 | 1 | 0 | | | | | | | | | |
| AS | | | | 3 | 1 | 1 | 36,58 | 47,58 | 85,6 | 6x11 | 0 | 0 | 0,883 | | |
| AS | | | | 4 | 1 | 2 | | | | | | | | | |
| AS | | | | 5 | 1 | 2 | | | | | | | | | |
| AS | | | | 6 | 1 | 2 | | | | | | | | | |
| AS | | | | 7 | 1 | 2 | | | | | | | | 8:47 | |
| AS | 1286 | 8:47 | 3 | 8 | 1 | 0 | | | | | | | | | |
| AS | | | | 9 | 1 | 0 | | | | | | | | | |
| AS | | | | 10 | 1 | 0 | | | | | | | | | |
| AS | | | | 11 | 1 | 0 | | | | | | | | | |
| AS | | | | 12 | 1 | 0 | | | | | | | | | |
| AS | | | | 13 | 1 | 0 | | | | | | | | | |
| AS | | | | 14 | 1 | 0 | | | | | | | | | |
| AS | | | | 15 | 1 | 0 | | | | | | | | 8:52 | |
| AS | 1223 | 8:53 | 3 | 8 | 1 | 1 | 62,77 | 52,67 | 42,42 | 2x2 | 0 | 1 | 0,206 | | |
| AS | | | | 9 | 1 | 1 | 52,32 | 52,11 | 44,55 | 7x13 | 0 | 0 | 0,966 | | |
| AS | | | | 10 | 1 | 0 | | | | | | | | | |
| AS | | | | 11 | 1 | 1 | 63,05 | 47,96 | 44,63 | 4x11 | 1 | 0 | 1,274 | | |
| AS | | | | 12 | 1 | 0 | | | | | | | | | |
| AS | | | | 13 | 1 | 1 | 51,87 | 50,71 | 48,24 | 5x4 | 0 | 0 | 0,319 | | |
| AS | | | | 14 | 1 | 1 | 55,57 | 46,54 | 50,94 | 2x2 | 0 | 1 | 0,113 | | |
| AS | | | | 15 | 1 | 0 | | | | | | | | 9:04 | |
| AS | 1241 | 9:29 | 1 | 0 | 1 | 1 | 56,52 | 50,27 | 71,19 | 3x2 | 0 | 1 | 0,258 | | |
| AS | | | | 0 | 1 | 1 | 57,23 | 50,14 | 73,4 | 13x21 | 1 | 0 | 1,373 | | |
| AS | | | | 1 | 1 | 1 | 51,19 | 47,08 | 72,01 | 6x7 | 0 | 0 | 0,783 | | |
| AS | | | | 1 | 1 | 1 | 51,7 | 44,72 | 73,15 | 17x28 | 0 | 0 | 1,332 | | |
| AS | | | | 2 | 1 | 0 | | | | | | | | | |
| AS | | | | 3 | 1 | 1 | 63,66 | 45,32 | 70,2 | 1x2 | 0 | 1 | 0,292 | | |
| AS | | | | 3 | 1 | 1 | 62,03 | 41,6 | 72,5 | 8x7 | 0 | 0 | 0,54 | | |
| AS | | | | 3 | 1 | 1 | 63,12 | 43,77 | 73,15 | 4x9 | 0 | 0 | 0,732 | | |
| AS | | | | 4 | 1 | 2 | | | | | | | | | |
| AS | | | | 5 | 1 | 2 | | | | | | | | | |
| AS | | | | 6 | 1 | 2 | | | | | | | | | |
| AS | | | | 7 | 1 | 2 | | | | | | | | 9:40 | |
| AS | 1250 | 9:43 | 2 | 8 | 1 | 1 | 83,96 | 53,17 | 48,69 | 7x7 | 0 | 1 | 0,422 | | |
| AS | | | | 9 | 1 | 0 | | | | | | | | | |
| AS | | | | 10 | 1 | 0 | | | | | | | | | |
| AS | | | | 11 | 1 | 0 | | | | | | | | | |
| AS | | | | 12 | 1 | 0 | | | | | | | | | |

| | | | | | | | | | | | | | | |
|---|---|---|---|---|---|---|---|---|---|---|---|---|---|---|
| AS | | | | 13 | 1 | 0 | | | | | | | | |
| AS | | | | 14 | 1 | 0 | | | | | | | | |
| AS | | | | 15 | 1 | 0 | | | | | | | | 9:49 |
| AS | 1263 | 9:49 | 3 | 8 | 1 | 1 | 61,86 | 57,48 | 48,87 | 2x3 | 0 | 1 | 0,128 | |
| AS | | | | 8 | 1 | 1 | 56,64 | 58,77 | 49,94 | 2x2 | 0 | 1 | 0,196 | |
| AS | | | | 9 | 1 | 0 | | | | | | | | |
| AS | | | | 10 | 1 | 0 | | | | | | | | |
| AS | | | | 11 | 1 | 0 | | | | | | | | |
| AS | | | | 12 | 1 | 0 | | | | | | | | |
| AS | | | | 13 | 1 | 0 | | | | | | | | |
| AS | | | | 14 | 1 | 0 | | | | | | | | |
| AS | | | | 15 | 1 | 0 | | | | | | | | 9:56 |
| AS | 1297 | 11:08 | 3 | 8 | 1 | 0 | | | | | | | | |
| AS | | | | 9 | 1 | 0 | | | | | | | | |
| AS | | | | 10 | 1 | 0 | | | | | | | | |
| AS | | | | 11 | 1 | 1 | 64,64 | 52,53 | 26,55 | 3x2 | 0 | 0 | 0,546 | |
| AS | | | | 12 | 1 | 0 | | | | | | | | |
| AS | | | | 13 | 1 | 0 | | | | | | | | |
| AS | | | | 14 | 1 | 0 | | | | | | | | |
| AS | | | | 15 | 1 | 0 | | | | | | | | 11:15 |
| AS | 1230 | 11:16 | 1 | 0 | 1 | 1 | 61,87 | 56,35 | 92,01 | 2x5 | 0 | 1 | 0,343 | |
| AS | | | | 1 | 1 | 0 | | | | | | | | |
| AS | | | | 2 | 1 | 0 | | | | | | | | |
| AS | | | | 3 | 1 | 0 | | | | | | | | |
| AS | | | | 4 | 1 | 0 | | | | | | | | |
| AS | | | | 5 | 1 | 1 | 52,24 | 52,06 | 105,95 | 1x2 | 0 | 1 | 0,281 | |
| AS | | | | 6 | 1 | 0 | | | | | | | | |
| AS | | | | 7 | 1 | 0 | | | | | | | | 11:23 |
| AS | 1290 | 11:34 | 2 | 8 | 1 | 0 | | | | | | | | |
| AS | | | | 9 | 1 | 0 | | | | | | | | |
| AS | | | | 10 | 1 | 0 | | | | | | | | |
| AS | | | | 11 | 1 | 0 | | | | | | | | |
| AS | | | | 12 | 1 | 0 | | | | | | | | |
| AS | | | | 13 | 1 | 0 | | | | | | | | |
| AS | | | | 14 | 1 | 0 | | | | | | | | |
| AS | | | | 15 | 1 | 1 | 89,98 | 43,69 | 47,26 | 21x18 | 1 | 0 | 1,483 | 11:40 |
| AS | 1247 | 11:40 | 2 | 8 | 1 | 1 | 42,45 | 58,29 | 38,12 | 3x2 | 0 | 1 | 0,146 | |
| AS | | | | 9 | 1 | 1 | 48,84 | 52,58 | 37,46 | 1x2 | 0 | 1 | 0,251 | |
| AS | | | | 10 | 1 | 0 | | | | | | | | |
| AS | | | | 11 | 1 | 0 | | | | | | | | |
| AS | | | | 12 | 1 | 0 | | | | | | | | |
| AS | | | | 13 | 1 | 0 | | | | | | | | |
| AS | | | | 14 | 1 | 0 | | | | | | | | |
| AS | | | | 15 | 1 | 0 | | | | | | | | 11:45 |
| AS | 1257 | 11:46 | 0 | 0 | 1 | 0 | | | | | | | | |
| AS | | | | 1 | 1 | 0 | | | | | | | | |
| AS | | | | 2 | 1 | 0 | | | | | | | | |
| AS | | | | 3 | 1 | 0 | | | | | | | | |
| AS | | | | 4 | 1 | 0 | | | | | | | | |
| AS | | | | 5 | 1 | 1 | 78,64 | 37,65 | 73,9 | 3x2 | 0 | 1 | 0,154 | |
| AS | | | | 6 | 1 | 0 | | | | | | | | |
| AS | | | | 7 | 1 | 1 | 89,27 | 36,13 | 75,95 | 2x1 | 0 | 1 | 0,28 | 11:53 |
| AS | 1201 | 11:54 | 0 | 0 | 1 | 0 | | | | | | | | |
| AS | | | | 1 | 1 | 0 | | | | | | | | |
| AS | | | | 2 | 1 | 0 | | | | | | | | |
| AS | | | | 3 | 1 | 0 | | | | | | | | |
| AS | | | | 4 | 1 | 0 | | | | | | | | |
| AS | | | | 5 | 1 | 0 | | | | | | | | |
| AS | | | | 6 | 1 | 0 | | | | | | | | |
| AS | | | | 7 | 1 | 0 | | | | | | | | 12:00 |
| AS | 1303 | 12:00 | 3 | 8 | 1 | 1 | 67,41 | 48,36 | 40,38 | 2x1 | 0 | 1 | 0,108 | |
| AS | | | | 9 | 1 | 1 | 77,96 | 46,16 | 43,09 | 5x15 | 0 | 0 | 2,197 | |
| AS | | | | 10 | 1 | 0 | | | | | | | | |
| AS | | | | 11 | 1 | 1 | 66,33 | 45,26 | 41,86 | 13x15 | 1 | 0 | 2,059 | |
| AS | | | | 12 | 1 | 0 | | | | | | | | |
| AS | | | | 13 | 1 | 1 | 76,13 | 47,13 | 50,47 | 9x6 | 0 | 0 | 0,916 | |
| AS | | | | 13 | 1 | 1 | 75,37 | 43,02 | 46,37 | 14x8 | 0 | 0 | 0,82 | |
| AS | | | | 14 | 1 | 1 | 73,44 | 41,2 | 47,19 | 8x1 | 0 | 0 | 0,377 | |
| AS | | | | 15 | 1 | 1 | 68,24 | 42,92 | 45,96 | 19x29 | 1 | 0 | 1,982 | 12:16 |
| AS | 1275 | 14:05 | 2 | 8 | 1 | 0 | | | | | | | | |
| AS | | | | 9 | 1 | 1 | 72,76 | 52,2 | 49,2 | 4x2 | 0 | 1 | 0,247 | |
| AS | | | | 10 | 1 | 1 | 80,25 | 48,17 | 46,91 | 2x2 | 0 | 1 | 0,215 | |
| AS | | | | 11 | 1 | 0 | | | | | | | | |
| AS | | | | 12 | 1 | 0 | | | | | | | | |
| AS | | | | 13 | 1 | 0 | | | | | | | | |
| AS | | | | 14 | 1 | 0 | | | | | | | | |
| AS | | | | 15 | 1 | 0 | | | | | | | | 14:13 |
| AS | 1284 | 14:13 | 3 | 8 | 1 | 0 | | | | | | | | |
| AS | | | | 9 | 1 | 0 | | | | | | | | |
| AS | | | | 10 | 1 | 0 | | | | | | | | |
| AS | | | | 11 | 1 | 0 | | | | | | | | |
| AS | | | | 12 | 1 | 0 | | | | | | | | |
| AS | | | | 13 | 1 | 1 | 67,77 | 56,55 | 58,17 | 28x21 | 1 | 0 | 2,468 | |
| AS | | | | 14 | 1 | 0 | | | | | | | | |
| AS | | | | 15 | 1 | 0 | | | | | | | | 14:20 |
| AS | 1206 | 14:20 | 3 | 8 | 1 | 0 | | | | | | | | |
| AS | | | | 9 | 1 | 0 | | | | | | | | |
| AS | | | | 10 | 1 | 0 | | | | | | | | |
| AS | | | | 11 | 1 | 0 | | | | | | | | |
| AS | | | | 12 | 1 | 0 | | | | | | | | |
| AS | | | | 13 | 1 | 0 | | | | | | | | |
| AS | | | | 14 | 1 | 0 | | | | | | | | |
| AS | | | | 15 | 1 | 0 | | | | | | | | 14:25 |
| AS | 1302 | 14:32 | 0 | 0 | 1 | 1 | 80 | 50,38 | 76,86 | 6x6 | 0 | 1 | 0,42 | |
| AS | | | | 1 | 1 | 0 | | | | | | | | |
| AS | | | | 2 | 1 | 0 | | | | | | | | |
| AS | | | | 3 | 1 | 0 | | | | | | | | |
| AS | | | | 4 | 1 | 0 | | | | | | | | |
| AS | | | | 5 | 1 | 0 | | | | | | | | |
| AS | | | | 6 | 1 | 1 | 77,42 | 37,91 | 87,6 | 3x1 | 0 | 1 | 0,21 | |
| AS | | | | 7 | 1 | 0 | | | | | | | | 14:41 |
| AS | 1289 | 14:43 | 0 | 0 | 1 | 1 | 46,21 | 51,77 | 71,45 | 2x2 | 0 | 1 | 0,394 | |
| AS | | | | 1 | 1 | 0 | | | | | | | | |
| AS | | | | 2 | 1 | 0 | | | | | | | | |
| AS | | | | 3 | 1 | 0 | | | | | | | | |
| AS | | | | 4 | 1 | 0 | | | | | | | | |
| AS | | | | 5 | 1 | 0 | | | | | | | | |
| AS | | | | 6 | 1 | 0 | | | | | | | | |
| AS | | | | 7 | 1 | 1 | 42,71 | 45,71 | 83,91 | 2x2 | 0 | 1 | 0,346 | 14:52 |
| AS | 1244 | 14:52 | 0 | 0 | 1 | 0 | | | | | | | | |
| AS | | | | 1 | 1 | 0 | | | | | | | | |
| AS | | | | 2 | 1 | 0 | | | | | | | | |
| AS | | | | 3 | 1 | 1 | 37,45 | 44,92 | 68,53 | 1x3 | 0 | 1 | 0,406 | |
| AS | | | | 3 | 1 | 1 | 37,31 | 47,51 | 69,68 | 13x18 | 0 | 0 | 1,251 | |
| AS | | | | 4 | 1 | 0 | | | | | | | | |
| AS | | | | 5 | 1 | 1 | 49,36 | 45,64 | 78,94 | 4x2 | 0 | 1 | 0,137 | |
| AS | | | | 6 | 1 | 1 | 45,47 | 39,36 | 78,04 | 2x5 | 0 | 0 | 0,347 | |
| AS | | | | 7 | 1 | 0 | | | | | | | | 15:01 |
| AS | 1264 | 15:09 | 0 | 0 | 1 | 1 | 76,45 | 45,22 | 77,6 | 1x2 | 0 | 1 | 0,193 | |
| AS | | | | 0 | 1 | 1 | 77,41 | 45,39 | 79,97 | 1x2 | 0 | 1 | 0,165 | |
| AS | | | | 0 | 1 | 1 | 81,48 | 45,81 | 80,3 | 2x1 | 0 | 1 | 0,208 | |
| AS | | | | 1 | 1 | 0 | | | | | | | | |
| AS | | | | 2 | 1 | 0 | | | | | | | | |
| AS | | | | 3 | 1 | 0 | | | | | | | | |
| AS | | | | 4 | 1 | 1 | 79,97 | 43,28 | 91,54 | 3x5 | 0 | 0 | 0,329 | |
| AS | | | | 5 | 1 | 0 | | | | | | | | |
| AS | | | | 6 | 1 | 0 | | | | | | | | |
| AS | | | | 7 | 1 | 0 | | | | | | | | 15:19 |
| AS | 1212 | 15:19 | 2 | 8 | 1 | 0 | | | | | | | | |
| AS | | | | 9 | 1 | 1 | 52,34 | 53,09 | 44,82 | 2x2 | 0 | 1 | 0,094 | |
| AS | | | | 10 | 1 | 0 | | | | | | | | |
| AS | | | | 11 | 1 | 0 | | | | | | | | |
| AS | | | | 12 | 1 | 0 | | | | | | | | |
| AS | | | | 13 | 1 | 0 | | | | | | | | |
| AS | | | | 14 | 1 | 0 | | | | | | | | |
| AS | | | | 15 | 1 | 0 | | | | | | | | 15:25 |
| AS | 1227 | 15:26 | 2 | 8 | 1 | 2 | | | | | | | | |
| AS | | | | 9 | 1 | 2 | | | | | | | | |
| AS | | | | 10 | 1 | 2 | | | | | | | | |
| AS | | | | 11 | 1 | 2 | | | | | | | | |
| AS | | | | 12 | 1 | 2 | | | | | | | | |
| AS | | | | 13 | 1 | 2 | | | | | | | | |
| AS | | | | 14 | 1 | 2 | | | | | | | | |
| AS | | | | 15 | 1 | 2 | | | | | | | | 15:27 |
| AS | 1234 | 15:27 | 3 | 8 | 1 | 0 | | | | | | | | |
| AS | | | | 9 | 1 | 0 | | | | | | | | |
| AS | | | | 10 | 1 | 0 | | | | | | | | |
| AS | | | | 11 | 1 | 0 | | | | | | | | |
| AS | | | | 12 | 1 | 0 | | | | | | | | |
| AS | | | | 13 | 1 | 0 | | | | | | | | |
| AS | | | | 14 | 1 | 0 | | | | | | | | |
| AS | | | | 15 | 1 | 0 | | | | | | | | 15:33 |
| AS | 1267 | 15:33 | 3 | 8 | 1 | 1 | 62,04 | 54,83 | 46,44 | 5x3 | 0 | 1 | 0,351 | |
| AS | | | | 9 | 1 | 0 | | | | | | | | |
| AS | | | | 10 | 1 | 0 | | | | | | | | |
| AS | | | | 11 | 1 | 0 | | | | | | | | |
| AS | | | | 12 | 1 | 0 | | | | | | | | |
| AS | | | | 13 | 1 | 0 | | | | | | | | |
| AS | | | | 14 | 1 | 0 | | | | | | | | |
| AS | | | | 15 | 1 | 0 | | | | | | | | 15:38 |

| | | | | | | | | | | | | | | |
|---|---|---|---|---|---|---|---|---|---|---|---|---|---|---|
| AS | 1255 | 15:39 | 3 | 8 | 1 | 0 | | | | | | | | |
| AS | | | | 9 | 1 | 0 | | | | | | | | |
| AS | | | | 10 | 1 | 0 | | | | | | | | |
| AS | | | | 11 | 1 | 0 | | | | | | | | |
| AS | | | | 12 | 0 | | | | | | | | | |
| AS | | | | 13 | 0 | | | | | | | | | |
| AS | | | | 14 | 0 | | | | | | | | | |
| AS | | | | 15 | 0 | | | | | | | | | 15:41 |
| AS | 1208 | 15:41 | 1 | 0 | 1 | 0 | | | | | | | | |
| AS | | | | 1 | 1 | 0 | | | | | | | | |
| AS | | | | 2 | 1 | 0 | | | | | | | | |
| AS | | | | 3 | 1 | 0 | | | | | | | | |
| AS | | | | 4 | 1 | 0 | | | | | | | | |
| AS | | | | 5 | 1 | 0 | | | | | | | | |
| AS | | | | 6 | 1 | 0 | | | | | | | | |
| AS | | | | 7 | 1 | 0 | | | | | | | | 15:46 |
| AS | 1273 | 15:47 | 3 | 8 | 1 | 0 | | | | | | | | |
| AS | | | | 9 | 1 | 0 | | | | | | | | |
| AS | | | | 10 | 1 | 0 | | | | | | | | |
| AS | | | | 11 | 1 | 1 | 66,76 | 47,47 | 50,71 | 15x7 | 1 | 0 | 0,876 | |
| AS | | | | 12 | 1 | 2 | | | | | | | | |
| AS | | | | 13 | 1 | 2 | | | | | | | | |
| AS | | | | 14 | 1 | 2 | | | | | | | | |
| AS | | | | 15 | 1 | 2 | | | | | | | | 15:51 |
| AS | 1298 | 15:52 | 2 | 8 | 0 | | | | | | | | | |
| AS | | | | 9 | 0 | | | | | | | | | |
| AS | | | | 10 | 0 | | | | | | | | | |
| AS | | | | 11 | 0 | | | | | | | | | |
| AS | | | | 12 | 0 | | | | | | | | | |
| AS | | | | 13 | 0 | | | | | | | | | |
| AS | | | | 14 | 0 | | | | | | | | | |
| AS | | | | 15 | 0 | | | | | | | | | 15:53 |
