# Supplementary material for: Development of a scoring method to visually score cortical interruptions on high-resolution peripheral quantitative computed tomography in rheumatoid arthritis and healthy controls
Source: PLoS One. 2018 Jul 9;13(7):e0200331. doi: 10.1371/journal.pone.0200331 (PMC6037386; doi:10.1371/journal.pone.0200331)
Supplement: S3 File — (PDF) [file pone.0200331.s003.pdf]

Reader initials	studyID	start time	joint (0=MCP2, 1= MCP3, 2=PIP2, 3=PIP3)	surface (0= palmar PB, 1=ulnar PB, 2=d	evaluable (0=no 1=yes)	discontinuity (0=no 1=yes 2=total destruction)	x value	y value	z value	#SLICES i	adjacent trabecular dist	parallel structure (0=no	maximal diameter	end time	total time	Remarks
MP	1246	17:37	3	8	1	0										
				9	1	0										
				10	1	0										
				11	1	0										
				12	1	0										
				13	1	1	51,71	56,8	36,92	9x6	1	0	1,317			
				14	1	1	57,81	50,11	35,94	2x2	0	0	0,17			
				15	1	0								17:45		
MP	1294	17:46	1	0	0											
				1	1	0										
				2	0											
				3	1	0										
				4	1	0										
				5	1	0										
				6	1	0										
				7	1	0								17:51		
MP	1200	17:53	0	0	1	1	83,16	46,3	94,5	3X4	0	1	0,593			
				0	1	1	84,79	46,72	94,42	2x2	0	1	0,408			
				0	1	1	80,18	45,99	94,82	3x3	0	1	0,2			
				0	1	1	82,75	45,37	98,02	7x10	1	0	1,167			
				1	1	1	75,79	40,75	96,96	5x9	0	0	0,665			
				1	1	1	75,38	41,72	96,06	4x3	0	0	0,45			
				1	1	1	76,27	40,38	95,4	2x1	0	0	0,205			
				1	1	1	75,36	43,14	95,97	2x2	0	0	0,153			
				2	1	1	86,74	36,48	95,64	7x5	0	0	1,625			
				3	1	1	90,15	43,89	95,97	15x19	0	0	1,924			
				3	1	1	90,38	41,32	93,43	4x3	0	1	0,623			
				4	1	1	86,07	40,14	105,65	7x7	0	1	1,265			
				5	1	1	78,16	35,75	102,04	32x26	0	0	3,863			
				5	1	1	79,07	36,56	103,76	3x3	0	1	0,491			
				6	1	1	84,86	29,77	108,19	10x8	0	0	1,192			
				6	1	1	81,31	29,38	102,78	4x18	1	0	1,554			
				7	1	1	87,52	33,58	100,07	22x7	1	0	1,729			
				7	1	1	89,38	35,59	106,63	35x17	1	0	2,536	18:31		
MP	1264	15:47	0	0	1	1	76,39	45,21	77,68	3x3	0	1	0,241			
				0	1	1	77,33	45,49	79,97	2x2	0	0	0,268			
				1	1	0										
				2	1	0										
				3	1	0										
				4	1	1	80,17	43,39	90,96	8x8	0	0	1,507			
				5	1	0										
				6	1	1	80,51	32,1	89,73	4x4	0	0	0,705			
				7	1	0								15:58		
MP	1255	15:59	3	8	1	1	66,58	58,93	31,34	5x2	0	0	0,413			
				9	1	0										
				10	1	0										
				11	1	0										
				12	0											
				13	0											
				14	0											
				15	0									16:02		
MP	1273	16:20		8	1	1	61,54	54,36	51,21	7X6	0	1	0,984			
				9	1	1	57,2	53,8	47,92	2X2	0	1	0,135			
				10	1	1	66,71	47,32	50,88	9X6	1	0	0,784			
				11	1	0										
				12	1	0										
				13	1	1	55,69	47,55	54,98	4X6	1	0	0,471			
				14	1	0										
				15	1	0								16:32		
MP	1237	16:35	0	0	1	0										
				1	1	0										
				2	1	0										
				3	1	1	39,65	41,66	68,41	5X5	0	0	1,02			
				4	1	0										
				5	1	0										
				6	1	0										
				7	1	1	44,26	38,23	75,87	5X5	1	0	0,434			
				7	1	1	39,66	44,27	75,55	4X3	0	0	0,25	0,697222		
MP	1212	0,6986111	2	8	1	1	48,32	54,96	48,35	4X5	0	1	0,378			
				8	1	1	43,31	54,86	47,04	2X3	0	1	0,19			
				9	1	0										
				10	1	0										
				11	1	0										
				12	1	0										
				13	1	0										
				14	1	0										
				15	1	0								0,702778		
MP	1210	0,7034722	3	8	1	0										
				9	1	0										
				10	1	0										
				11	1	0										
				12	1	0										
				13	1	1	71,25	55,16	48,84	14X9	1	0	1,033	0,707639		
				14	1	0										
				15	1	0										
MP	1284	0,7097222	3	8	1	0										
				9	1	0										
				10	1	0										
				11	1	0										
				12	1	0										
				13	1	1	68,13	55,72	60,14	3x2	0	0	0,339			
				13	1	1	67,84	56,46	58,25	27x8	1	0	1,268	0,716667		
				14	1	0										
				15	1	1	57,38	54,53	57,1	10x3	0	0	0,718			
MP	1249	0,7173611	3	8	1	1	63,37	54,84	40,75	2x2	0	1	0,598			
				9	1	0										
				10	1	0										
				11	1	0										
				12	1	0										
				13	1	0										
				14	1	0										
				15	1	1	65,16	50,9	45,59	19x11	1	0	1,228	17:19		
MP	1254	17:23	1	0	1	1	62,81	50,77	87,14	3x4	0	1	0,49			
				1	1	1	50,92	49,62	88,78	2x2	0	1	0,344			
				2	1	1	67,75	46,97	91,07	9x10	1	0	0,964			
				3	1	0										
				4	1	1	62,59	49,39	101,07	8x5	0	1	1,011			
				5	1	0										
				6	1	0										
				7	1	1	65,01	45,42	101,16	14x8	1	0	1,39			
				7	1	1	61,98	43,48	98,45	66x26	1	0	7,883	17:40		
MP	1230	17:42	1	0	1	1	58,79	57,17	91,52	4X3	0	1	0,531			
				0	1	1	61,95	56,6	91,93	6x6	0	1	0,616			
				1	0											
				2	0											
				3	1	0										
				4	1	0										
				5	1	1	54,07	51,93	105,87	3x3	0	1	0,538			
				6	1	0										
				7	1	0								17:50		
MP	1208	17:52	1	0	1	0										
				1	1	0										
				2	1	0										
				3	1	0										
				4	1	0										
				5	1	0										
				6	1	0										
				7	1	0								17:57		
MP	1275	17:59	2	8	1	1	58,22	55,18	45,51	7x7	0	0	0,422			
				8	1	1	62,16	53,94	46,25	5x5	0	0	0,597			
				8	1	1	53,26	56,01	47,32	2x3	0	0	0,368			
				9	1	1	51,41	51,08	46,99	3x4	0	0	0,509			
				10	1	0										
				11	1	0										
				12	1	0										
				13	1	0										
				14	1	1	56,37	46,62	52,81	3x5	0	1	0,291			
				15	1	0										
MP	1302	9:30	0	0	1	1	83,87	50,14	73,41	6X4	0	1	0,517			
				0	1	1	79,91	50,65	76,86	6x6	0	1	0,581			
				0	1	1	77,48	51,16	76,77	2x2	0	1	0,302			
				1	1	0										
				2	1	0										
				3	1	0										

				4	1	1	81,31	48,39	89,4	4x3	0	0	0,628		
				5	1	0									
				6	1	1	77,28	37,67	87,68	3x3	0	1	0,372		
				7	1	0								9:41	
MP	1239	9:42		8	1	0									
				9	1	0									
				10	1	0									
				11	1	0									
				12	1	0									
				13	1	1	35,93	55,09	39,54	5X6	1	0	0,827		
				14	1	0									
				15	1	0								9:47	
MP	1223	9:48		8	1	1	55,74	56,09	40,2	2X2	0	1	0,237		
				9	1	1	52,47	52,28	44,71	5x14	1	0	1,08		
				10	1	1	56,56	46,93	42,58	3x3	0	1	0,295		
				10	1	1	56,95	46,86	45,37	6x4	1	0	0,615		
				11	1	1	63,36	47,62	44,63	4x11	1	0	1,415		
				12	1	0									
				13	1	0									
				14	1	1	54,89	47,02	50,29	2x2	0	1	0,185		
				14	1	1	54,81	47,08	49,47	4x5	0	0	0,326		
				14	1	1	53,11	48,56	48,89	5x5	0	0	0,581		
				15	1	0								10:05	
MP	1262	10:18		8	1	0									
				9	1	0									
				10	1	0									
				11	1	1	75,53	50,68	42,44	6x3	0	0	0,423		
				12	1	0									
				13	1	0									
				14	1	0									
				15	1	0								10:22	
MP	1291	10:23	0	0	1	0									
				1	1	1	73,68	43,23	80,13	3X4	0	1	0,663		
				2	1	0									
				3	1	1	87,91	38,95	81,77	24X31	1	0	2,931		
				4	1	1	83,06	43,99	92,51	6X5	0	0	0,708		
				4	1	1	85,16	44,1	90,3	8X5	0	0	0,791		
				5	1	0									
				6	1	1	78,81	32,8	90,63	11x11	1	0	0,886		
				6	1	1	78,83	33	89,23	9x6	1	0	1,012		
				7	1	1	83,81	37,45	86,61	28x16	1	0	1,884		
					1	1	85,77	38,23	90,71	58x32	1	0	3,085	10:45	
MP	1287	16:41	1	0	1	1	63,93	49,25	68,21	3x3	0	1	0,256		
				1	1	0									
				2	1	0									
				3	1	0									
				4	1	1	64,1	47,28	82,4	4x5	0	0	0,52		
				5	1	0									
				6	1	0									
				7	1	1	65,32	41	82,48	9x7	1	0	1,153	16:49	
MP	1257	16:50	0	0	1	0									
				1	1	0									
				2	1	0									
				3	1	0									
				4	1	0									
				5	1	0									
				6	1	0									
				7	1	0								16:56	
MP	1297	16:57	3	8	1	0									
				9	1	0									
				10	1	0									
				11	1	1	64,07	49,97	29,33	3X3	0	0	0,41		
				12	1	0									
				13	1	0									
				14	1	0									
				15	1	1	64,71	49,12	37,78	2x2	0	0	0,386	17:04	
MP	1298	17:05		8	0										
				9	0										
				10	0										
				11	0										
				12	0										
				13	0										
				14	0										
				15	0									17:06	
MP	1247	17:07	2	8	1	1	42,38	58,41	38,12	3X3	0	1	0,504		
				8	1	1	45,14	58,17	38,28	3X3	0	0	0,379		
				8	1	1	47,2	57,85	38,94	3X3	0	0	0,275		
				9	1	1	48,99	53,81	37,46	2X2	0	0	0,231		
				10	1	0									
				11	1	0									
				12	1	0									
				13	1	0									
				14	1	0									
				15	1	0								17:16	
MP	1253	17:17	1	0	1	1	58,42	48,6	95,81	2X3	0	1	0,283		
				1	1	0									
				2	1	0									
				3	1	0									
				4	1	0									
				5	1	0									
				6	1	0									
				7	1	0							17:23		
MP	1252	17:24	2	8	1	1	81,72	56,02	37,9	3X3	0	0	0,39		
				8	1	1	78,54	56,89	39,54	3X4	0	1	0,449		
				8	1	1	72,88	57,75	39,54	2X2	0	0	0,248		
				9	1	0									
				10	1	0									
				11	1	1	83,64	53,72	37,49	3X3	0	0	0,278		
				11	1	1	82,33	51,08	38,4	3X3	0	0	0,342		
				12	1	0									
				13	1	1	72,89	56,58	45,86	4X3	1	0	0,46		
				14	1	1	79,61	48,38	44,71	4X3	0	0	0,373		
				15	1	1	81,44	51,14	42,58	2X2	0	0	0,28	17:39	
MP	1227	9:45	2	8	1	2									
				9	1	2									
				10	1	2									
				11	1	2									
				12	1	2									
				13	1	2									
				14	1	2									
				15	1	2									9:46
MP	1268	9:48	0	0	1	1	82,51	49,47	87,63	8X6	0	0	0,903		
				0	1	1	78,85	48,84	88,86	8X8	0	0	0,68		
				1	1	0									
				2	1	0									
				3	1	0									
				4	1	0									
				5	1	1	73,16	38,87	96,24	2X2	0	0	0,195		
				5	1	1	74,3	42,33	97,64	4X2	0	0	0,465		
				6	1	1	79,14	36,09	97,96	2X3	0	0	0,333		
				7	1	0								9:59	
MP	1202	10:01	2	8	1	1	82,61	55,01	35,99	6X5	0	0	0,513		
				8	1	1	79,87	55,71	36,4	3X3	0	1	0,336		
				9	1	0									
				10	1	1	83,6	48,25	34,02	2X2	0	0	0,597		
				11	1	1	85,99	53,18	31,65	7X5	0	1	0,419		
				12	1	0									
				13	1	0									
				14	1	0									
				15	1	0								10:09	
MP	1265	10:11	2	8	1	0									
				9	1	0									
				10	1	0									
				11	1	0									
				12	1	0									
				13	1	0									
				14	1	0									
				15	1	0								10:14	
MP	1204	10:15	1	0	1	1	60,21	53,6	92,16	3X3	0	1	0,262		
				0	1	1	63,03	53	92,57	3X4	0	1	0,38		
				0	1	1	61,83	55,76	93,3	5X5	0	1	0,632		
				0	1	1	63,03	53,9	95,44	19X24	1	0	2,35		
				1	1	0									
				2	1	0									

				3	1	0								
				4	1	1	60,86	53,75	106,75	2X2	0	0	0,388	
				4	1	1	65,86	53,09	105,19	6X7	0	0	0,806	
				5	1	1	66,75	51,23	104,62	14X16	1	0	1,514	
				6	1	0								
				7	1	1	54,7	48,52	106,59	11X11	1	0	0,903	
				7	1	1	56,26	45,49	105,03	11X10	1	0	1,383	
				7	1	1	56,85	43,91	100,44	4X10	0	0	0,916	10:36
MP	1272	9:25	1	0	1	1	62,34	51,76	85,41	3X3	0	0	0,388	
				0	1	1	66,59	52,75	87,46	6X5	0	1	0,513	
				0	1	1	64,86	52,45	88,77	2X2	0	1	0,234	
				1	1	1	72,07	49,32	89,75	2X2	0	0	0,378	
				2	1	1	62,35	42,32	89,26	6X5	0	1	0,639	
				3	1	1	58,06	49,68	84,75	5X5	0	0	0,72	
				3	1	1	58,43	47,06	90,66	15X21	1	0	1,514	
				4	1	1	63,32	51,59	101,97	9X6	0	0	1,225	
				5	1	1	69,86	46,89	99,02	3X3	0	1	0,272	
				6	1	0								
				7	1	1	60,38	45,47	95,66	9X5	1	0	0,837	
				7	1	1	61,06	44,95	97,38	19X12	1	0	1,792	9:47
MP	1220	9:52	3	8	1	1	62,69	51,75	40,82	7X7	0	0	1,451	
				9	1	0								
				10	1	0								
				11	1	0								
				12	1	0								
				13	1	0								
				14	1	1	68,84	44,08	46,81	7X7	0	0	0,446	
				14	1	1	62,09	41,66	44,84	4X4	0	0	0,308	
				15	1	0								10:01
MP	1300	10:12	3	8	1	1	57,57	51,76	42,11	6X5	0	1	0,458	
				8	1	1	54,57	53,41	43,01	5X4	0	1	0,489	
				9	1	0								
				10	1	0								
				11	1	0								
				12	1	0								
				13	1	0								
				14	1	0								
				15	1	0								10:21
MP	1243	10:24	0	0	1	1	42,27	51,21	74,96	3X3	0	1	0,334	
				0	1	1	46,09	49,96	75,7	3X4	0	1	0,463	
				1	1	0								
				2	1	0								
				3	1	0								
				4	1	1	44,29	48,75	87,35	8X7	0	1	0,522	
				5	1	0								
				6	1	0								
				7	1	0								10:30
MP	1274	10:32	3	8	1	0								
				9	1	1	67,27	50,34	53,82	2X3	0	0	0,386	
				10	1	0								
				11	1	0								
				12	1	0								
				13	1	0								
				14	1	0								
				15	1	0								10:36
MP	1301	10:38	2	8	1	1	79,05	58,32	41,73	2X2	0	1	0,303	
				8	1	1	79,5	57,85	42,88	3X3	0	0	0,445	
				9	1	0								
				10	1	0								
				11	1	0								
				12	1	0								
				13	1	0								
				14	1	1	78,79	49,87	44,6	2X2	0	0	0,286	
				15	1	0								
MP	1289	0,5986111	0	0	1	1	46,27	51,89	71,53	6x7	0	1	0,466	
				1	1	0								
				2	1	0								
				3	1	0								
				4	1	1	46,65	51,06	85,23	2x2	0	1	0,35	
				5	1	0								
				6	1	0								
				7	1	0								14:31
MP	1250	14:32	2	8	1	1	84,1	53,18	48,69	6X6	1	0	0,484	
				9	1	0								
				10	1	0								
				11	1	0								
				12	1	0								
				13	1	0								
				14	1	0								
				15	1	0								14:37
MP	1270	14:38	2	8	1	1	77,99	53,23	41,2	3X3	0	1	0,197	
				9	1	1	75,77	49,98	41,77	5X4	0	0	0,694	
				9	1	1	76,42	47,42	42,51	11X25	1	0	2,055	
				10	1	0								
				11	1	0								
				12	1	0								
				13	1	0								
				14	1	0								
				15	1	0								14:50
MP	1303	14:56	3	8	1	1	70,23	49,88	44,07	3X2	0	0	0,194	
				8	1	1	72,48	50,07	44,32	4X3	0	0	0,498	
				9	1	0								
				10	1	1	70,13	42,53	43	15X16	1	0	1,325	
				10	1	1	67,96	43,46	42,1	16X8	1	0	1,826	
				11	1	1	66,32	47,02	42,59	4X5	1	0	0,683	
				11	1	1	66,88	48,22	43,66	7X10	1	0	0,51	
				12	0									
				13	1	1	75,85	44,4	46,61	6X6	0	0	0,517	
				13	1	1	75,57	43,3	46,45	5X3	0	0	0,45	
				14	0									
				15	1	1	68,04	42,92	46,28	22X28	1	0	1,948	15:13
MP	1258	17:44	1	0	1	1	58,68	55,46	93,6	3X3	0	1	0,25	
				1	1	0								
				2	1	1	60,17	44,94	93,85	3X3	0	1	0,321	
				3	1	0								
				4	1	0								
				5	1	0								
				6	1	0								
				7	1	0								17:50
MP	1240	17:51	1	0	1	1	68,32	53,31	82,28	5X5	0	1	0,462	
				1	1	0								
				2	1	1	70,53	46,85	80,72	3X2	0	0	0,176	
				3	1	0								
				4	1	0								
				5	1	0								
				6	1	0								
				7	1	1	62,05	47,72	95,56	10X8	1	0	1,363	18:00
MP	1288	18:01	0	0	1	1	78,69	52,4	88	3X7	0	1	0,459	
				1	1	0								
				2	1	0								
				3	1	0								
				4	1	1	80,09	50,58	99,72	3X2	0	0	0,31	
				5	1	0								
				6	1	1	76,2	39,54	97,51	5X5	0	1	0,44	
				6	1	1	77,49	39,66	97,18	3X3	0	0	0,295	
				7	1	1	83,89	46,27	100,05	3X2	0	0	0,232	18:13
MP	1260	11:27	3	8	1	1	64,48	55,23	24,34	2X2	0	1	0,152	
				8	1	1	66,6	55,64	26,56	4X3	0	0	0,465	
				9	1	0								
				10	1	1	64,9	47,08	24,26	3X2	0	1	0,13	
				11	1	0								
				12	1	0								
				13	1	0								
				14	1	0								
				15	1	0								11:33
MP	1201	11:36	0	0	1	1	51,26	53,26	81,98	6X3	0	0	0,338	
				0	1	1	44,78	55,98	85,91	3X3	0	1	0,37	
				1	1	0								
				2	1	0								
				3	1	1	39,32	48,44	87,55	2X3	0	1	0,14	
				4	1	0								
				5	1	1	51,05	51,31	97,8	4X3	0	1	0,509	
				6	1	1	46,72	44,6	96,82	2X5	0	1	0,362	
				7	1	0								11:51

MP	1299	11:53	0	0	1	1	44,88	41,82	72,26	4X4	0	1	0,457	
				0	1	1	46,68	41,38	72,35	4X4	0	1	0,397	
				1	1	0								
				2	1	0								
				3	1	0								
				4	0									
				5	0									
				6	0									
				7	0									11:57
MP	1266	11:58	0	0	1	1	38,93	48,67	63,92	5X5	0	1	0,592	
				0	1	1	41,81	48,47	64,66	4X3	0	1	0,375	
				1	1	0								
				2	1	0								
				3	1	0								
				4	0									
				5	0									
				6	0									
				7	0									12:03
MP	1293	12:04	1	0	1	1	65,58	54,32	83,28	4X5	0	1	0,524	
				0	1	1	62,25	53,49	83,77	5X8	0	1	0,479	
				0	1	1	58,28	53,79	83,94	8X8	0	1	0,999	
				1	1	0								
				2	1	1	59,32	44,46	87,14	13X20	1	0	1,091	
				3	1	0								
				4	1	1	60,57	50,2	96,15	11X5	0	1	0,528	
				5	1	1	67,87	51,52	95,58	4X4	0	0	0,529	
				5	1	1	68,4	47,39	96,07	4X3	0	1	0,42	
				6	1	1	65,41	40,63	91,64	15X15	1	0	1,158	
				6	1	1	64,44	41,04	90,82	8X7	1	0	0,596	
				6	1	1	70,61	43,33	91,07	10X21	1	0	2,175	
				7	1	1	60,27	43,19	94,76	15X13	1	0	1,18	
				7	1	1	60,6	43,35	92,55	25X15	1	0	1,875	12:27
MP	1290	16:49	2	8	0									
				9	0									
				10	0									
				11	0									
				12	1	0								
				13	1	0								
				14	1	0								
				15	1	1	89,23	43,88	46,44	21X17	1	0	1,471	16:54
MP	1295	17:00	0	0	1	1	34,38	49,83	75,96	3X3	0	1	0,218	
				0	1	1	35,88	49,62	76,12	4X3	0	1	0,337	
				1	1	0								
				2	1	1	34,22	40,43	76,78	3X3	0	0	0,503	
				3	1	0								
				4	1	1	33,9	48,11	87,11	3X2	0	0	0,319	
				4	1	1	36,23	48,04	87,11	3X3	0	1	0,382	
				5	1	0								
				6	1	1	43,89	36,13	87,44	5x5	0	0	0,6	
				6	1	1	38,7	36,22	85,47	2x2	0	1	0,344	
				7	1	0								17:16
MP	1221	17:19	1	0	1	1	69,64	35,81	80,64	4X2	0	1	0,325	
				1	1	0								
				2	1	0								
				3	1	0								
				4	1	1	68,88	35,11	91,55	9X5	0	1	0,476	
				5	1	0								
				6	1	0								
				7	1	1	69,79	28,34	92,29	6X10	1	0	0,765	17:28
MP	1238	14:09	2	8	1	0								
				9	1	0								
				10	1	0								
				11	1	0								
				12	1	0								
				13	1	0								
				14	1	0								
				15	1	0								14:13
MP	1214	14:14	0	0	1	1	79,55	42,15	72,06	4X3	0	1	0,241	
				0	1	1	78,53	42,11	73,13	5X4	0	1	0,382	
				1	1	0								
				2	1	0								
				3	1	0								
				4	1	1	79,73	39,11	84,2	5X5	0	1	0,348	
				5	1	1	70,08	35,57	86,25	2X2	0	1	0,215	
				6	1	0								
				7	1	0								14:23
MP	1286	14:24	3	8	1	1	65,13	57,94	40,95	5X4	0	1	0,41	
				9	1	0								
				10	1	0								
				11	1	0								
				12	1	0								
				13	1	0								
				14	1	0								
				15	1	0								14:27
MP	1236	14:29	0	0	1	1	71,28	51,93	78,9	4X3	0	1	0,271	
				0	1	1	73,44	52,67	81,94	3X3	0	0	0,151	
				1	1	0								
				2	1	0								
				3	1	0								
				4	1	1	73,35	51,52	92,43	4X3	0	0	0,458	
				5	1	1	70,55	45,68	89,89	2X2	0	1	0,212	
				6	1	0								
				7	1	0								14:39
MP	1211	14:40	2	8	0									
				9	0									
				10	0									
				11	0									
				12	0									
				13	0									
				14	0									
				15	0									14:41
MP	1278	14:42	0	0	1	1	80,47	45,1	76,49	3X2	0	0	0,236	
				0	1	1	74,11	45,07	78,29	2X2	0	0	0,265	
				0	1	1	77,94	44,87	78,78	3X1	0	0	0,375	
				1	1	1	71,21	38,81	81,32	13X10	0	0	0,799	
				2	1	1	81,67	34,94	78,78	3X3	0	0	0,444	
				2	1	1	76,21	35,32	80,34	4X3	0	0	0,556	
				3	1	1	86,63	41,43	77,88	4X4	0	0	0,31	
				3	1	1	84,41	39,37	81,98	11X43	1	0	3,613	
				4	1	0								
				5	1	1	71,67	35,07	87,47	7X6	0	0	0,537	
				5	1	1	72,68	39,05	87,39	8X8	0	0	0,532	
				6	1	1	76,59	31,98	88,29	15X17	1	0	0,939	
				7	1	1	80,37	37,19	85,42	4X3	0	0	0,498	
				7	1	1	81,23	36,75	87,31	7X5	0	0	0,545	
				7	1	1	82,01	38,3	87,15	7X4	0	0	0,495	
				7	1	1	84,56	38,96	88,13	5X3	0	0	0,431	
				7	1	1	82,48	36,67	88,79	4X3	0	1	0,432	15:14
MP	1263	15:39	3	8	1	1	56,51	58,88	49,94	3x3	0	1	0,256	
				9	1	0								
				10	1	0								
				11	1	0								
				12	1	0								
				13	1	0								
				14	1	0								
				15	1	0								15:44
MP	1235	15:45	1	0	1	2								
				1	1	2								
				2	1	2								
				3	1	2								
				4	1	2								
				5	1	2								
				6	1	2								
				7	1	2								15:46
MP	1285	15:47	0	0	1	1	80,71	45,04	78,36	3X3	0	1	0,301	
				1	1	1	71,77	41,59	80,82	3X2	0	0	0,264	
				2	1	0								
				3	1	1	83,88	38,95	81,39	4X18	1	0	1,455	
				4	1	1	80,52	42,21	89,84	3X3	1	0	0,203	
				4	1	1	81,09	41,92	89,51	4X3	0	1	0,268	
				5	1	0								
				6	1	1	75,34	32,14	88,93	3X3	0	1	0,22	
				7	1	1	83,11	36,96	90,57	27X15	1	0	1,805	15:59
MP	1233	16:00	3	8	1	1	56,67	53,53	39,8	4x5	0	1	0,34	
				9	1	1	48,96	51,81	42,34	13x27	1	0	2,033	

				9	1	1	48,19	50,98	41,11	5x10	1	0	0,851	
				10	1	1	57,05	46,81	40,54	13x13	1	0	1,229	
				10	1	1	50,57	47,51	41,27	10x9	1	0	0,772	
				11	1	1	59,21	48,48	40,04	7x4	1	0	0,688	
				12	1	0								
				13	1	0								
				14	1	1	56,87	45,38	44,72	15x7	1	0	0,724	
				15	1	1	59,85	50,12	46,03	11x5	1	0	0,914	0,679861
MP	1206	0,40625	3	8	1	1	64,19	56,13	31,44	3X3	0	0	0,3	
				9	1	0								
				10	1	0								
				11	1	1	61,58	50,8	28,82	4X2	0	0	0,306	
				12	1	0								
				13	1	0								
				14	1	0								
				15	1	0								0,410417
MP	1215	0,4111111	0	0	1	0								
				1	1	0								
				2	1	0								
				3	1	0								
				4	1	1	40,79	36,74	76,9	5X5	0	1	0,373	
				5	1	1	46,42	32,05	74,85	3X4	0	1	0,344	
				6	1	0								
				7	1	1	36,65	33,08	77,48	23X40	1	0	3,157	0,41875
MP	1282	0,4194444	1	0	1	1	57,99	52,08	94,14	4X2	0	0	0,345	
				0	1	1	61,52	52,47	95,69	4X5	0	1	0,354	
				1	1	0								
				2	1	1	59,71	41,44	91,26	4X3	0	0	0,451	
				3	1	0								
				4	1	1	62,51	52,56	106,68	2X3	0	1	0,252	
				4	1	1	59,83	51,35	106,19	4X5	0	1	0,321	
				5	1	1	67,1	46,54	104,47	4X3	0	1	0,315	
				6	1	1	64,03	40,89	105,53	3X3	0	1	0,249	
				7	1	1	57,24	45,9	105,61	7X5	1	0	0,48	
				7	1	1	57,79	45,2	104,63	6X5	0	0	0,632	10:20
MP	1216	10:26	2	8	1	1	85,22	55,05	52,03	3X3	0	1	0,369	
				9	1	0								
				10	1	0								
				11	1	0								
				12	1	0								
				13	1	0								
				14	1	0								
				15	1	0								10:30
MP	1271	10:31	2	8	1	1	47,77	57,3	33,93	3X3	0	0	0,265	
				8	1	1	50,73	57,3	35	3X3	0	0	0,285	
				9	1	0								
				10	1	0								
				11	1	0								
				12	1	1	50,22	54,51	39,34	2X2	0	0	0,173	
				13	1	0								
				14	1	0								
				15	1	0								10:38
MP	1213	13:11	3	8	1	1	58,39	57,45	52,33	5X3	0	0	0,32	
				8	1	1	60,53	60,04	52,58	3X3	0	1	0,25	
				9	1	0								
				10	1	0								
				11	1	1	65,38	52,37	48,8	3X3	0	0	0,236	
				11	1	1	60,36	55,34	51,26	4X3	0	0	0,199	
				12	1	0								
				13	1	1	55,83	54,44	60,53	10X6	0	0	0,547	
				14	1	1	63,72	49,99	55,69	2X3	0	0	0,311	
				15	1	0								13:24
MP	1251	13:25	0	0	1	1	77,46	47,09	78,27	2X3	0	1	0,258	
				1	1	0								
				2	1	0								
				3	1	0								
				4	1	0								
				5	1	0								
				6	1	0								
				7	1	1	83,24	39,94	89,59	4X4	0	1	0,392	13:32
MP	1261	13:33	1	0	1	1	59,17	53,52	85,46	3x3	0	1	0,391	
				0	1	1	57,76	53,5	85,46	3x3	0	1	0,388	
				0	1	1	54,19	54,94	85,87	4x3	0	1	0,29	
				1	1	1	49,58	51,66	86,69	3x2	0	0	0,256	
				2	1	1	58,8	43,45	87,76	4x5	0	1	0,237	
				3	1	0								
				4	1	1	60,03	53,01	99,97	3x7	0	0	0,683	
				5	1	1	51,83	50,23	99,89	3x3	0	1	0,305	
				6	1	1	54,11	43,27	99,15	5x7	0	1	0,622	
				7	1	0								13:47
MP	1248	16:16	1	0	1	1	58,8	50,79	76,09	3X3	0	0	0,391	
				0	1	1	62,66	50,16	76,66	4X7	0	0	0,466	
				1	1	0								
				2	1	0								
				3	1	1	67,75	43,26	80,27	2X8	0	0	0,966	
				4	1	0								
				5	1	1	56	45,03	87,81	8X6	0	0	0,525	
				6	1	1	59,05	38,86	87,98	6X5	0	1	0,441	
				7	1	0								16:26
MP	1280	16:27	2	8	1	1	77,96	53,79	47,31	4x3	0	0	0,324	
				9	1	1	74,33	53,24	47,88	15x20	1	0	0,869	
				10	1	1	78,08	47,38	46,16	4x3	0	0	0,343	
				11	1	1	79,99	52,31	47,31	35x30	1	0	2,406	
				12	1	0								
				13	1	0								
				14	1	0								
				15	1	1	80,01	48,72	48,54	5x5	0	0	0,519	16:44
MP	1267	10:02	3	8	1	1	62,14	54,96	46,28	7x5	0	1	0,365	
				9	1	0								
				10	1	0								
				11	1	0								
				12	1	0								
				13	1	0								
				14	1	0								
				15	1	0								10:05
MP	1232	10:06	1	0	1	1	59,2	45,8	77,72	4X4	0	0	0,367	
				0	1	1	63,57	46,33	78,05	2X4	0	0	0,582	
				1	1	1	68,6	43,24	80,35	2X3	0	1	0,277	
				2	1	0								
				3	1	1	54,88	40,67	78,38	2X2	0	0	0,281	
				4	1	1	60,21	44,61	90,19	3X3	0	0	0,425	
				5	1	0								
				6	1	0								
				7	1	0								10:19
MP	1244	10:22	0	0	1	1	43,14	51,09	69,1	2x2	0	1	0,231	
				1	1	1	50,36	48,02	68,61	3x2	0	0	0,258	
				2	1	0								
				3	1	1	37,22	47,59	70,08	14x18	1	0	1,451	
				3	1	1	37,38	44,87	68,44	7x4	0	1	0,633	
				4	1	1	43,71	50,18	80,33	6x7	0	0	0,789	
				5	1	0								
				6	1	1	45,44	39,73	79,19	2x6	0	0	0,465	
				7	1	0								10:35
MP	1245	10:49	3	8	1	1	56,21	55,97	29,87	3X3	0	1	0,263	
				9	1	0								
				10	1	0								
				11	1	0								
				12	1	0								
				13	1	1	49,41	54,44	37,33	10X9	1	0	0,676	
				14	1	0								
				15	1	0								10:54
MP	1217	10:55	2	8	1	0								
				9	1	0								
				10	1	0								
				11	1	0								
				12	1	0								
				13	1	1	45,63	51,98	47,16	11X3	0	0	0,843	
				14	1	1	34,83	47,4	47,81	5X4	0	0	0,4	
				15	1	1	35,35	51,51	44,94	16X11	1	0	1,283	
				15	1	1	35,31	54,43	48,22	18X11	1	0	1,061	11:04
MP	1241	15:00	1	0	1	1	56,49	50,38	71,19	3x3	0	1	0,239	
				0	1	1	56,91	49,88	73,48	12x10	1	0	0,7	
				1	1	1	51,74	45,12	73,4	18x27	1	0	1,778	

				2	1	0								
				3	1	1	63,06	43,93	73,15	4x10	1	0	0,747	
				3	1	1	61,98	41,31	72,58	7x6	0	0	0,551	
				3	1	1	63,71	45,26	70,12	4x3	0	0	0,22	
				4	1	2								
				5	1	2								
				6	1	2								
				7	1	2								15:14
MP	1242	15:15	1	0	1	1	52,8	45,47	71,97	5X4	0	1	0,43	
				0	1	1	58,73	44,08	71,97	3X3	0	1	0,318	
				1	1	0								
				2	1	0								
				3	1	1	61,43	35,89	73,53	7X32	1	0	3,724	
				4	1	0								
				5	1	0								
				6	1	0								
				7	1	1	59,82	34,99	81,89	4X5	1	0	0,512	15:22
MP	1259	15:23	1	0	1	1	57,92	51,7	86,99	7X7	0	1	0,636	
				0	1	1	54,59	51,03	86,66	4X4	0	1	0,453	
				1	1	0								
				2	1	0								
				3	1	0								
				4	1	1	60,81	47,9	98,71	3X4	0	0	0,468	
				5	1	0								
				6	1	0								
				7	1	0								15:30
MP	1292	15:31	2	8	1	0								
				9	1	0								
				10	1	0								
				11	1	0								
				12	1	0								
				13	1	0								
				14	1	0								
				15	1	1	29,36	53,24	43,54	26X37	1	0	2,829	0,649306
MP	1256	15:36	2	8	1	1	76,48	53,4	54,13	3X3	0	0	0,323	
				8	1	1	80,86	51,62	55,77	3X4	0	1	0,293	
				9	1	0								
				10	1	1	80,57	44,31	53,07	4X2	0	1	0,173	
				11	1	0								
				12	1	0								
				13	1	0								
				14	1	0								
				15	1	0								15:41
MP	1281	17:38	1	0	1	1	64,5	46,96	89,79	3x3	0	0	0,228	
				0	1	1	59,82	48,04	93,4	3x3	0	1	0,47	
				0	1	1	61,77	47,72	93,4	4x5	0	1	0,433	
				0	1	1	58,37	47,69	96,27	7x8	0	0	0,626	
				1	1	1	52,54	43,32	96,03	6x6	0	0	0,556	
				2	1	0								
				3	1	1	63,79	40,56	97,25	5x5	1	0	0,506	
				4	1	0								
				5	1	0								
				6	1	0								
				7	1	1	60,66	35,41	104,55	19x17	1	0	1,562	
				7	1	1	62,54	37,7	103,73	5x4	1	0	0,329	
				7	1	1	62,37	37,71	102,83	2x3	0	0	0,226	17:56
MP	1283	17:58	1	0	0									
				1	0									
				2	0									
				3	0									
				4	0									
				5	0									
				6	0									
				7	0									17:59
MP	1279	18:01	1	0	1	1	67,96	44,97	84,26	3X2	0	0	0,165	
				1	1	0								
				2	1	0								
				3	1	0								
				4	1	0								
				5	1	1	69,41	41,1	97,46	3X3	0	0	0,285	
				6	1	1	66,79	34,03	96,97	5x7	0	0	0,513	
				6	1	1	66,04	33,63	95,74	3x3	0	0	0,675	
				7	1	1	61,11	39,31	94,43	26x27	1	0	2,142	
				7	1	1	62,38	37,21	92,05	2x3	0	0	0,472	18:19
MP	1226	16:37	2	8	1	0								
				9	1	1	74,94	47,91	32,17	3X3	0	0	0,418	
				10	1	1	82,58	45,96	29,71	27X27	1	0	1,826	
				11	1	1	85,49	47,74	32,34	7X7	1	0	0,916	
				12	1	0								
				13	1	1	76,89	46,5	34,14	10X7	1	0	0,719	
				13	1	1	74,15	46,52	36,76	15X30	1	0	2,822	
				14	1	1	80,58	42,54	36,76	28X30	1	0	2,619	
				15	1	1	85,29	46,46	37,83	41X22	1	0	1,757	
				15	1	1	83,33	44,33	34,47	5X8	0	0	0,85	16:58
MP	1277	17:02	3	8	1	1	61,41	53,57	40,92	4X3	0	0	0,361	
				8	1	1	69,8	55,19	41,98	3X3	0	0	0,188	
				8	1	1	60,97	53,44	42,64	4X3	0	0	0,289	
				8	1	1	65,34	54,88	42,8	3X3	0	1	0,325	
				8	1	1	70,1	55,48	44,2	3X3	0	0	0,358	
				8	1	1	66,4	54,97	45,59	3X2	0	1	0,188	
				9	1	1	72,07	53,72	41,24	3X2	0	0	0,196	
				10	1	1	64,71	47,47	41	3X3	0	0	0,169	
				11	1	0								
				12	1	0								
				13	1	0								
				14	1	1	67,91	46,02	50,76	3X3	0	0	0,313	
				15	1	0								17:23
MP	1276	17:28	2	8	1	1	43,71	57,21	32,04	3X5	0	1	0,623	
				9	1	0								
				10	1	1	43,15	40,45	31,05	3X3	0	0	0,302	
				11	1	0								
				12	1	0								
				13	1	1	50,28	53,4	34,5	7X6	1	0	1,379	
				14	1	0								
				15	1	0								17:36
MP	1205	17:40	3	8	1	0								
				9	1	0								
				10	1	0								
				11	1	0								
				12	1	1	59,42	51,03	44,17	2X3	0	0	0,274	
				13	1	1	51,54	53,61	39,25	8X8	1	0	0,499	
				14	1	0								
				15	1	1	61,45	51,7	39,34	9X12	1	0	0,746	
				15	1	1	61,9	52	41,55	9X9	1	0	0,495	17:48
MP	1234	17:49	3	8	1	0								
				9	1	1	71,21	51,22	42,41	3X2	0	0	0,438	
				10	1	0								
				11	1	0								
				12	1	0								
				13	1	0								
				14	1	0								
				15	1	0								17:54
MP	1207	17:55	1	0	1	1	59,26	49,27	76,5	3X3	0	1	0,306	
				0	1	1	56	49,34	76,75	4X4	0	1	0,338	
				0	1	1	54,55	49,48	76,67	2X2	0	0	0,281	
				1	1	0								
				2	1	0								
				3	1	0								
				4	1	0								
				5	1	0								
				6	1	0								
				7	1	1	62,45	41,17	87,41	8X6	1	0	0,398	18:04
MP	1225	9:42	0	0	1	1	45,98	49,53	75,92	3X3	0	1	0,398	
				0	1	1	43,17	50,47	84,2	4X5	0	0	0,559	
				1	1	1	49,96	44,03	80,43	10X15	1	0	1,333	
				2	1	1	45,84	40,88	82,23	8X7	0	0	0,79	
				3	1	0								
				4	1	2								
				5	1	2								
				6	1	2								
				7	1	2								10:12
MP	1269	10:24	0	0	1	1	46,16	50,2	69,08	4X5	0	1	0,387	
				1	1	1	53,25	46,26	69,74	4X3	0	0	0,519	
				2	1	0								
				3	1	1	41,1	42,59	71,21	2X2	1	0	0,217	

				4	0									
				5	0									
				6	0									
				7	0									10:29
MP	1231	10:39	0	0	1	1	77,41	38,83	64,15	4x2	0	1	0,186	
				0	1	1	81,58	38,2	64,72	3x3	0	1	0,26	
				1	1	0								
				2	1	0								
				3	1	0								
				4	1	1	82,02	35,19	75,22	2x3	0	0	0,26	
				5	1	1	74,84	31,03	74,81	2x2	0	1	0,322	
				6	1	1	74,63	25,39	76,78	4x5	0	0	0,818	
				7	1	0								10:48
MP	1224	10:49	0	0	1	1	80,45	50,53	77,49	5x3	0	1	0,259	
				1	1	0								
				2	1	0								
				3	1	1	84,63	43,17	80,63	7x8	1	0	1,438	
				4	1	2								
				5	1	2								
				6	1	2								
				7	1	2								10:54
MP	1222	10:55	1	0	1	1	64,39	55,03	85,78	2X4	0	1	0,334	
				0	1	1	66,06	55,14	89,95	3X2	0	1	0,323	
				1	1	0								
				2	1	0								
				3	1	0								
				4	1	0								
				5	1	0								
				6	1	1	66,52	43,16	97,59	5X4	0	1	0,544	
				7	1	1	63,18	46,44	92,83	10X8	1	0	0,753	
				7	1	1	61,16	48,42	96,11	14X15	1	0	2,173	11:03
MP	1296	11:04	3	8	1	2								
				9	1	2								
				10	1	2								
				11	1	2								
				12	1	0								
				13	1	1	54,97	52,85	34,88	3X3	0	0	0,29	
				14	1	0								
				15	1	0								11:07
