# Supplementary material for: Development of a scoring method to visually score cortical interruptions on high-resolution peripheral quantitative computed tomography in rheumatoid arthritis and healthy controls
Source: PLoS One. 2018 Jul 9;13(7):e0200331. doi: 10.1371/journal.pone.0200331 (PMC6037386; doi:10.1371/journal.pone.0200331)
Supplement: S4 File — (PDF) [file pone.0200331.s004.pdf]

Reader initials	studyID	start time	joint (0=MCP2, 1=	surface (0= palma	evaluable (0=no 1=yes)	discontinuity (0=no 1=yes 2=total d	x value	y value	z value	planes	adjacent trabecular d	parallel structure (0	maximal diameter	end time	total time
MP	1246	15:19	3	8	1	0									
	1246			9	1	0									
	1246			10	1	0									
	1246			11	1	0									
	1246			12	1	0									
	1246			13	1	1	51,82	56,77	36,68	10x7	1	0	0,756		
	1246			14	1	0									
	1246			15	1	0								15:22	
MP	1294	15:24	1	0	0										
	1294			1	0										
	1294			2	0										
	1294			3	0										
	1294			4	1	0									
	1294			5	1	0									
	1294			6	1	0									
	1294			7	1	0								15:27	
MP	1200	15:28	0	0	1	1	83,09	46,32	94,5	3X4	0	1	0,323		
	1200			0	1	1	80,17	45,95	94,66	2X2	0	1	0,11		
	1200			0	1	1	82,43	45,98	98,02	7X10	1	0	0,975		
	1200			1	1	1	75,7	40,7	97,04	4X5	0	0	0,888		
	1200			2	1	1	86,28	36,64	95,73	6X4	0	0	0,335		
	1200			3	1	1	89,81	44,23	95,89	14X19	1	9	1,727		
	1200			3	1	1	90,52	41,29	93,43	5X3	0	0	0,323		
	1200			4	1	1	86,05	40,25	105,65	6X6	0	1	0,765		
	1200			5	1	1	78,51	35,04	100,24	18X20	1	0	2,042		
	1200			6	1	1	85,64	31,04	101,63	7X7	0	0	0,659		
	1200			6	1	1	81,06	29,6	102,7	5X9	0	0	0,765		
	1200			7	1	1	87,61	33,66	100,4	19X6	1	0	0,996	15:49	
MP	1264	15:58	0	0	1	1	76,4	45,16	77,68	3X3	0	1	0,201		
	1264			0	1	1	77,51	45,52	79,89	3X3	0	0	0,268		
	1264			0	1	1	81,46	45,85	80,22	2X2	0	0	0,226		
	1264			1	1	0									
	1264			2	1	0									
	1264			3	1	1	88	42,67	79,65	3X3	0	0	0,144		
	1264			4	1	1	80,13	43,35	91,13	5X11	0	0	0,798		
	1264			5	1	1	73,82	33,25	94,32	3X3	0	0	0,304		
	1264			6	1	0									
	1264			7	1	0									
MP	1255	16:11	3	8	1	1	66,6	58,96	31,42	4X3	0	0	0,341		
	1255			9	1	0									
	1255			10	1	0									
	1255			11	1	0									
	1255			12	0										
	1255			13	0										
	1255			14	0										
	1255			15	0									16:14	
MP	1273	16:15	3	8	1	1	61,63	54,35	51,37	9X6	0	1	0,651		
	1273			9	1	0									
	1273			10	1	1	57,57	47	51,7	3X5	0	0	0,345		
	1273			11	1	1	66,88	47,26	50,63	13X8	1	0	1,032		
	1273			12	1	0									
	1273			13	1	1	55,76	47,5	54,98	6X6	1	0	0,461		
	1273			14	1	0									
	1273			15	1	0								16:27	
MP	1237	16:40	0	0	1	0									
	1237			1	1	0									
	1237			2	1	1	45,23	38,47	66,85	3X4	0	0	0,493		
	1237			2	1	1	42,8	39,56	70,3	7X5	0	0	1,183		
	1237			3	1	1	39,72	41,6	68,41	6X4	0	0	0,524		
	1237			4	1	0									
	1237			5	1	0									
	1237			6	1	1	50,96	35,06	78,74	2X2	0	1	0,163		
	1237			7	1	1	44,1	38,14	75,87	3X4	0	0	0,219		
	1237			7	1	1	41,8	40,44	82,6	3X3	0	0	0,232	16:54	
MP	1212	16:55	2	8	1	1	43,29	54,88	47,04	4X3	0	1	0,206		
	1212			8	1	1	48,32	54,93	48,35	6X5	0	1	0,281		
	1212			9	1	0									
	1212			10	1	1	43,77	50,68	44,08	3X2	0	0	0,32		
	1212			11	1	0									
	1212			12	1	1	43,9	53,31	55,24	3X4	0	0	0,433		
	1212			13	1	0									
	1212			14	1	1	47,61	47,42	50,73	3X3	0	0	0,332		
	1212			15	1	0								17:07	
MP	1210	17:10	3	8	1	1	65,04	58,18	42,03	6X3	0	1	0,436		
	1210			8	1	1	66,08	58,35	41,78	3X3	0	1	0,161		
	1210			8	1	1	67,4	58,83	43,18	4X3	0	1	0,433		
	1210			9	1	0									
	1210			10	1	1	66,47	49,56	42,36	2X2	0	0	0,219		
	1210			11	1	0									
	1210			12	1	0									
	1210			13	1	1	70,96	55,3	48,76	18X8	1	0	0,861		
	1210			14	1	1	66,63	50,48	47,77	2X4	0	1	0,263		
	1210			15	1	0								17:22	
MP	1284	9:49	3	8	1	0									
	1284			9	1	0									
	1284			10	1	0									
	1284			11	1	0									
	1284			12	1	0									
	1284			13	1	1	68,17	55,7	60,14	3x3	0	0	0,305		
	1284			13	1	1	67,67	56,54	58,09	27x22	1	0	1,385		
	1284			14	1	0									
	1284			15	1	1	57,34	54,57	57,35	8x5	0	0	0,637	9:56	
MP	1249	9:58	3	8	1	1	63,36	54,84	40,75	2X4	0	1	0,475		
	1249			9	1	0									
	1249			10	1	0									
	1249			11	1	0									
	1249			12	1	0									
	1249			13	1	0									
	1249			14	1	0									
	1249			15	1	1	65,26	50,8	45,75	20X13	1	0	1,346	10:04	
MP	1254	10:07	1	0	1	1	62,88	50,78	87,22	4x4	0	1	0,454		
	1254			0	1	1	59,35	51,48	89,1	4x5	0	0	0,554		
	1254			1	1	0									
	1254			2	1	1	58,54	41,52	90,66	5x5	0	1	0,473		
	1254			3	1	1	63,4	43,54	91,24	13x44	1	0	4,567		
	1254			4	1	1	62,71	49,47	101,07	7x6	0	1	1,001		
	1254			4	1	1	58,84	53,37	102,88	5x4	0	0	0,465		
	1254			5	1	0									
	1254			6	1	0									
	1254			7	1	1	61,98	43,72	95,66	33x15	1	0	1,995		
	1254			7	1	1	61,98	44,42	98,53	18x15	1	0	1,024		

	1254			7	1	1	65,01	45,23	101,4	13x8	1	0	1,069	
	1254			7	1	1	66,03	47,09	101,89	7x7	1	0	0,991	
	1254			7	1	1	65,77	46,6	103,21	3x2	0	0	0,299	10:30
MP	1230	16:06	1	0	1	1	58,84	57	91,6	4X5	0	1	0,452	
	1230			0	1	1	61,75	56,38	91,93	6X6	0	1	0,501	
	1230			0	1	1	64,47	56,57	92,5	4x4	0	1	0,387	
	1230			1	1	0								
	1230			2	0									
	1230			3	1	0								
	1230			4	1	0								
	1230			5	1	1	53,35	50,84	103,08	3x3	0	0	0,191	
	1230			5	1	1	54,04	52,16	105,87	4x4	0	0	0,47	
	1230			6	1	0								
	1230			7	1	0								16:17
MP	1208	16:23	1	0	1	0								
	1208			1	1	0								
	1208			2	1	0								
	1208			3	1	0								
	1208			4	1	0								
	1208			5	1	0								
	1208			6	1	0								
	1208			7	1	0								16:27
MP	1275	16:28	2	8	1	1	80	55,3	49,28	4X4	0	0	0,211	
	1275			9	1	0								
	1275			10	1	1	80,17	48,19	46,91	3X3	0	0	0,267	
	1275			10	1	1	79,22	47,37	50,1	4X3	0	0	0,477	
	1275			11	1	0								
	1275			12	1	1	78,21	51,07	56,91	4X2	0	1	0,267	
	1275			13	1	0								
	1275			14	1	1	79,92	46,01	52,89	4X4	0	0	0,363	
	1275			15	1	0								16:38
MP	1302	16:39	0	0	1	1	83,86	50,05	73,49	7X4	0	0	0,46	
	1302			0	1	1	79,99	50,6	76,86	6X7	0	1	0,495	
	1302			0	1	1	77,44	51,19	76,77	3X3	0	1	0,28	
	1302			0	1	1	82,74	51,05	76,69	2X2	0	1	0,208	
	1302			1	1	0								
	1302			2	1	0								
	1302			3	1	0								
	1302			4	1	1	81,38	48,24	89,32	3X4	0	0	0,575	
	1302			5	1	0								
	1302			6	1	1	77,31	37,77	87,6	3X3	0	1	0,333	
	1302			7	1	0								16:52
MP	1239	16:53	2	8	1	0								
	1239			9	1	0								
	1239			10	1	0								
	1239			11	1	0								
	1239			12	1	0								
	1239			13	1	0								
	1239			14	1	0								
	1239			15	1	1	35,97	55,48	39,05	7X5	0	0	0,745	
	1239			15	1	1	36,14	55,05	39,54	4X6	1	0	0,634	16:58
MP	1223	14:56	3	8	1	1	55,74	56,17	40,12	3x3	0	1	0,252	
	1223			8	1	1	62,01	53,93	45,28	5x4	0	0	0,288	
	1223			9	1	1	52,42	52,17	44,63	6x14	1	0	1,552	
	1223			10	1	1	56,42	46,88	42,58	3x3	0	1	0,273	
	1223			11	1	1	63,35	47,69	44,55	6x11	1	0	1,526	
	1223			12	1	1	63,74	50,75	51,76	4X3	0	0	0,263	
	1223			13	1	1	51,86	50,72	48,24	4x4	0	0	0,265	
	1223			14	1	1	55,06	46,87	49,47	5x4	0	0	0,504	
	1223			14	1	1	52,96	48,46	48,73	6x3	0	0	0,398	
	1223			15	1	1	62,18	47,64	46,76	4x3	0	0	0,668	15:15
MP	1262	15:18	2	8	1	1	72,84	54,79	46,29	3X2	0	0	0,189	
	1262			9	1	0								
	1262			10	1	0								
	1262			11	1	1	75,64	50,73	42,52	6X3	0	1	0,39	
	1262			11	1	1	75,12	50,88	41,37	3X3	0	1	0,193	
	1262			12	1	0								
	1262			13	1	0								
	1262			14	1	0								
	1262			15	1	0								15:25
MP	1291	15:33	0	0	1	1	78,47	46,24	76,52	3x2	0	1	0,402	
	1291			1	1	1	73,58	43,23	80,13	3x4	0	1	0,398	
	1291			2	1	0								
	1291			3	1	1	88,41	39,73	80,79	25x33	1	0	2,826	
	1291			3	1	1	87,13	39,46	83,9	4x5	0	0	1,167	
	1291			4	1	1	85,16	43,87	90,14	7X7	0	0	0,662	
	1291			4	1	1	83,12	43,96	92,51	6x6	0	1	0,543	
	1291			5	1	1	75,75	38,87	90,54	2X2	0	1	0,265	
	1291			6	1	1	78,79	32,31	91,61	4x5	0	1	0,48	
	1291			6	1	1	78,79	32,91	90,54	11X11	1	0	0,784	
	1291			6	1	1	76,24	34,07	85,54	6X12	1	0	1,066	
	1291			7	1	1	83,69	37,17	86,61	30X18	1	0	1,733	
	1291			7	1	1	85,66	38,5	90,96	70X39	1	0	3,37	15:56
MP	1287	15:59	1	0	1	1	63,93	49,21	68,21	3X2	0	1	0,162	
	1287			1	1	1	54,12	43,11	73,54	3X3	0	0	0,295	
	1287			2	1	0								
	1287			3	1	0								
	1287			4	1	0								
	1287			5	1	0								
	1287			6	1	0								
	1287			7	1	1	65,41	41,18	82,32	9X8	1	0	1,062	16:07
MP	1257	13:19	0	0	1	1	82,94	44,39	65,87	4X3	0	1	0,27	
	1257			1	1	0								
	1257			2	1	0								
	1257			3	1	1	90,78	37,21	64,06	3X4	0	0	0,261	
	1257			4	1	1	86,33	41,68	75,87	3X4	0	0	0,55	
	1257			5	1	0								
	1257			6	1	1	82,78	29,93	75,71	3X3	0	0	0,27	
	1257			7	1	1	89,37	36,19	75,95	3X3	0	0	0,296	13:32
MP	1297	13:34	3	8	1	0								
	1297			9	1	0								
	1297			10	1	1	56,35	49,22	31,8	3X2	0	0	0,204	
	1297			11	1	1	63,92	49,83	29,33	4X3	0	0	0,304	
	1297			12	1	0								
	1297			13	1	0								
	1297			14	1	0								
	1297			15	1	1	64,53	49,12	37,78	5X5	0	0	0,332	
	1297			15	1	1	66	52,8	36,22	4X3	0	0	0,277	13:43
MP	1298	13:44	2	8	0									
	1298			9	0									

	1298			10	0									
	1298			11	0									
	1298			12	0									
	1298			13	0									
	1298			14	0									
	1298			15	0									13:44
MP	1247	13:45	2	8	1	1	47,25	57,83	38,77	3X3	0	0	0,261	
	1247			8	1	1	42,37	58,41	38,28	5X4	0	1	0,323	
	1247			8	1	1	45,14	58,24	38,2	3X3	0	1	0,32	
	1247			9	1	1	49,06	55,59	34,51	3X5	0	0	0,432	
	1247			9	1	1	49	53,87	37,46	3X3	0	1	0,292	
	1247			10	1	0								
	1247			11	1	0								
	1247			12	1	0								
	1247			13	1	0								
	1247			14	1	0								
	1247			15	1	0								13:55
MP	1253	14:22	1	0	1	1	58,33	48,64	95,81	4X4	0	1	0,311	
	1253			1	1	1	49,44	47,13	98,27	3X2	0	0	0,253	
	1253			2	1	0								
	1253			3	1	0								
	1253			4	1	0								
	1253			5	1	0								
	1253			6	1	1	55,73	36,12	106,23	3X3	0	1	0,313	
	1253			7	1	0								14:31
MP	1252	14:36	2	8	1	1	81,76	56,16	37,9	4X5	0	0	0,381	
	1252			8	1	1	72,84	57,67	39,54	3X3	0	0	0,284	
	1252			8	1	1	78,59	57,02	39,54	4X5	0	1	0,42	
	1252			8	1	1	79,74	57,18	39,63	3X3	0	0	0,34	
	1252			8	1	1	76,2	57,76	40,37	4X4	0	0	0,498	
	1252			9	1	1	71,73	53,16	39,96	5X3	0	0	0,393	
	1252			10	1	0								
	1252			11	1	1	83,78	53,68	37,41	3X2	0	0	0,214	
	1252			11	1	1	82,34	51,07	38,4	3X3	0	0	0,319	
	1252			12	1	0								
	1252			13	1	1	72,85	56,52	45,86	3X3	0	0	0,398	
	1252			14	1	0								
	1252			15	1	1	81,41	51,12	42,58	3X3	0	0	0,202	14:51
MP	1227	17:25	2	8	1	2								
	1227			9	1	2								
	1227			10	1	2								
	1227			11	1	2								
	1227			12	1	2								
	1227			13	1	2								
	1227			14	1	2								
	1227			15	1	2								17:27
MP	1268	17:28	0	0	1	1	82,5	49,73	87,71	9X7	0	1	0,657	
	1268			0	1	1	78,88	48,92	88,78	8X8	0	1	0,543	
	1268			1	1	0								
	1268			2	1	1	81,04	39,22	84,68	6X5	0	0	0,408	
	1268			3	1	0								
	1268			4	1	0								
	1268			5	1	1	74,35	42,31	97,47	6x3	0	0	0,544	
	1268			5	1	1	73,13	38,87	96,24	3x3	0	0	0,208	
	1268			6	1	1	79,05	36,12	97,96	3x4	0	1	0,379	
	1268			7	1	0								17:40
MP	1202	17:42	2	8	1	1	82,57	55,12	35,99	5X5	0	0	0,445	
	1202			8	1	1	79,92	55,73	36,32	5X4	0	1	0,328	
	1202			9	1	1	75,44	51,86	33,86	3X2	0	1	0,236	
	1202			10	1	1	83,63	48,15	34,11	3X3	0	0	0,492	
	1202			11	1	1	85,97	53,18	31,73	6X5	0	0	0,37	
	1202			12	1	0								
	1202			13	1	0								
	1202			14	1	0								
	1202			15	1	0								17:53
MP	1265	17:55	2	8	1	0								
	1265			9	1	1	75,53	51,44	43,85	3X2	0	0	0,377	
	1265			10	1	0								
	1265			11	1	0								
	1265			12	1	0								
	1265			13	1	0								
	1265			14	1	0								
	1265			15	1	1	83,19	46,14	48,04	6X5	0	0	0,379	18:03
MP	1204	17:19	1	0	1	1	60,25	53,57	92,16	5x5	0	1	0,499	
	1204			0	1	1	62,15	53,7	92,65	4x4	0	1	0,473	
	1204			0	1	1	64,65	53,84	93,22	5x5	0	1	0,512	
	1204			0	1	1	62,99	53,76	95,68	23x28	1	0	2,292	
	1204			1	1	0								
	1204			2	1	0								
	1204			3	1	0								
	1204			4	1	1	61,96	53,17	106,75	3x2	0	0	0,445	
	1204			4	1	1	59,51	52,37	106,51	5x4	0	0	0,397	
	1204			5	1	1	66,59	51,33	104,62	20x16	0	0	1,531	
	1204			5	1	1	63,78	44,85	105,03	6x7	0	0	1,23	
	1204			5	1	1	66,31	45,81	100,68	3x3	0	1	0,33	
	1204			6	1	0								
	1204			7	1	1	54,66	48,62	106,51	12x11	1	0	0,946	
	1204			7	1	1	56,36	45,56	105,19	11x19	1	0	1,373	
	1204			7	1	1	55,3	47,47	103,96	4x4	1	0	0,403	
	1204			7	1	1	56,83	43,91	100,27	13x11	1	0	0,767	17:41
MP	1272	17:34	1	0	1	1	68,71	52,26	84,01	4X3	0	1	0,284	
	1272			0	1	1	66,72	52,71	87,38	6X5	0	1	0,54	
	1272			1	1	0								
	1272			2	1	1	62,3	42,19	89,02	6X5	0	1	0,491	
	1272			3	1	1	58,53	46,8	90,57	20X20	1	0	1,591	
	1272			4	1	1	63,26	51,74	101,89	8X7	0	0	1,061	
	1272			5	1	1	69,86	46,89	99,02	3X3	0	1	0,272	
	1272			6	1	0								
	1272			7	1	1	60,99	44,74	97,38	20x15	1	0	1,333	
	1272			7	1	1	60,38	45,27	96,07	9x5	1	0	0,716	17:54
MP	1220	17:56	3	8	1	1	62,85	51,88	40,74	4X3	0	0	0,55	
	1220			9	1	0								
	1220			10	1	0								
	1220			11	1	0								
	1220			12	1	0								
	1220			13	1	0								
	1220			14	1	1	68,62	43,76	46,81	6X5	0	0	0,401	
	1220			14	1	1	62,07	41,73	44,92	5X4	0	0	0,426	
	1220			15	1	0								18:04
MP	1300	16:30	3	8	1	1	57,6	51,74	42,11	6X5	0	1	0,405	
	1300			8	1	1	54,73	53,41	42,93	4X4	0	1	0,435	

	1300			9	1	0								
	1300			10	1	0								
	1300			11	1	0								
	1300			12	1	0								
	1300			13	1	0								
	1300			14	1	1	54,49	42,72	46,54	3x3	0	0	0,236	
	1300			15	1	0								16:39
MP	1243	16:40	0	0	1	1	42,13	51,11	74,8	4X3	0	1	0,23	
	1243			0	1	1	46,11	49,81	75,7	4X4	0	1	0,367	
	1243			1	1	0								
	1243			2	1	0								
	1243			3	1	0								
	1243			4	1	1	44,22	48,74	87,26	7X7	0	0	0,629	
	1243			5	1	0								
	1243			6	1	0								
	1243			7	1	1	37,92	45,24	88,99	3X5	0	0	0,316	16:49
MP	1274	17:03	3	8	1	0								
	1274			9	1	1	67,23	50,47	53,9	3X4	0	0	0,366	
	1274			10	1	1	62,58	46,51	51,77	3X2	0	0	0,225	
	1274			10	1	1	65,72	47,29	55,13	7X5	0	0	0,416	
	1274			11	1	0								
	1274			12	1	0								
	1274			13	1	0								
	1274			14	1	0								
	1274			15	1	0								17:11
MP	1301	17:15	2	8	1	1	78,86	58,28	41,81	2x2	0	0	0,189	
	1301			9	1	0								
	1301			10	1	1	76,64	51,04	42,63	5x3	0	0	0,466	
	1301			11	1	0								
	1301			12	1	0								
	1301			13	1	0								
	1301			14	1	0								
	1301			15	1	0								17:21
MP	1289	17:41	0	0	1	1	46,18	51,94	71,53	6x7	0	1	0,425	
	1289			1	1	0								
	1289			2	1	0								
	1289			3	1	0								
	1289			4	1	0								
	1289			5	1	0								
	1289			6	1	0								
	1289			7	1	0								17:48
MP	1250	17:49	2	8	1	1	84,05	53,1	48,61	6X5	0	0	0,375	
	1250			9	1	1	77,86	52,51	45,16	4X3	0	0	0,266	
	1250			10	1	0								
	1250			11	1	0								
	1250			12	1	0								
	1250			13	1	0								
	1250			14	1	0								
	1250			15	1	0								17:55
MP	1270	9:49	2	8	1	1	80,98	52,39	44,32	4X4	0	0	0,534	
	1270			9	1	1	75,8	49,73	41,77	5X3	0	0	0,668	
	1270			9	1	1	76,72	47,41	42,43	9X16	1	0	2,108	
	1270			10	1	0								
	1270			11	1	0								
	1270			12	1	0								
	1270			13	1	0								
	1270			14	1	0								
	1270			15	1	0								9:58
MP	1303	10:02	3	8	1	1	72,45	50,07	44,32	4X4	0	1	0,55	
	1303			9	1	1	77,73	46,33	43,09	4X14	1	0	1,427	
	1303			10	1	1	70,18	42,6	43,25	15X25	1	0	2,356	
	1303			11	1	1	65,79	46,18	41,28	5X4	0	0	0,246	
	1303			11	1	1	66,17	46,65	42,59	4X5	0	0	0,624	
	1303			11	1	1	66,44	45,36	41,86	7X10	1	0	1,411	
	1303			11	1	1	68	44,07	42,92	16X7	1	0	1,846	
	1303			12	0									
	1303			13	1	1	76,05	47,36	50,3	7X4	0	0	0,673	
	1303			13	1	1	75,81	44,36	46,69	7X7	0	0	0,374	
	1303			14	0									
	1303			15	1	1	68,16	42,99	46,2	22X28	1	0	2,111	10:25
MP	1258	15:44	1	0	1	1	58,83	55,58	93,6	4x4	0	1	0,266	
	1258			0	1	1	62,44	54,31	93,52	3x3	0	1	0,19	
	1258			1	1	0								
	1258			2	1	1	60,12	44,88	94,01	3x3	0	0	0,224	
	1258			3	1	0								
	1258			4	1	1	60,58	53,39	106,89	3x3	0	1	0,222	
	1258			5	1	0								
	1258			6	1	0								
	1258			7	1	0								15:55
MP	1240	15:56	1	0	1	1	68,32	53,45	82,28	6X5	0	0	0,402	
	1240			0	1	1	67,25	54,13	84,82	2X3	0	1	0,286	
	1240			1	1	0								
	1240			2	1	1	70,59	46,8	80,8	4X2	0	1	0,227	
	1240			3	1	0								
	1240			4	1	1	61,29	51,38	97,53	5X6	0	0	0,585	
	1240			5	1	0								
	1240			6	1	0								
	1240			7	1	1	62,08	47,79	95,48	13X8	1	0	1,294	16:09
MP	1288	16:19	0	0	1	1	78,85	52,52	88	3x6	0	1	0,37	
	1288			1	1	0								
	1288			2	1	0								
	1288			3	1	0								
	1288			4	1	0								
	1288			5	1	0								
	1288			6	1	1	76,13	39,5	97,43	7x6	0	1	0,503	
	1288			7	1	1	84,02	46,3	100,13	4x3	0	0	0,315	16:27
MP	1260	14:19	3	8	1	1	66,94	55,01	26,39	4x5	0	0	0,547	
	1260			9	1	0								
	1260			10	1	0								
	1260			11	1	0								
	1260			12	1	0								
	1260			13	1	0								
	1260			14	1	0								
	1260			15	1	0								14:23
MP	1201	14:24	0	0	1	1	51,33	53,41	82,14	5X4	0	0	0,356	
	1201			0	1	1	44,91	55,95	85,91	3X4	0	0	0,416	
	1201			1	1	0								
	1201			2	1	0								
	1201			3	1	1	39,3	48,39	87,47	2X4	0	0	0,169	
	1201			4	1	0								

	1201			5	1	1	50,96	51,37	97,8	4X3	0	1	0,278	
	1201			6	1	1	46,74	44,71	96,9	2X4	0	1	0,175	
	1201			7	1	1	40,4	50,81	100,18	9X6	0	0	0,655	14:40
MP	1299	14:45	0	0	1	1	44,83	41,77	72,18	5X4	0	1	0,395	
	1299			0	1	1	46,71	41,39	72,35	4X5	0	1	0,398	
	1299			1	1	0								
	1299			2	1	0								
	1299			3	1	0								
	1299			4	0									
	1299			5	0									
	1299			6	0									
	1299			7	0									14:51
MP	1266	14:52	0	0	1	1	39,13	48,5	63,84	7X7	0	1	0,475	
	1266			0	1	1	41,84	48,57	64,66	3X4	0	1	0,33	
	1266			1	1	0								
	1266			2	1	1	42,01	38,85	63,84	4X3	0	0	0,378	
	1266			2	1	1	38,64	38,66	64,42	3X3	0	0	0,313	
	1266			3	1	0								
	1266			4	0									
	1266			5	0									
	1266			6	0									
	1266			7	0									14:58
MP	1293	15:22	1	0	1	1	65,58	54,34	83,36	5x7	0	1	0,488	
	1293			0	1	1	62,33	53,4	83,69	6x5	0	1	0,541	
	1293			0	1	1	58,48	53,51	83,77	9X8	0	1	0,665	
	1293			1	1	1	70,35	50,35	84,02	6X5	0	0	0,369	
	1293			2	1	0								
	1293			3	1	1	56,81	47,06	87,54	16X30	1	0	3,058	
	1293			4	1	1	60,48	50,25	96,15	6X4	0	1	0,482	
	1293			4	1	1	62,47	51,46	96,24	3X3	0	1	0,163	
	1293			5	1	1	68,33	50,97	95,01	4X4	0	0	0,35	
	1293			6	1	1	65,34	40,44	91,73	15X23	1	0	1,483	
	1293			6	1	1	70,58	43,12	91,07	9X22	1	0	1,788	
	1293			7	1	1	60,06	44,4	89,02	4X6	1	0	0,779	
	1293			7	1	1	60,98	43,38	91,89	24X16	1	0	1,902	
	1293			7	1	1	60,51	43,48	94,51	16X13	1	0	1,137	15:45
MP	1290	12:10	2	8	1	0								
	1290			9	1	0								
	1290			10	1	1	86,29	42,45	39,88	5X5	1	0	0,433	
	1290			11	1	0								
	1290			12	1	0								
	1290			13	1	0								
	1290			14	1	0								
	1290			15	1	1	89,08	44,16	46,35	23X19	1	0	1,531	12:17
MP	1295	13:04	0	0	1	1	35,96	49,45	75,96	4X3	0	1	0,213	
	1295			0	1	1	34,35	49,88	75,96	3X3	0	1	0,18	
	1295			1	1	0								
	1295			2	1	1	34,27	40,31	76,69	4x11	0	0	0,774	12:21
	1295			3	1	0								
	1295			4	1	1	36,28	47,96	87,11	3x4	0	1	0,2	
	1295			5	1	0								
	1295			6	1	1	39,14	35,91	85,31	2x2	0	1	0,22	
	1295			7	1	0								13:14
MP	1221	13:15	1	0	1	1	84,25	38,96	81,22	5X5	0	1	0,273	
	1221			1	1	1	80,32	36,45	84,17	2X3	0	0	0,183	
	1221			1	1	1	79,98	33,09	84,17	7X13	1	0	1,741	
	1221			2	1	1	92,51	32,37	84,17	20X42	1	0	2,621	
	1221			3	1	1	94,19	31,37	79,33	4X4	0	0	0,413	
	1221			4	1	2								
	1221			5	1	2								
	1221			6	1	2								
	1221			7	1	2								13:27
MP	1238	13:28	2	8	1	1	82,67	57	54,72	3X5	0	0	0,322	
	1238			9	1	0								
	1238			10	1	0								
	1238			11	1	0								
	1238			12	1	0								
	1238			13	1	0								
	1238			14	1	1	80,55	49,68	57,83	4X4	0	0	0,375	
	1238			15	1	0								13:33
MP	1214	13:44	0	0	1	1	79,53	42,22	72,06	4x3	0	1	0,229	
	1214			0	1	1	78,49	42	73,13	4x5	0	1	0,34	
	1214			1	1	0								
	1214			2	1	0								
	1214			3	1	0								
	1214			4	1	1	79,77	39,27	84,36	6x8	0	1	0,485	
	1214			4	1	1	79,29	39,6	82,97	3x3	0	1	0,197	
	1214			5	1	1	70,1	35,51	96,25	3x3	0	1	0,321	
	1214			6	1	1	74	28,64	82,64	3x7	0	1	0,467	
	1214			7	1	0								13:55
MP	1286	13:57	3	8	1	1	65,23	58,07	40,78	5X5	0	1	0,344	
	1286			9	1	0								
	1286			10	1	1	62,67	49,82	41,11	6X6	0	0	0,406	
	1286			10	1	1	61,43	50,6	38,65	5X5	0	0	0,421	
	1286			11	1	0								
	1286			12	1	0								
	1286			13	1	0								
	1286			14	1	0								
	1286			15	1	0								14:03
MP	1236	14:13	0	0	1	1	71,22	52	79,07	5X5	0	1	0,368	
	1236			0	1	1	73,42	52,77	82,02	4X4	0	1	0,233	
	1236			1	1	0								
	1236			2	1	0								
	1236			3	1	0								
	1236			4	1	1	75,38	51,39	92,43	6X5	0	0	0,471	
	1236			5	1	1	70,59	46,47	97,68	3X3	0	1	0,235	
	1236			5	1	1	70,39	46,82	91,37	2X2	0	0	0,295	
	1236			5	1	1	70,58	45,67	89,97	4X3	0	1	0,227	
	1236			6	1	1	71,48	40,5	90,3	3X3	0	0	0,261	
	1236			7	1	0								14:25
MP	1211	14:53	2	8	0									
	1211			9	1	0								
	1211			10	0									
	1211			11	1	0								
	1211			12	1	0								
	1211			13	1	0								
	1211			14	0									
	1211			15	1	0								14:57
MP	1278	14:59	0	0	1	1	74,02	45,1	78,45	5X4	0	1	0,399	
	1278			0	1	1	77,99	44,94	78,78	3X3	0	0	0,384	

	1278			1	1	1	72,02	39,88	82,72	3X3	0	0	0,232	
	1278			1	1	1	71,44	39,12	81,65	20X13	0	0	1,333	
	1278			2	1	1	81,63	35,08	78,78	3X3	0	0	0,344	
	1278			2	1	1	76,11	35,31	80,26	5X7	0	0	0,545	
	1278			3	1	1	86,59	41,52	77,88	4X4	0	0	0,367	
	1278			3	1	1	84,14	39,35	82,23	19X52	1	0	4,056	
	1278			4	1	0								
	1278			5	1	1	71,56	35,1	87,39	7X5	0	0	0,621	
	1278			5	1	1	72,88	39,26	87,56	9X9	0	0	0,566	
	1278			5	1	1	71,23	34,53	88,62	2X2	0	0	0,217	
	1278			6	1	1	75,93	32,32	89,11	19X22	0	0	1,096	
	1278			7	1	1	82,38	36,72	88,79	4X5	0	1	0,37	
	1278			7	1	1	84,54	39,24	88,13	8X6	0	0	0,487	
	1278			7	1	1	81,22	36,69	87,47	8X6	0	0	0,488	
	1278			7	1	1	81,87	38,18	87,23	5X3	0	0	0,382	
	1278			7	1	1	80,35	37,35	85,42	5X6	0	0	0,588	15:26
MP	1263	15:28	3	8	1	1	56,41	58,87	50,02	4X3	0	1	0,206	
	1263			9	1	0								
	1263			10	1	0								
	1263			11	1	1	63,59	53,71	45,76	4X3	0	0	0,19	
	1263			12	1	0								
	1263			13	1	0								
	1263			14	1	0								
	1263			15	1	0								15:32
MP	1235	10:32	1	0	1	2								
	1235			1	1	2								
	1235			2	1	2								
	1235			3	1	2								
	1235			4	1	2								
	1235			5	1	2								
	1235			6	1	2								
	1235			7	1	2								10:33
MP	1285	10:34	0	0	1	1	80,7	45,16	78,36	3X3	0	1	0,242	
	1285			1	1	0								
	1285			2	1	0								
	1285			3	1	1	83,75	38,93	81,39	5X19	1	0	1,698	
	1285			4	1	1	80,59	42,18	89,84	2X3	1	0	0,23	
	1285			4	1	1	81,08	42,01	89,59	4X3	0	1	0,267	
	1285			5	1	0								
	1285			6	1	1	75,33	32,1	88,93	3X3	0	0	0,255	
	1285			7	1	1	83,23	37,08	90,82	26X14	1	0	1,91	10:45
MP	1233	10:47	3	8	1	1	56,68	53,68	39,8	4X5	0	0	0,289	
	1233			8	1	1	59,42	52,26	37,42	5X6	0	0	0,331	
	1233			9	1	1	48,78	53,31	42,34	6X8	0	0	0,656	
	1233			9	1	1	49,16	50,54	41,93	4X8	0	0	0,616	
	1233			9	1	1	48,19	51,03	40,95	6X11	0	0	0,964	
	1233			10	1	1	56,95	46,74	40,37	14X20	1	0	1,047	
	1233			10	1	1	50,65	47,52	41,44	10X13	1	0	0,681	
	1233			11	1	1	59,27	48,34	40,04	9X7	0	0	0,657	
	1233			12	1	0								
	1233			13	1	0								
	1233			14	1	1	57,01	45,59	44,39	15X9	1	0	1,456	
	1233			15	1	1	60	50,01	46,44	11X6	1	0	0,803	11:06
MP	1206	11:41	3	8	1	1	67,4	57,53	31,36	3x4	0	0	0,369	
	1206			8	1	1	64,2	56,45	31,44	3x3	0	0	0,263	
	1206			9	1	0								
	1206			10	1	1	63,61	49,19	28,74	4x2	0	0	0,37	
	1206			11	1	1	61,63	50,85	28,74	4x3	0	0	0,282	
	1206			12	1	0								
	1206			13	1	0								
	1206			14	1	0								
	1206			15	1	0								11:50
MP	1215	11:59	0	0	1	0								
	1215			1	1	0								
	1215			2	1	0								
	1215			3	1	1	36,07	41,22	71,57	12x10	1	0	2,044	
	1215			4	1	1	40,83	36,73	76,74	4x5	0	1	0,352	
	1215			5	1	0								
	1215			6	1	1	40,96	26,34	76,41	3x3	0	1	0,275	
	1215			7	1	1	36,77	33,21	77,56	30x42	1	0	3,092	12:13
MP	1282	15:43	1	0	1	1	58,02	52,26	94,22	3x3	0	0	0,457	
	1282			0	1	1	61,68	52,53	95,86	4x4	0	1	0,293	
	1282			1	1	1	68,56	48,97	96,03	2x3	0	0	0,193	
	1282			2	1	0								
	1282			3	1	0								
	1282			4	1	1	59,82	51,43	106,19	5x5	0	1	0,501	
	1282			5	1	1	67,12	45,96	103,73	3x3	0	0	0,188	
	1282			5	1	1	67,07	46,62	104,55	4x4	0	1	0,299	
	1282			6	1	0								
	1282			7	1	1	57,88	45,38	104,63	6x5	1	0	0,676	
	1282			7	1	1	57,27	45,83	105,61	7x7	1	0	0,516	15:57
MP	1216	15:58	2	8	1	1	85,16	55,02	52,19	4X4	0	1	0,367	
	1216			9	1	0								
	1216			10	1	0								
	1216			11	1	0								
	1216			12	1	0								
	1216			13	1	0								
	1216			14	1	0								
	1216			15	1	0								16:01
MP	1271	16:02	2	8	1	1	47,88	57,31	34,01	5X4	0	0	0,374	
	1271			8	1	1	50,68	57,25	35	3X4	0	1	0,332	
	1271			9	1	0								
	1271			10	1	0								
	1271			11	1	1	45,11	52,04	32,29	4X3	0	0	0,364	
	1271			12	1	0								
	1271			13	1	0								
	1271			14	1	0								
	1271			15	1	0								16:08
MP	1213	16:09	3	8	1	1	66,63	57,05	49,81	4X4	0	1	0,439	
	1213			9	1	0								
	1213			10	1	0								
	1213			11	1	0								
	1213			12	0									
	1213			13	0									
	1213			14	0									
	1213			15	0									16:12
MP	1251	16:13	0	0	1	1	77,32	47,2	78,27	3X3	0	1	0,291	
	1251			1	1	0								
	1251			2	1	0								
	1251			3	1	0								
	1251			4	1	1	82,78	44,56	85,9	3x3	0	0	0,211	

	1251			5	1	0								
	1251			6	1	1	76,72	33,78	88,36	3x3	0	1	0,266	
	1251			7	1	1	83,11	39,97	89,59	3x4	0	0	0,374	16:22
MP	1261	16:31	1	0	1	1	57,72	53,85	85,46	5X4	0	1	0,331	
	1261			0	1	1	59,13	53,49	85,38	4X4	0	1	0,455	
	1261			0	1	1	54,27	54,83	85,62	7X5	0	1	0,349	
	1261			0	1	1	60,44	54,36	86,53	5X5	0	0	0,415	
	1261			1	1	1	49,23	48,73	87,43	4X4	0	0	0,34	
	1261			1	1	1	49,64	51,62	86,69	5X3	0	0	0,356	
	1261			2	1	1	58,9	43,41	87,84	4X5	0	1	0,276	
	1261			3	1	1	62,36	49,4	81,44	5X3	0	0	0,421	
	1261			4	1	1	60,14	52,91	99,97	4X12	0	0	0,861	
	1261			5	1	1	51,81	50,3	99,81	3X3	0	1	0,293	
	1261			6	1	1	54,17	43,36	99,15	6X10	0	1	1,022	
	1261			7	1	1	63,56	47,93	101,12	2X3	0	0	0,169	16:48
MP	1248	16:49	1	0	1	1	58,88	50,71	76	3X5	0	1	0,346	
	1248			0	1	1	62,8	50,3	76,82	6X9	0	0	0,567	
	1248			1	1	0								
	1248			2	1	1	65,89	40,43	78,14	3X5	0	0	0,461	
	1248			3	1	1	67,42	43,3	80,35	2X9	1	0	1,142	
	1248			4	1	0								
	1248			5	1	1	56,11	44,94	87,98	7X6	0	1	0,495	
	1248			6	1	1	58,98	38,88	88,06	6X6	0	1	0,362	
	1248			7	1	0								17:01
MP	1280	10:39	2	8	1	1	76,38	53,78	45,01	4x4	0	0	0,319	
	1280			8	1	1	78,08	53,7	47,39	5x5	0	0	0,406	
	1280			8	1	1	74,07	53,22	47,72	11x20	1	0	1,336	
	1280			9	1	1	72,63	52,37	47,23	5x6	1	0	1,057	
	1280			10	1	0								
	1280			11	1	1	81,06	50,9	46,74	35x28	1	0	2,089	
	1280			12	1	0								
	1280			13	1	0								
	1280			14	1	0								
	1280			15	1	1	79,84	48,68	48,54	5x6	0	0	0,483	10:51
MP	1267	10:54	3	8	1	1	62,17	55,09	46,28	9X5	0	1	0,337	
	1267			9	1	0								
	1267			10	1	0								
	1267			11	1	0								
	1267			12	1	0								
	1267			13	1	0								
	1267			14	1	0								
	1267			15	1	0								10:58
MP	1232	10:59	1	0	1	1	59,35	45,7	77,72	5X6	0	1	0,327	
	1232			0	1	1	63,49	46,38	78,13	5X5	0	1	0,472	
	1232			1	1	1	68,56	43,29	80,35	3X4	0	1	0,299	
	1232			2	1	0								
	1232			3	1	1	54,82	40,61	78,38	2X3	0	0	0,313	
	1232			4	1	1	60,2	44,85	90,19	3X4	0	0	0,443	
	1232			4	1	1	61,6	45,38	90,27	4X9	0	0	0,468	
	1232			5	1	0								
	1232			6	1	0								
	1232			7	1	0								11:10
MP	1244	11:16	0	0	1	1	43,03	51,2	69,1	3x3	0	1	0,245	
	1244			1	1	1	50,42	45,73	71,23	4x8	0	0	0,707	
	1244			2	1	0								
	1244			3	1	1	37,23	47,54	69,76	15x19	1	0	1,587	
	1244			3	1	1	37,34	45,02	68,44	6x5	0	1	0,494	
	1244			4	1	1	43,66	50,19	80,17	8x10	0	0	0,806	
	1244			5	1	0								
	1244			6	1	1	45,45	39,72	79,19	3x6	0	1	0,443	
	1244			7	1	1	38,87	45,18	82,14	3x4	0	0	0,403	11:28
MP	1245	11:36	3	8	1	1	56,19	56,06	29,87	2X3	0	1	0,234	
	1245			8	1	1	54,69	56,72	32,17	3X3	0	0	0,365	
	1245			9	1	1	48,24	53,52	31,02	3X6	0	0	0,434	
	1245			10	1	0								
	1245			11	1	0								
	1245			12	1	0								
	1245			13	1	1	49,51	54,48	37,17	10X10	1	0	0,757	
	1245			14	1	0								
	1245			15	1	0								11:42
MP	1217	11:54	2	8	1	1	41,66	56,58	42,81	4x3	0	0	0,326	
	1217			8	1	1	41,14	56,47	41,83	2x3	0	0	0,253	
	1217			9	1	0								
	1217			10	1	1	37,29	49,17	40,03	3x2	0	1	0,149	
	1217			11	1	0								
	1217			12	1	0								
	1217			13	1	1	45,66	52,06	46,99	11x3	0	0	0,359	
	1217			14	1	1	34,87	47,36	47,65	5x4	0	0	0,31	
	1217			15	1	1	35,41	51,58	45,11	15x13	1	0	1,331	
	1217			15	1	1	35,29	54,37	48,22	17x11	1	0	1,105	12:06
MP	1241	13:50	1	0	1	1	56,6	50,38	71,19	3x3	0	1	0,249	
	1241			0	1	1	57,26	49,97	73,32	12x19	1	0	0,884	
	1241			1	1	1	51,72	45,79	73,89	17x27	1	0	1,216	
	1241			2	1	0								
	1241			3	1	1	63,06	43,9	73,15	5x11	1	0	0,699	
	1241			3	1	1	63,82	45,39	70,12	4x3	0	0	0,246	
	1241			4	1	2								
	1241			5	1	2								
	1241			6	1	2								
	1241			7	1	2								13:59
MP	1242	14:00	1	0	1	1	52,78	45,52	72,06	5X4	0	1	0,469	
	1242			0	1	1	58,68	44,12	71,97	4X3	0	1	0,272	
	1242			1	1	0								
	1242			2	1	0								
	1242			3	1	1	61,54	36,31	73,53	6X30	1	0	3,689	
	1242			4	1	0								
	1242			5	1	0								
	1242			6	1	1	53,66	31,34	82,96	2X3	0	1	0,293	
	1242			7	1	1	59,69	35,07	81,81	5X5	1	0	0,42	14:08
MP	1259	14:19	1	0	1	1	54,61	50,96	86,66	4X4	0	1	0,37	
	1259			0	1	1	58,48	49,59	86,9	7X7	0	1	0,511	
	1259			1	1	0								
	1259			2	1	0								
	1259			3	1	0								
	1259			4	1	0								
	1259			5	1	0								
	1259			6	1	0								
	1259			7	1	0								14:27
MP	1292	14:28	2	8	1	0								
	1292			9	1	0								

	1292			10	1	0								
	1292			11	1	0								
	1292			12	1	0								
	1292			13	1	0								
	1292			14	1	0								
	1292			15	1	1	29	53,27	43,46	25X37	1	0	2,868	14:32
MP	1256	14:33	2	8	1	1	76,44	53,55	54,13	3X3	0	1	0,245	
	1256			8	1	1	80,79	51,51	55,86	3X3	0	1	0,272	
	1256			9	1	0								
	1256			10	1	1	80,62	44,33	53,07	2X2	0	1	0,13	
	1256			11	1	0								
	1256			12	1	0								
	1256			13	1	0								
	1256			14	1	0								
	1256			15	1	0								14:39
MP	1281	14:40	1	0	1	1	61,69	47,75	93,48	5X6	0	1	0,434	
	1281			0	1	1	58,35	47,8	96,19	6X7	1	0	0,741	
	1281			0	1	1	59,85	48,03	93,48	3X2	0	1	0,337	
	1281			1	1	1	52,59	43,45	96,19	5X8	0	0	0,554	
	1281			2	1	1	61,63	37,88	94,14	6X6	0	0	0,535	
	1281			3	1	1	63,91	40,61	97,17	5X4	1	0	0,49	
	1281			4	1	0								
	1281			5	1	1	52,57	40,96	102,5	4X3	0	0	0,221	
	1281			6	1	0								
	1281			7	1	1	60,69	35,34	104,72	20X16	1	0	1,625	
	1281			7	1	1	62,42	37,71	103,65	5X4	0	0	0,266	
	1281			7	1	1	62,36	37,91	102,75	3X3	0	0	0,249	14:55
MP	1283	14:56	1	0	0									
	1283			1	0									
	1283			2	0									
	1283			3	0									
	1283			4	0									
	1283			5	1	0								
	1283			6	0									
	1283			7	1	0								14:57
MP	1279	15:10	1	0	1	0								
	1279			1	1	1	72,88	38,94	88,2	6x7	0	0	0,627	
	1279			2	1	1	67,13	34,73	85,49	6x5	0	0	0,392	
	1279			3	1	1	60,33	38,44	87,21	3x5	0	0	0,333	
	1279			4	1	1	62,38	44,68	95,41	6x10	0	0	0,517	
	1279			5	1	1	69,42	41,19	97,46	3x4	0	1	0,366	
	1279			6	1	1	66,86	34,26	96,97	5x5	0	1	0,537	
	1279			7	1	1	61,24	38,95	94,92	27x24	1	0	2,06	15:23
MP	1226	9:35	2	8	1	0								
	1226			9	1	1	74,89	47,9	32,17	3x4	0	0	0,497	
	1226			10	1	1	82,32	45,42	30,94	28x52	1	0	4,13	
	1226			11	1	0								
	1226			12	1	0								
	1226			13	1	1	76,96	46,28	34,55	11x10	1	0	0,606	
	1226			13	1	1	77,06	47,38	36,35	24x31	1	0	2,596	
	1226			14	1	1	80,54	42,63	36,68	27x30	1	0	2,533	
	1226			15	1	1	83,49	44,46	34,47	5x6	1	0	0,53	
	1226			15	1	1	85,23	46,01	38,57	51x23	1	0	2,023	9:51
MP	1277	10:00	3	8	1	1	61,33	53,47	40,92	4X3	0	0	0,247	
	1277			8	1	1	65,29	54,77	42,97	4X3	0	1	0,255	
	1277			8	1	1	61,03	53,3	42,72	4X3	0	0	0,258	
	1277			9	1	1	71,58	52,37	44,03	8X14	1	0	1,203	
	1277			9	1	1	72,08	53,7	41,24	3X2	0	1	0,222	
	1277			10	1	0								
	1277			11	1	0								
	1277			12	1	0								
	1277			13	1	0								
	1277			14	1	1	67,82	46,02	50,76	3X2	0	0	0,205	
	1277			15	1	0								10:12
MP	1276	10:19	2	8	1	1	43,76	57,25	32,04	4x6	0	1	0,54	
	1276			9	1	1	51,74	53,18	32,04	3x5	1	0	0,436	
	1276			9	1	1	50,91	55,09	29	5x6	0	0	0,46	
	1276			10	1	0								
	1276			11	1	0								
	1276			12	1	0								
	1276			13	1	1	50,29	53,34	34,58	7x8	1	0	1,189	
	1276			14	1	0								
	1276			15	1	0								10:27
MP	1205	10:28	3	8	1	0								
	1205			9	1	0								
	1205			10	1	0								
	1205			11	1	0								
	1205			12	1	0								
	1205			13	1	1	51,34	53,58	39,25	8X8	0	0	0,678	
	1205			14	1	0								
	1205			15	1	1	61,31	51,66	39,34	21X16	1	0	0,766	10:34
MP	1234	10:46	3	8	1	0								
	1234			9	1	1	71,04	51,71	42	4x3	0	0	0,292	
	1234			10	1	0								
	1234			11	1	1	60,6	50,05	40,69	3x3	0	0	0,164	
	1234			12	1	0								
	1234			13	1	0								
	1234			14	1	0								
	1234			15	1	0								10:50
MP	1207	10:58	1	0	1	1	59,25	49,31	76,59	4x4	0	1	0,339	
	1207			0	1	1	56,09	49,2	76,67	4x4	0	1	0,266	
	1207			0	1	1	59,9	48	80,03	3x4	0	0	0,267	
	1207			1	1	0								
	1207			2	1	0								
	1207			3	1	0								
	1207			4	1	0								
	1207			5	1	0								
	1207			6	1	0								
	1207			7	1	1	62,24	41,07	87,25	8x6	1	0	0,662	11:07
MP	1225	11:08	0	0	1	1	45,97	49,51	76	3X4	0	1	0,288	
	1225			0	1	1	43,55	51,1	84,12	13X13	1	0	1,501	
	1225			1	1	1	50	43,56	83,87	4X4	0	0	0,285	
	1225			1	1	1	50,11	44,05	80,35	11X15	1	0	1,276	
	1225			2	1	1	45,93	40,83	82,56	9X9	0	0	0,67	
	1225			3	1	1	36,81	47,62	85,68	5X11	1	0	1,463	
	1225			4	1	2								
	1225			5	1	2								
	1225			6	1	2								
	1225			7	1	2								11:18
MP	1269	11:21	0	0	1	1	46,19	50,28	69,08	4X5	0	1	0,31	

	1269			1	1	1	53,36	46,47	69,74	4X3	0	0	0,568	
	1269			2	1	0								
	1269			3	1	0								
	1269			4	0									
	1269			5	1	1	51,87	41,74	77,36	6X4	0	1	0,68	
	1269			6	0									
	1269			7	1	1	41,84	39,83	82,2	13X9	1	0	1,192	
	1269			7	1	1	41,27	40,55	80,97	12X6	0	0	0,755	11:30
MP	1231	11:31	0	0	1	1	77,38	38,81	64,15	4X2	0	1	0,22	
	1231			0	1	1	81,57	38,26	64,72	3X3	0	0	0,301	
	1231			1	1	0								
	1231			2	1	0								
	1231			3	1	0								
	1231			4	1	1	82,2	35,22	75,22	4x4	0	0	0,369	
	1231			5	1	0								
	1231			6	1	1	74,69	25,26	76,69	5x10	0	0	0,874	
	1231			6	1	1	76,17	24,67	73,58	6x5	0	0	0,49	
	1231			7	1	0								11:45
MP	1224	11:56	0	0	1	1	80,33	50,56	77,49	5x4	0	1	0,356	
	1224			1	1	1	71,81	44,12	81,75	4x4	0	0	0,41	
	1224			1	1	1	71,39	47,46	79,7	5x5	0	0	0,478	
	1224			2	1	0								
	1224			3	1	1	84,11	43,23	80,52	8x10	1	0	1,487	
	1224			4	1	2								
	1224			5	1	2								
	1224			6	1	2								
	1224			7	1	2								12:02
MP	1222	12:03	1	0	1	1	64,51	55,1	85,78	4X4	0	1	0,253	
	1222			0	1	1	65,97	54,97	85,95	3X4	0	1	0,319	
	1222			1	1	0								
	1222			2	1	0								
	1222			3	1	0								
	1222			4	1	0								
	1222			5	1	0								
	1222			6	1	1	66,47	43,17	97,59	7X9	0	0	0,579	
	1222			7	1	1	63,22	46,56	92,67	13X9	1	0	0,771	
	1222			7	1	1	60,71	48,3	96,52	14X16	1	0	1,689	12:!4
MP	1296	12:15	3	8	1	0								
	1296			9	1	0								
	1296			10	1	0								
	1296			11	1	0								
	1296			12	1	0								
	1296			13	1	1	54,96	52,76	34,96	4X3	0	0	0,295	
	1296			14	1	0								
	1296			15	1	0								12:18
