# Supplementary material for: Development of a scoring method to visually score cortical interruptions on high-resolution peripheral quantitative computed tomography in rheumatoid arthritis and healthy controls
Source: PLoS One. 2018 Jul 9;13(7):e0200331. doi: 10.1371/journal.pone.0200331 (PMC6037386; doi:10.1371/journal.pone.0200331)
Supplement: S1 Table — (DOCX) [file pone.0200331.s005.docx]

| S1 Table. Mean (SD) number of cortical interruptions based on quadrants in healthy controls and patients with RA | | | |
| --- | --- | --- | --- |
|  | Healthy controls | Patients with RA |  |
| Quadrants | mean (SD) | mean (SD) | p-value |
| Palmar | 0.6 (0.9) | 0.5 (0.9) | p=0.43 |
| Ulnar | 0.1 (0.3) | 0.3 (0.5) | p=0.03 |
| Dorsal | 0.2 (0.4) | 0.3 (0.5) | p=0.56 |
| Radial | 0.1 (0.3) | 0.5 (0.6) | p<0.001 |

Values are based on results from Reader 1, first reading and calculated on quadrant level
